# Synthesis of 3,3′-dihydroxy-2,2′-diindan-1,1′-dione derivatives for tautomeric organic semiconductors exhibiting intramolecular double proton transfer†

**DOI:** 10.1039/d3sc04125e

**Published:** 2023-10-16

**Authors:** Kyohei Nakano, Iat Wai Leong, Daisuke Hashizume, Kirill Bulgarevich, Kazuo Takimiya, Yusuke Nishiyama, Toshio Yamazaki, Keisuke Tajima

**Affiliations:** a RIKEN Center for Emergent Matter Science (CEMS) 2-1 Hirosawa Wako 351-0198 Japan keisuke.tajima@riken.jp; b SANKEN, Osaka University Mihogaoka 8-1 Ibaraki Osaka 567-0047 Japan; c Department of Chemistry, Graduate School of Science, Tohoku University 6-3 Aoba, Aramaki, Aoba-ku Sendai Miyagi 980-8578 Japan; d Tohoku University Advanced Institute for Materials Research (AIMR) 2-1-1 Katahira, Aoba-ku Sendai Miyagi 980-8577 Japan; e JEOL Ltd. Musashino Akishima Tokyo 196-8558 Japan; f RIKEN Center for Biosystems Dynamics Research 1-7-22 Suehiro-cho, Tsurumi-ku Yokohama Kanagawa 230-0045 Japan

## Abstract

To investigate potential applications of the 3,3′-dihydroxy-2,2′-biindan-1,1′-dione (BIT) structure as an organic semiconductor with intramolecular hydrogen bonds, a new synthetic route under mild conditions is developed based on the addition reaction of 1,3-dione to ninhydrin and the subsequent hydrogenation of the hydroxyl group. This route affords several new BIT derivatives, including asymmetrically substituted structures that are difficult to access by conventional high-temperature synthesis. The BIT derivatives exhibit rapid tautomerization by intramolecular double proton transfer in solution. The tautomerizations are also observed in the solid state by variable temperature measurements of X-ray diffractometry and magic angle spinning ^13^C solid-state NMR. Possible interplay between the double proton transfer and the charge transport is suggested by quantum chemical calculations. The monoalkylated BIT derivative with a lamellar packing structure suitable for lateral charge transport in films shows a hole mobility of up to 0.012 cm^2^ V^−1^ s^−1^ with a weak temperature dependence in an organic field effect transistor.

## Abstract 2

A new synthetic route for 3,3′-dihydroxy-2,2′-diindan-1,1′-dione derivatives allows the study of the intramolecular double proton transfer in the solid state, which could elucidate possible interplay between tautomerization and charge transport.

## Graphical abstract

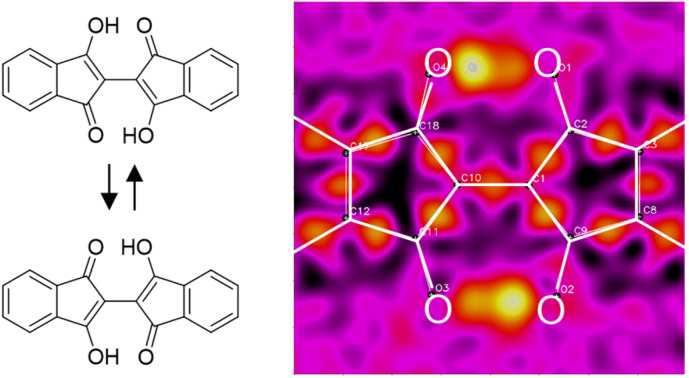

## Introduction

Among the many chemical design principles of π-conjugated molecules for high-performance organic semiconductors (OSCs), the use of intramolecular and intermolecular hydrogen bonds to control the structures and properties of the materials is particularly important.^1–4^ Intramolecular hydrogen bonds are often used to planarize the π-conjugated moieties to delocalize the electrons more widely in the molecules. The planar structure can also enhance the intermolecular charge transport through better molecular packing in the crystals and the thin films. On the other hand, intermolecular hydrogen bonds can control the packing morphology of the molecules beyond the limitations of the ordinary van der Waals interactions of the π-planes and alkyl substituents. The hydrogen bonding network can also increase the environmental stability of the materials, although it often drastically reduces the solubility of the materials in organic solvents.

**Fig. 1:**
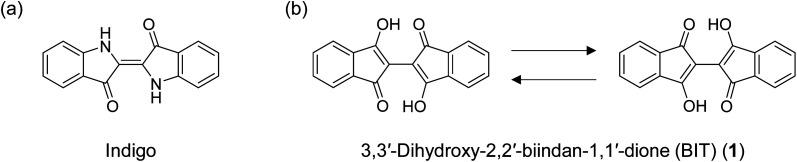
(a) Structure of indigo and (b) identical tautomeric structures of 3,3′-dihydroxy-2,2′-biindan-1,1′-dione (1).

Indigo, an organic dye with a long history, has a π-conjugated structure with intramolecular hydrogen bonds formed between two sets of N–H and C=O groups (Fig. 1a). The compound also forms an intermolecular hydrogen bonding network in the solid state, which renders indigo virtually insoluble. Recently, indigo and its derivatives have been used as OSCs with decent ambipolar mobility (0.56 cm^2^ V^−1^ s^−1^ for hole and 0.95 cm^2^ V^−1^ s^−1^ for electron) and high stability toward various environments.^5–8^ 3,3′-Dihydroxy-2,2′-biindan-1,1′-dione (BIT; 1 in Fig. 1b) has been known as a structural analogue of indigo for more than a century,^9,10^ but it has never been used as an OSC. BIT also has two strong intramolecular hydrogen bonds between the enolic O–H and C=O, resulting in the planar structure of the molecules. 1 has an intense purple color, suggesting the delocalization of electrons through π-conjugation. In addition, unlike indigo, 1 exhibits tautomerization by the simultaneous transfer of two protons from O–H to C=O to reproduce the mirror inverted hydrogen-bonded enol structures (Fig. 1b). These mobile protons within the tautomeric molecules are a unique feature of BIT compared to indigo or other hydrogen-bonded OSCs. Tautomerization of polar molecules by proton transfer in organic solids can switch the direction of the polarity and has been extensively studied in relation to the dielectric and ferroelectric properties of insulating materials.^11–16^ Even without the switching of polarity, tautomerization can also affect the molecular orbitals and the intermolecular electronic coupling in the aggregates that determine the charge transport properties in the solid state. If rapid tautomerization can be realized in OSCs, the interplay between the intramolecular proton transfer and the electronic transport could lead to a new class of OSCs with unique dynamic physical properties.^17^

To explore the possibility of BIT as an electronic functional material, we need various derivatives of the structure. However, most of the BIT derivatives reported so far involve reactions of the keto–enol structures, which loses the intramolecular hydrogen bonds and the planarity of the molecules. For example, the Wudl group reported the synthesis of zwitterionic BIT derivatives with cyanine and dione enolate structures and their application as OSCs in organic field effect transistors (OFETs) with a hole mobility of 2.1 × 10^−4^ cm^2^ V^−1^ s^−1^.^18^ To our knowledge, the use of BIT-related structures for electronic transport has been limited to this report, mainly because the available synthetic route for the BIT structure requires harsh conditions at high temperatures, and the reaction has been limited to non-substituted or symmetric and simple substitutions on the benzene rings.^19,20^

To elucidate the potential of the BIT structure in OSCs, in this study, we present a new synthetic route for BIT consisting of the addition reaction of 1,3-dione to ninhydrin followed by hydrogenation of the hydroxyl group at room temperature. This route allows us to access various BIT derivatives with the intact hydrogen bonding moieties and asymmetric substitutions. The tautomerizations involving the double proton transfer are studied both in solution and in the solid state. The possible interplay between the proton transfer and the charge transport properties is also studied by quantum chemical calculations. Some of the alkylated BIT derivatives with suitable packing structures for charge transport in films are tested in OFETs.

## Results

### Synthesis of BIT derivatives

**Fig. 2:**
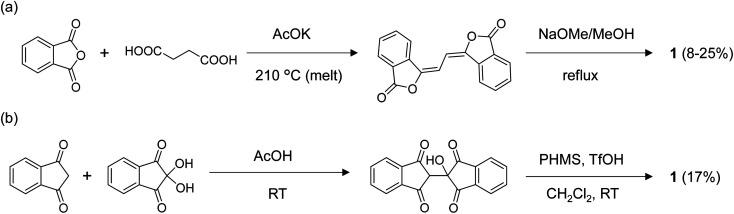
Synthetic routes for 1*via* (a) the conventional Perkin reaction and subsequent rearrangement approach and (b) the new route *via* the addition reaction and subsequent hydrogenation at room temperature proposed in the current work.

**Fig. 3:**
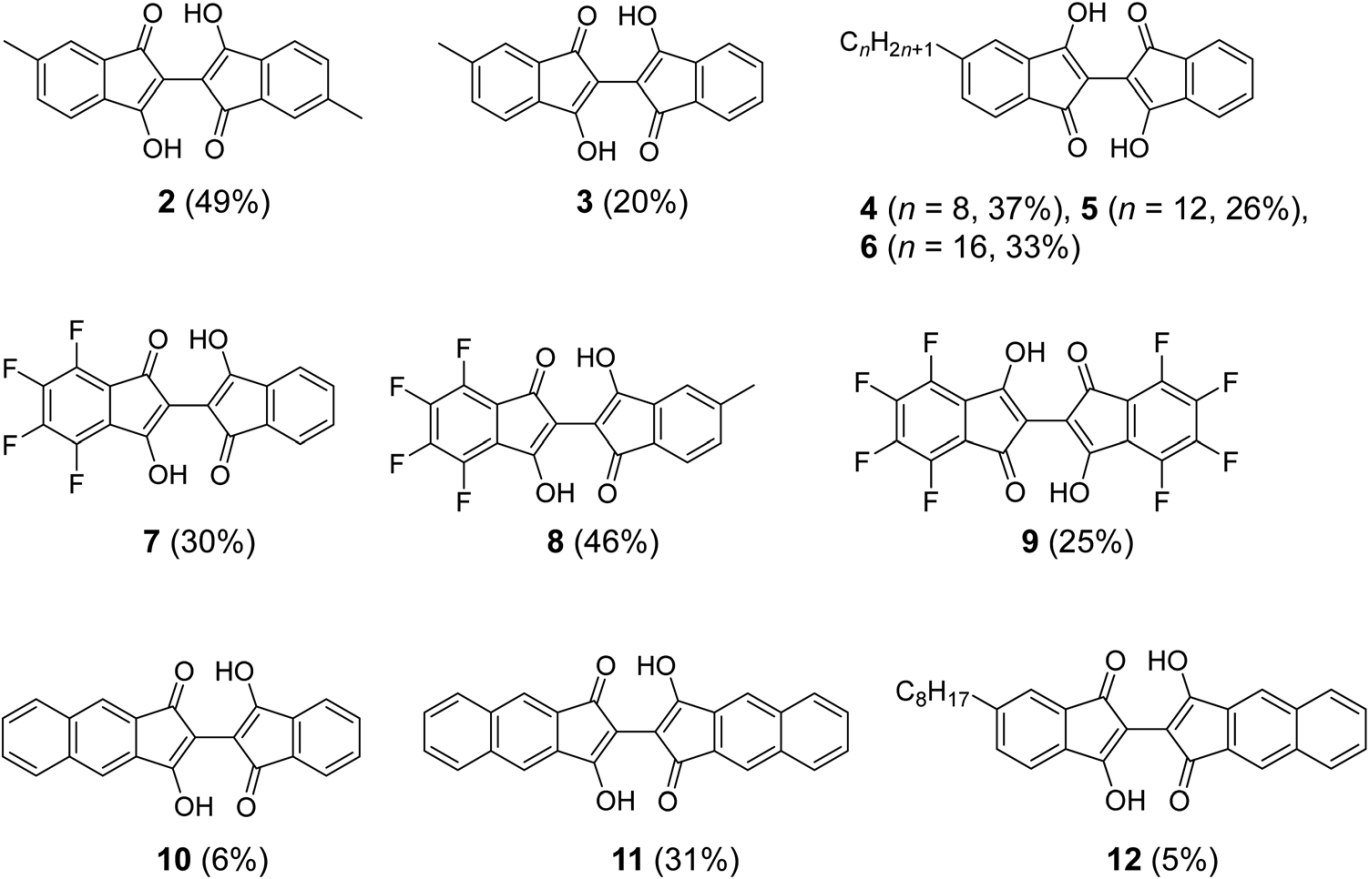
Structure of BIT derivatives (2–12) with symmetric and asymmetric substitutions synthesized by the new route shown in Fig. 2b. The numbers in parentheses are the isolated yields starting from the corresponding indan-1,3-diones and the ninhydrins.

The previously reported synthesis of 1 was *via* the Perkin reaction between phthalic anhydride and succinic acid to form the conjugated olefin, followed by the transformation to 1 with sodium methoxide (Fig. 2a).^9,21^ The first reaction step requires a high temperature (210 °C) under melt conditions, and the overall yield was low to moderate. When we repeated the synthesis, the total yield of 1 after recrystallization was only 8%, although a yield of 25% was reported by the Wudl group.^21^ The application of the same reaction conditions to other derivatives with substitutions, especially those with asymmetric structures, would be difficult due to the harsh conditions and the lack of reaction selectivity. To our knowledge, the application of this route has been limited to the syntheses of 1, and the symmetric dimethyl (2 in Fig. 3)^19^ and tetramethoxy derivatives.^20^ Moreover, we found that 1 synthesized by this route contained a small amount of a strongly fluorescent impurity, probably the structural isomers formed during the high-temperature reactions, which was difficult to remove by repeated recrystallization or sublimation. This hindered the detailed photophysical analysis of 1 and its derivatives, as we show in the Electronic properties section.

To overcome these problems, we developed the synthetic route shown in Fig. 2b. First, 2-hydroxy-2,2′-biindan-1,1′,3,3′-tetraone was synthesized by the addition reaction of indan-1,3-dione to ninhydrin in acetic acid at room temperature in moderate yields (40–60%).^22^ In the second step, the hydrogenation of –OH with poly(methylhydrosiloxane) (PMHS) catalyzed by methanesulfonic acid or trifluoromethanesulfonic acid afforded 1. Under the reductive conditions tested (*i.e.*, hydrosilanes with Et_2_O·BF_3_ or B(C_6_F_5_)_3_, Pd/C and H_2_, and formic acid^23^), PMHS/protonic acids gave the best product yields (30–70%).^24^ Et_3_SiH also worked as a hydrogen source in the combination of the protonic acids, but gave a lower yield than PMHS. Although the total yield of 1 obtained with the new route (17% after the two steps) was not substantially higher than that obtained with the Perkin route, both the reaction steps in Fig. 2b were performed at room temperature, which prevented the formation of the byproduct. The milder conditions also allowed the synthesis of BIT derivatives with various functionalities and to achieve the asymmetric substitution of BIT starting from either or both indan-1,3-diones or ninhydrins functionalized on the benzene rings.

The applicability of the new synthetic route was tested with several derivatives of indan-1,3-dione and ninhydrin. The reaction between 5-alkyl-1,3-indandione and ninhydrin afforded BIT derivatives with asymmetric alkyl substitutions (3–6 in Fig. 3). 2 was synthesized by the reaction between 5-methyl-1,3-indandione and 4-methylninhydrin to give the product as a mixture of two structural isomers (5,5′-dimethyl and 5,4′-dimethyl, see NMR Spectra in solution section below). Reactions of tetrafluoroindan-1,3-dione with ninhydrin or 5-alkylated ninhydrin afforded the corresponding asymmetrically fluorinated (7) or fluorinated and alkylated (8) derivatives. Perfluorinated BIT (9) was also synthesized from tetrafluoroindan-1,3-dione and tetrafluoroninhydrin by the same reactions but in a lower yield. This is because the tetrafluoroninhydrin could not be isolated, probably due to its low stability, and the second steps of the reactions in Fig. 2b were carried out without isolating the product from the first step. Benz[*f*]indan-1,3-dione was also used in the reactions with ninhydrin to give the asymmetric compound (10). Benz[*f*]indan-1,3-dione and benzo[*f*]ninhydrin were used to synthesize the symmetric 3,3′-dihydroxy-2,2′-bibenz[*f*]indan-1,1′-dione (11) and the asymmetric 3,3′-dihydroxy-2,2′-benz[*f*]indan-5-indan-1,1′-dione (12). The synthetic details and characterizations are presented in the ESI (Fig. S1–S6).†

Considering the large variety of indan-1,3-dione analogues available and the rich chemistry of ninhydrin,^25–27^ these results demonstrate that the new synthetic route in Fig. 2b can be used to access various substituted BIT derivatives that cannot be easily synthesized by the conventional route in Fig. 2a.

### NMR spectra in solution

**Fig. 4:**
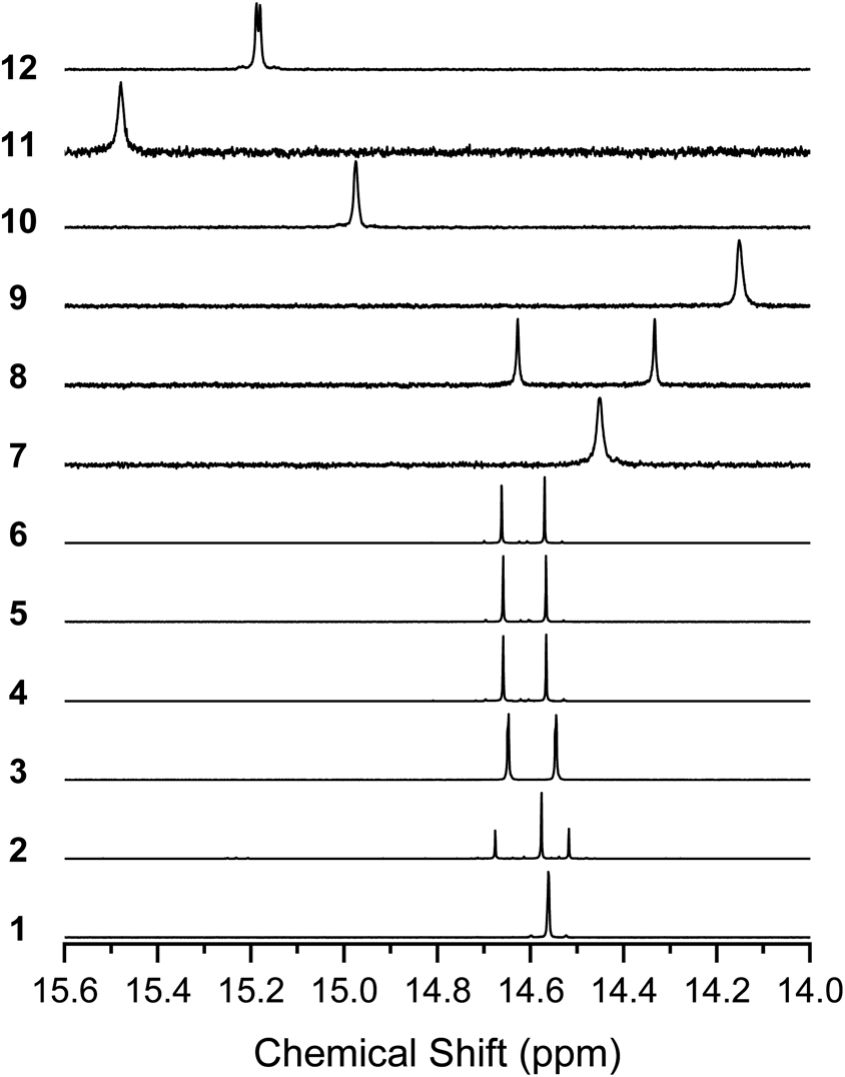
^1^H NMR spectra of 1–12 in the region of the enol –OH protons. Solvents are CDCl_3_ (25 °C) for 1–8 and 12 and 1,1,2,2-tetrachloroethane-*d*_2_ (120 °C) for 9–11.

^1^H NMR of the BIT derivatives in CDCl_3_ solution showed that the molecules were almost exclusively in their enol form at room temperature, although the coexistence of the diketo form in the spectra was observed when more polar solvents, such as TCE-*d*_2_, acetone-*d*_6_, or DMSO-*d*_6_, were used (see Fig. S7–S10† for 1). Here, we focus on the enol form of the molecules in CDCl_3_ solutions. All the synthesized BIT derivatives showed the proton peaks of the enol –OH in the low magnetic field region of 14.0–15.6 ppm, indicating that strong intramolecular hydrogen bonds between O–H and C=O were formed in the solutions (Fig. 4). Compared with 1, the fluorinated BITs showed peaks at higher magnetic fields (7–9), whereas the BITs with naphthyl groups showed peaks at lower magnetic fields (10–12), reflecting the differences in the electron densities at the enol groups. 1, 9, and 11 showed a single –OH peak, as expected from the molecular symmetry (*C*_2h_) that makes the –OH protons in the two tautomers equivalent. However, 3–6, 8, and 12 with *C*_S_ symmetry showed only two –OH peaks, although there should be four nonequivalent –OH protons if there are two tautomers. Similarly, 7 and 10 with *C*_S_ symmetry showed only one –OH peak, although there should be two nonequivalent –OH protons for the mixture of the two tautomers. These results suggest that the –OH protons are rapidly transferred between the closely facing –OH and C=O groups in the BIT molecules.

**Fig. 5:**
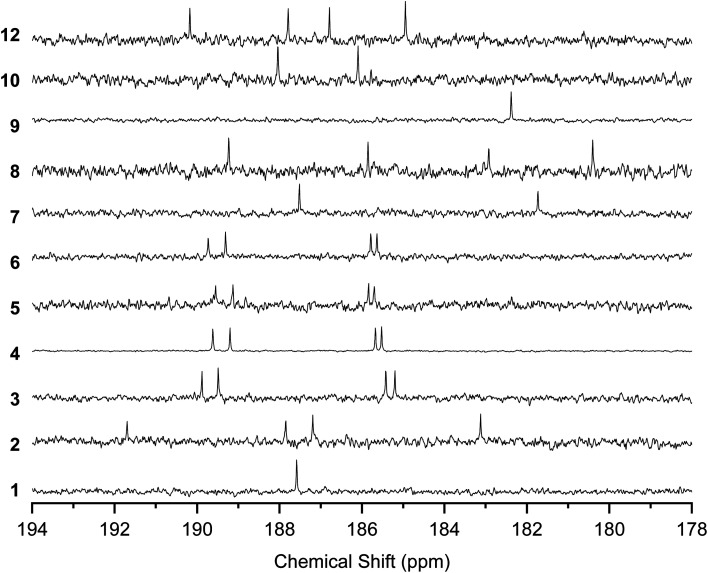
^13^C NMR spectra of 1–10 and 12 in the region of the C=O and C–OH carbons. Solvents are CDCl_3_ (25 °C) for 1–8 and 12 and 1,1,2,2-tetrachloroethane-*d*_2_ (120 °C) for 9–10.

The ^13^C NMR spectra of the solutions also indicated the rapid conversion between the tautomers by proton transfer (Fig. 5). The spectra for 1 and 9 showed a single peak at 187.58 and 182.24 ppm, respectively, corresponding to C=O and C–OH, indicating that the four carbons connected to O were equivalent. 3–6, 8, and 12 showed four peaks corresponding to C=O and C–OH, instead of the eight peaks expected for the mixture of the tautomers with nonequivalent C=O and C–OH carbons. 7 and 10 showed two peaks for C=O and C–OH, instead of four peaks expected for nonequivalent carbons with fixed –OH positions.

The ^1^H NMR spectrum of 2 shows two peaks for the 5,4′-dimethyl isomer (14.69 ppm and 14.53 ppm) and one for the 5,5′-dimethyl isomer (14.59 ppm), and the ratio of the isomers was approximately 1 : 1 based on the integrations. Our attempts to separate the isomers of 2 failed, suggesting isomerization of the molecules in solution. The formation of small amounts of the diketo tautomers is likely to cause a slow conversion between the isomers, while rotation of the central C–C bond was unlikely due to the intramolecular hydrogen bonds. In support of this, when variable temperature ^1^H NMR was performed for 4, the two –OH peaks showed no sign of coalescence up to 170 °C (Fig. S11†), suggesting that the environment for the two protons remained nonequivalent at high temperatures.

### Quantum chemical calculations of molecular properties

**Fig. 6:**
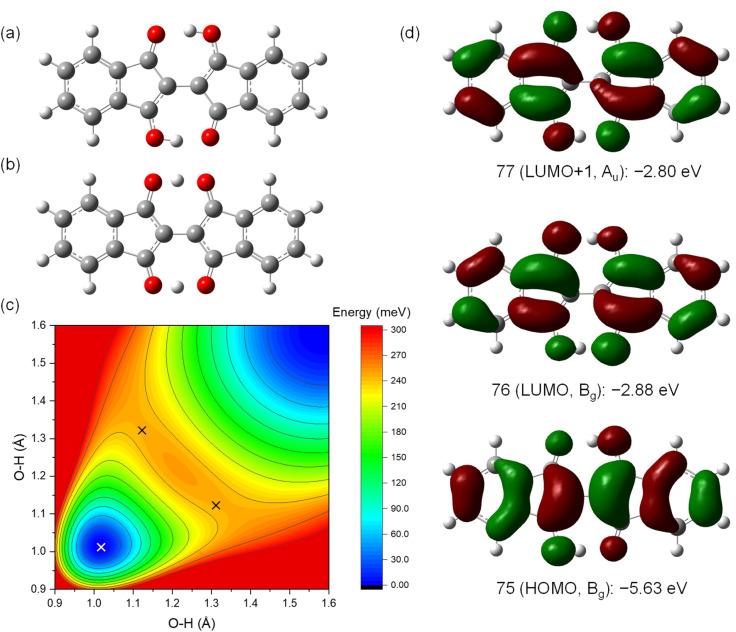
Structures of 1 in the (a) ground state and (b) transition state optimized by DFT calculations. (c) Contour plot of the potential energy for 1 with the diagonal O–H lengths as the *x*- and *y*-axes. White and black crosses indicate the ground state (set to 0) and the transition states, respectively. Note that the potential surface is a projection into the space with the two O–H lengths as the variables, so it appears asymmetric around the transition states. (d) Frontier molecular orbitals and the orbital energies for 1.

The NMR results suggested that proton transfer occurred in the BIT moiety from –OH to C=O, causing rapid tautomerization faster than the NMR timescale that made the groups equivalent in solution. The two protons in BIT could have a strongly correlated transfer because they are linked by the π-conjugation.^28,29^ To elucidate the tautomerization process, quantum chemical calculations based on density functional theory (DFT) were performed for 1 (Fig. 6). The optimized structure of 1 in the ground state was the 3,3′-dihydroxy-1,1′-dione structure with *C*_2h_ symmetry, with O–H lengths of 1.01 Å and O⋯O distances of 2.56 Å (Fig. 6a). The highest occupied molecular orbital (HOMO), the lowest unoccupied molecular orbital (LUMO), and LUMO+1 are shown in Fig. 6d with the orbital energy and the representation of point group. LUMO and LUMO+1 have similar orbital energies. The optimized structure of 1 in the transition states of tautomerization is shown in Fig. 6b. The structure has *C*_2v_ symmetry with two protons in the –OH groups were positioned off the center to one side with the shorter O–H distance of 1.12 Å. There are two mirror symmetric and energetically equivalent transition states. The barrier energy of the double proton transfer was calculated to be 0.23 eV, which is consistent with the fast proton transfer process observed in NMR at room temperature. According to the potential surface obtained by scanning the O–H distances, the two protons in BIT were in the symmetric double-well potential with the two transient states with mirror image structure (the black × marks in Fig. 6c). There is a local maximum of the potential for the structure with *D*_2h_ symmetry with the protons are in the middle of the O⋯O (O–H distance: 1.21 Å), but the energy difference from the transition states is only 3 meV, indicating that the potential surface is quite flat around the center. On the other hand, the structures with a single proton transferred to the other side have high potential energy (upper left and lower right regions of Fig. 6c). This result indicates that strong hydrogen-bond coupling occurred, allowing double proton transfer; the two protons were simultaneously transferred to the opposite C=O sites.^28,29^ Single proton transfer was unfavorable for the neutral 1 because it formed the unstable 1′,3′-dihydroxy-1,3-dione structure.

**Fig. 7:**
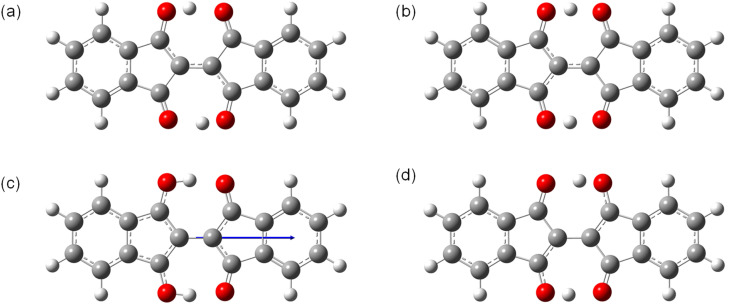
Structures of 1˙^+^ and 1˙^−^ in the (a and c) ground states and (b and d) transition states optimized by DFT calculations. The arrow in (c) shows the direction of molecular permanent dipole moment.

To investigate the properties of the charged species that would be formed in OFETs, the molecular structures of the radical cation (1˙^+^) and the radical anion (1˙^−^) are optimized in the ground and the transition states (Fig. 7). The ground state of 1˙^+^ have the structure with *C*_2h_ symmetry similar to 1 with a slightly longer O–H bond length (1.02 Å) than 1 (1.01 Å) (Fig. 7a). The transition states of 1˙^+^ is also similar to that of 1 with *C*_2v_ symmetry with off-centered protons and a shorter O–H distance of 1.08 Å (Fig. 7b). The barrier energy of double proton transfer for 1˙^+^ is 0.12 eV, which is much lower than that for 1. This implies that the protons are more mobile in the positively charged molecules than in the neutral ones. In contrast, 1˙^−^ has significantly different structures from 1 in the ground state with *C*_2v_ symmetry and the two protons positioned on one of the sides with an O–H bond length of 1.01 Å (Fig. 7c). This resulted in the formation of a large dipole moment of 3.0 Debye in the direction of the long axis of the molecules. The transition states of 1˙^−^ have *C*_2h_ symmetry with a shorter O–H distance of 1.03 Å (Fig. 7d). In contrast to 1˙^+^, 1˙^−^ has a higher barrier energy of the transition states (0.34 eV) than 1. This implies that the protons are less mobile in the negatively charged molecules.

The above calculations are also performed for 2, 3, 7, 8, 9, 10, 11, and a model of 12. The tautomers are defined as in Fig. S12–S14† and the calculated results are summarized in Tables S1–S4.† The electronic energy and the HOMO and LUMO energies are almost the same between the tautomers even for the BITs with asymmetric structures (Table S1†). The barrier energy of the double proton transfer for the neutral molecules, the radical cations, and the radical anions are in the range of 0.17–0.23 eV, 0.06–0.13 eV, and 0.33–0.46 eV, respectively (Tables S2–S4†). There was a general tendency for the barrier energy of radical anions, neutral molecules, and radical cations to be in descending order for each molecule.

### Electronic properties

**Fig. 8:**
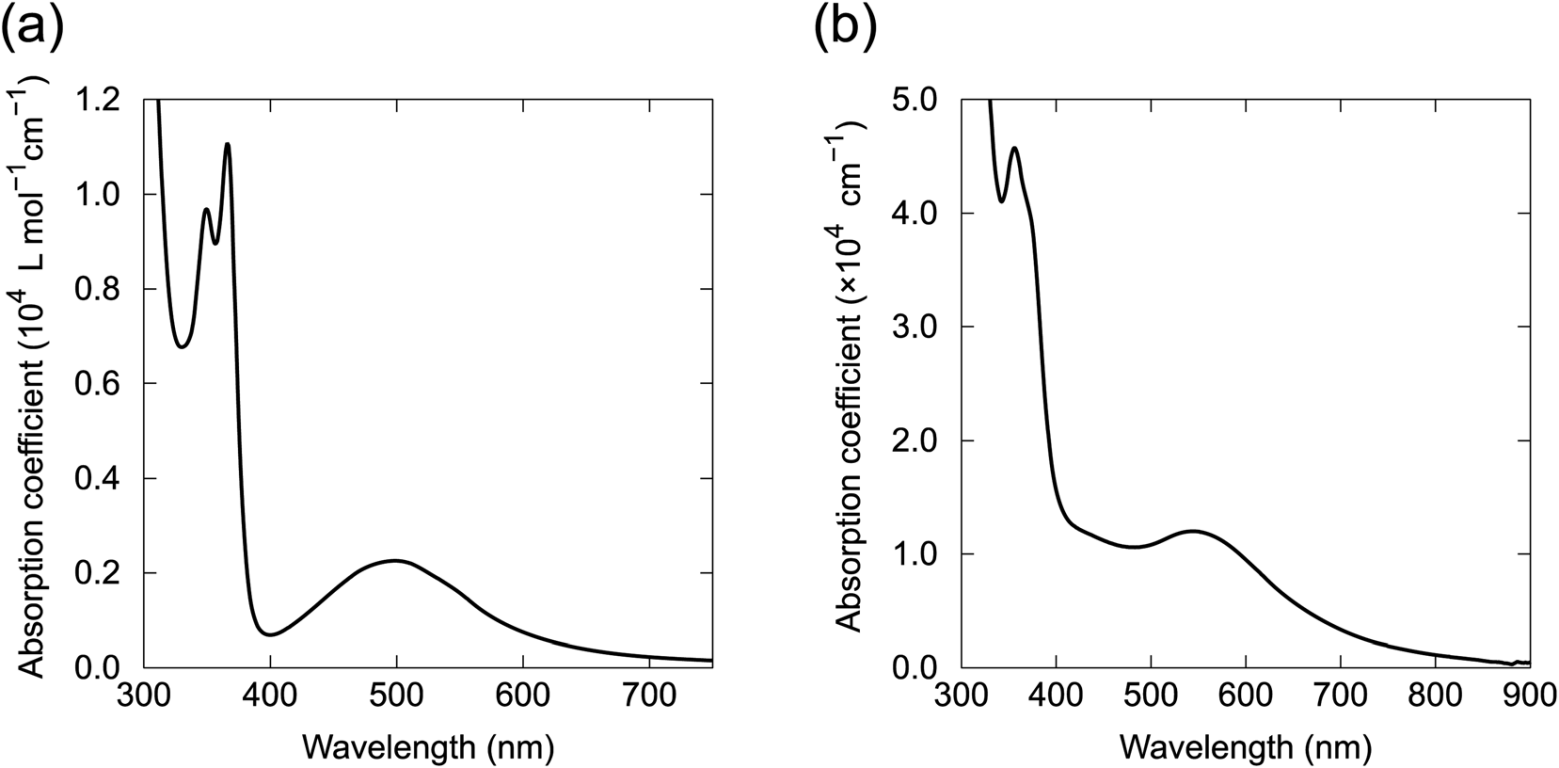
Absorption spectra of (a) 1 in CHCl_3_ solution and (b) 4 in a 100 nm-thick film prepared by vacuum deposition.

The UV-vis absorption spectrum of 1 in CHCl_3_ contained a broad absorption at 500 nm and a sharp absorption at 378 nm with vibronic features (Fig. 8a). Quantum chemical calculations for 1 based on time-dependent DFT (TD-DFT) indicate that the electronic transition from HOMO to LUMO at 610 nm was prohibited because of the symmetry of the orbitals (B_g_ → B_g_, Fig. 6d). A weak absorption was expected for the HOMO to LUMO+1 transition at 553 nm (B_g_ → A_u_), corresponding to the broad absorption band at 500 nm in the absorption spectrum (Fig. S15†). The absorption spectra of 2, 4–6, 8 and 9 in solution had almost identical absorption properties to 1 (Fig. S16†). The absorption spectrum of the vacuum-deposited 100 nm-thick film of 4 was similar to the solution spectrum (Fig. 8b). However, the film spectrum showed a red shift of the visible broad peak to 550 nm and broadening of the vibronic features for the UV absorption, which probably arose from the electronic coupling between the molecules in the solid state. Energy and oscillator strength of two lowest excited states for the molecules were calculated by TD-DFT and summarized in Table S5.†

Fluorescence spectroscopy of 1 in *o*-dichlorobenzene solution showed no observable fluorescence up to 850 nm, indicating that the BIT structure is intrinsically non-fluorescent. We speculate that the reason for the absence of the fluorescence could be related to the excited state proton transfer process in BIT, although the detailed photophysical behaviors require further investigation. In contrast, 1 synthesized with the Perkin reaction showed strong fluorescence in *o*-dichlorobenzene solution at 575 nm with vibronic features (Fig. S17†). The excitation spectra also had vibronic features that did not match the broad absorption spectrum around 500 nm. This mismatch between the absorption and the excitation spectra indicated that 1 synthesized with the Perkin reaction contained a fluorescent impurity, which was probably a structural isomer formed during the high-temperature synthesis. We failed to remove this fluorescence impurity by repeated recrystallization or vacuum sublimation. Avoiding this impurity is another advantage of our new synthetic route.

The ionization energy of 6 was 5.56 eV, determined by photoemission yield spectroscopy in air on a thin film prepared by vacuum deposition (Fig. S18†), which was consistent with the energy level of HOMO estimated by the DFT calculations (−5.63 eV). The ionization energy of BIT is deeper than that of a typical p-type OSC, such as [1]benzothieno[3,2-*b*]benzothiophene (5.45 eV)^30^ or pentacene (4.94 eV),^31^ but hole injection from the electrode is possible for BIT derivatives.

### Crystal structures

**Fig. 9:**
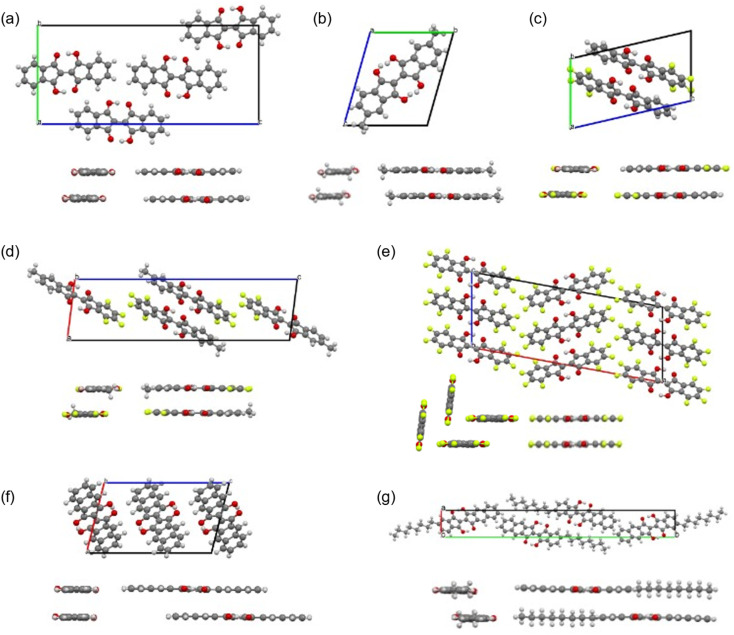
Crystal structures and intralayer packing structures along the molecular long and short axes for the BIT derivatives (a) 1, (b) 2, (c) 7, (d) 8, (e) 9, (f) 11, and (g) 12. The packing disorder in 2 and 8 and the presence of the isomers in 2 are omitted for clarity (see main text).

We obtained single crystals of 1, 2, 7–9, 11, and 12 suitable for the single-crystal X-ray structure analysis. The measurements were performed at 90 K. The packing structures in the lattices and the views along the molecular long and short axes are shown in Fig. 9, and the bond lengths of the molecules in the central part with the hydrogen bonds are summarized in Table 1. The distances between the π-planes and between the centroids of the molecules, and the displacement angle between the plane and the centroids are summarized in Table S6.† All molecules had planar structures with two intramolecular hydrogen bonds between –OH and –C=O. Although 7 and 8 have short intermolecular O⋯O distances (2.61 Å and 2.70 Å, respectively), none of the molecules shows the signature of intermolecular hydrogen bonds in the crystals, which could explain the better solubility of the BIT derivatives in organic solvents compared to indigo. The intramolecular distances between the two facing O atoms were in the range of 2.52–2.57 Å. According to the previous study, the potential curve for the protons in O–H–O is flatten by the merging of the double-well potential when the O⋯O distance is shorter than 2.55 Å.^32^ This suggests that the protons in the BIT derivatives were on the boundary between the low-barrier double-well and single-well potentials in the crystals.

**Table 1:** Atomic distances (Å) at the center of BIT molecules obtained from the single-crystal structures

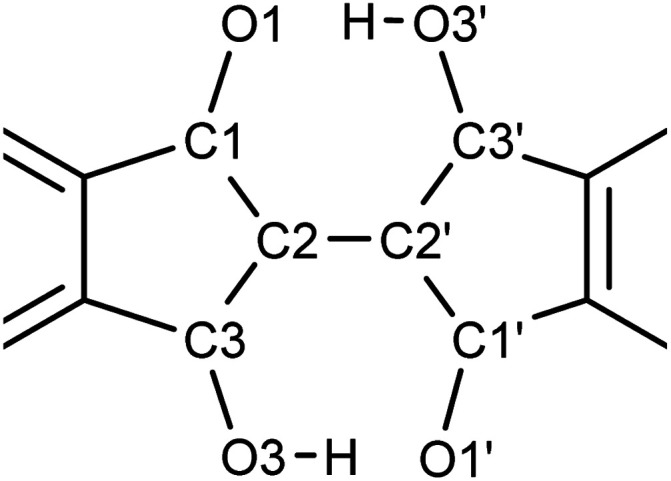							
	1	2	7	8	9	11	12
C1–O1	1.2549(9)	1.2659(18)	1.2326(5)	1.228(4)	1.231(4)	1.24750(19)	1.267(4)
C3–O3	1.3019(9)	1.2948(18)	1.3091(5)	1.306(4)	1.311(4)	1.30740(19)	1.296(4)
C1–C2	1.4482(10)	1.441(2)	1.4645(5)	1.465(4)	1.464(4)	1.4563(2)	1.441(5)
C2–C3	1.4033(10)	1.415(2)	1.3896(5)	1.385(4)	1.389(4)	1.3964(2)	1.418(5)
C2–C2′	1.4606(9)	1.454(3)	1.4549(5)	1.458(4)	1.455(6)	1.4566(3)	1.470(5)
C1′–O1′	1.2567(9)	1.2659(18)	1.2357(4)	1.238(4)	1.231(4)	1.24750(19)	1.269(4)
C3′–O3′	1.3006(9)	1.2948(18)	1.3056(5)	1.303(4)	1.311(4)	1.30740(19)	1.291(4)
C1′–C2′	1.4482(10)	1.441(2)	1.4620(5)	1.458(4)	1.464(4)	1.4563(2)	1.447(5)
C2′–C3′	1.4003(10)	1.415(2)	1.3921(5)	1.388(4)	1.389(4)	1.3964(2)	1.414(4)
O1–O3′	2.5418(9)	2.5343(18)	2.5642(4)	2.568(3)	2.548(3)	2.5162(2)	2.534(3)
O3–O1′	2.5274(8)	2.5343(18)	2.5470(4)	2.549(3)	2.548(3)	2.5162(2)	2.532(3)
O3–H	1.09(3)	0.91^a^	0.980(19)	0.95(5)	1.00(5)	1.030(7)	0.96(5)
O3′–H	1.07(2)	0.91^a^	0.985(18)	0.97(4)	1.00(5)	1.030(7)	1.03(6)

a There are two other O–H bonds (O1–H and O3–H) due to the packing disorder and the average values are presented.

In the molecular structure of 1 in the crystal (Fig. 9a and Table 1), at the first glance, the tautomeric structures and the proton positions of –OH are fixed with static conjugated double bonds; the bond lengths of C3–O3 and C1–C2 were longer than those of C1–O1 and C2–C3, respectively. However, the observed length differences between C=O and C–OH (0.045 Å) and between C=C and C–C (0.046 Å) are smaller than the calculated bond length differences in 1 by DFT (0.075 Å and 0.077 Å, respectively, see Table S7†). The observed smaller length differences suggest that there may be either dynamic or static disorder in term of the tautomeric structures in the crystal of 1.

As indicated by ^1^H NMR, 2 contained the *cis* and *trans* isomers in a 1 : 1 ratio in solution. In the single crystal, the methyl groups had an occupancy of about 0.5 at the four possible positions (not shown in Fig. 9b for clarity). This suggests that 2 shows structural disordering due to both the orientation of the molecules and the co-crystallization of the two isomers. In this case, this static packing disorder could contribute to the observed small differences in bond length between C=O and C–OH (0.029 Å) and those of C=C and C–C (0.026 Å).

In the crystal structure of 7 (Fig. 9c), the –OH protons occupied a single position, and the bond length differences between C=O and C–OH (0.074 Å) and between C=C and C–C (0.073 Å) are close to the calculated values (0.075 Å and 0.076 Å, respectively). In the molecular packing of 7, the fluorinated and non-fluorinated benzene rings were closely stacked together, suggesting the presence of donor–acceptor interactions between them. The crystal structure of 8 was also similar to that of 7 (Fig. 9d), with a dominant contribution of one of the tautomers and similar packing motifs, but there was disorder in the molecular arrangement in the direction of the methyl substituents, with the two directions present in approximately a 1 : 1 ratio.

The crystal structure of 9 was a layered structure with in-plane molecular packing in a herringbone pattern (Fig. 9e), and the contribution from one of the tautomers was dominant as for 7 and 8 at 90 K. The distance between π-planes was the shortest among the compounds (3.13 Å, Table S6†). In the crystal structure of 11 (Fig. 9f), the intramolecular O⋯O distance was the shortest among the compounds (2.52 Å, Table 1), and the bond length differences between C=O and C–OH (0.060 Å) and between C=C and C–C (0.060 Å) are slightly smaller than the calculated values (0.069 Å and 0.068 Å, respectively). The molecular packing was a pitched π-stacking structure with a large slip in the long axis direction. This structure was similar to the crystal of indigo, although unlike indigo, there are no intermolecular hydrogen bonds in 11.^33^

The crystal structure of 12 was a layered structure with antiparallel alignment of the molecules in in-plane molecular packing (Fig. 9g). The bond length differences between C=O and C–OH (0.026 Å) and between C=C and C–C (0.028 Å) are smaller than the calculated values (0.071 Å and 0.070 Å, respectively). The distance between π-planes was 3.41 Å, but the overlap of the π-conjugated core in the crystal is small due to the large displacement.

We are interested in whether the double proton transfer also occurs in the solid state. To investigate the dynamic disorder due to tautomerization, the single-crystal X-ray analysis was performed at different temperatures and the changes in bond length are analyzed. The results for 1 and 7 are summarized in Tables S8 and S9,† respectively. As the measurement temperature is increased from 90 K, the bond length differences between C=O and C–OH and between C=C and C–C become smaller (0.015 Å and 0.013 Å, respectively, for 1 at 373 K) to give a more symmetrical structure around the central part of the molecules. This result suggests that the dynamic tautomerization process occurred in the solid state to give the averaged structure of two tautomers in the X-ray analysis.

**Fig. 10:**
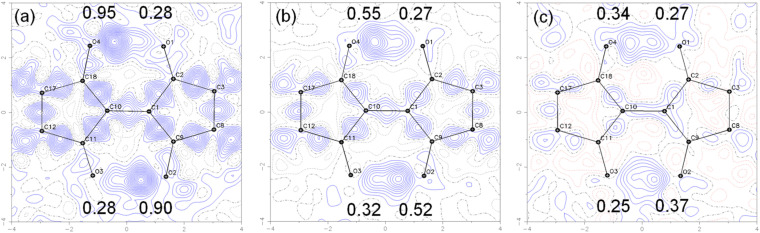
Contour plots of the difference Fourier maps for the molecular plane of the central part of 7 calculated using the structure model without the H atoms on the –OH groups and the measured diffractions at (a) 90 K, (b) 298 K, and (c) 428 K. The contours were drawn at 0.05 e Å^−3^ intervals. The numbers in the maps show the electron density maxima between the O⋯O groups in e Å^−3^, indicating the position of the –OH protons. Variable temperature magic angle spinning ^13^C solid-state NMR.

To visualize the position of the hydrogen atom more directly, the electron density distribution was examined by taking the difference Fourier maps on the plane average calculated using the structure models without the H atoms on the –OH groups and the measurements, where the protons appear as positive electron density. The results for 7 are shown in Fig. 10. The electron density difference corresponding to the proton in –OH is observed at the two positions between the O⋯O with the electron density ratio of about 77 : 23 at 90 K. When the measurement temperature was increased to 298 K and 423 K, the density ratio of the proton changed to 65 : 35 and 58 : 42, respectively. These results indicate that the tautomerization occurred in the solid state by intramolecular proton transfer. The similar tendency was observed for 1 in variable temperature measurements (Fig. S19†). The molecular *C*_2h_ symmetry of 1 leads to the symmetric double-well potential for the proton as shown by the DFT calculations. However, we speculate that the molecular packing in the single crystals makes the double well potential asymmetric. This leads to the distorted population ratio of the tautomers at thermal equilibrium depending on the temperatures according to the Boltzmann distribution. The energy difference between the tautomers in the crystal of 7 was calculated to be about 1.4 kJ mol^−1^ from the population ratios at 298 K and 423 K, assuming that the system had reached thermal equilibrium.

**Fig. 11:**
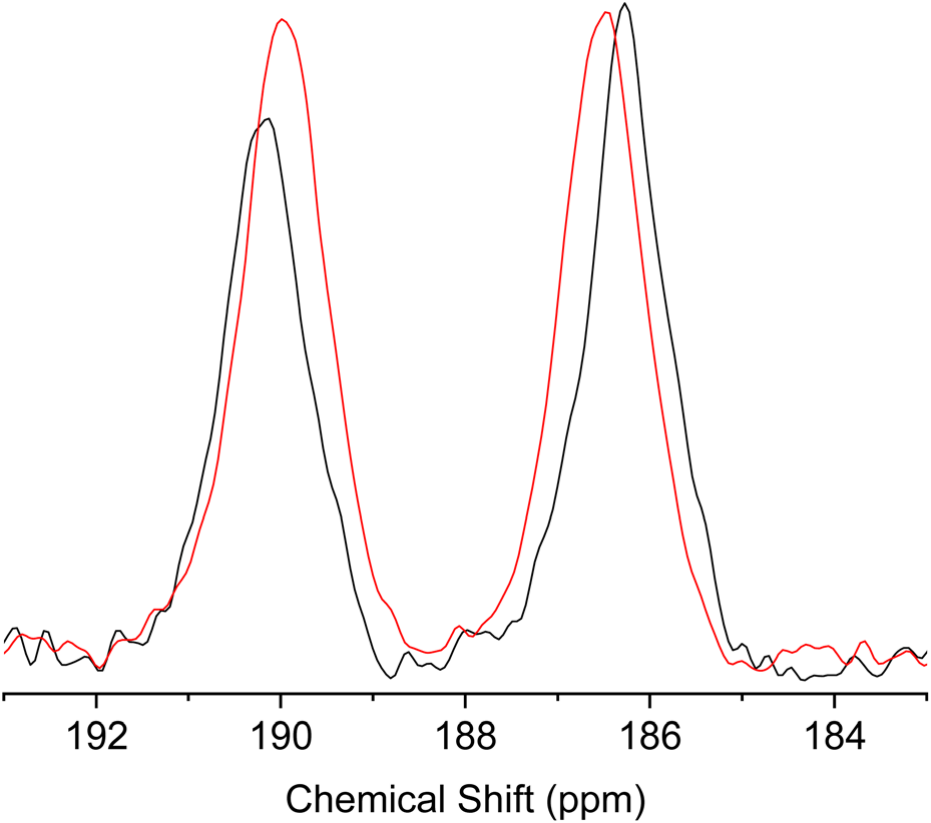
Region of C=O and C–OH peaks in the magic angle spinning (MAS) ^13^C solid-state NMR spectra of 1 measured at 298 K (black) and 423 K (red).

To further elucidate the dynamic tautomerization process in the solid state, we have performed magic angle spinning (MAS) ^13^C solid-state NMR of 1 (the full spectra are shown in Fig. S20 and S21†). The spectrum shows two peaks at 190.15 ppm and 186.28 ppm at 298 K, which can be assigned to C=O and C–OH, respectively (Fig. 11). To estimate the chemical shift difference in the solid state with the fixed tautomeric structures (at 0 K), we have performed calculations based on the Gauge Including Projector Augmented Waves (GIPAW) method, using the crystal structure of 1 as the starting structure.^34–36^ The details of the calculations are given in the ESI.† The results show that the difference in the chemical shifts between C=O and C–OH in the crystal is expected to be 15.64 ppm. The observed chemical shift difference (3.87 ppm) is much smaller than the calculated value, indicating that the two ^13^C environments are rapidly exchanged by tautomerization. The chemical shift difference reflects the biased population ratio of the tautomers at thermal equilibrium. Using the calculated chemical shift difference as the reference for the fixed tautomeric structures with an estimated error of ±2 ppm,^34^ the population ratio of the tautomers can be calculated from the observed values to be in the range of 0.64 : 0.36 and 0.61 : 0.39 at 300 K and the energy difference between two tautomers can be estimated to be 1.1–1.4 kJ mol^−1^. These results were in agreement with the population ratios estimated from the single-crystal X-ray analysis. When the measurement temperature was increased to 423 K, the difference in the chemical shift became smaller (Fig. 11), indicating the change in the population ratio of the tautomers according to the Boltzmann distribution. These results further support the picture that the fast double proton transfer in 1 occurs above the room temperature within the asymmetric double well potential induced by the crystal structure.

### Quantum chemical calculations for effects of tautomerization on charge transport

**Fig. 12:**
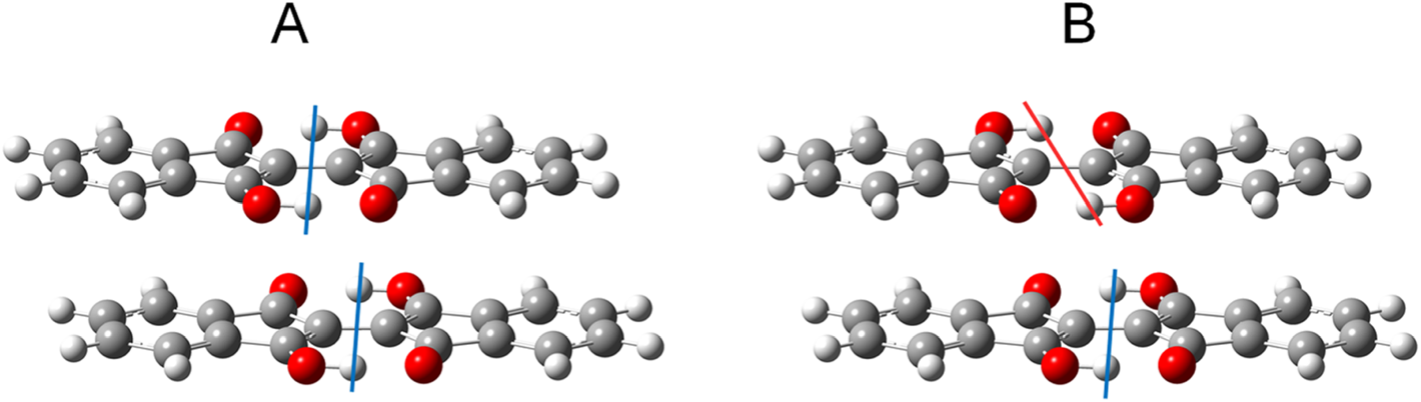
Two tautomeric situations A and B used to calculate the transfer integrals in the pair of 1. The colored lines indicate the difference in proton positions. See the ESI† for details on the procedures used to generate the structures.

To study the effects of tautomerization on charge transport in the solid state, quantum chemical calculations were performed based on the single crystal structures. The transfer integrals between two pairs of 1 with the different tautomeric situations in Fig. 12 were calculated and compared to see the effects of the tautomerization on the transfer integrals. The details of the calculation method are presented in the ESI.† When the two tautomeric situations of the pair of 1 are compared, the significant changes in the transfer integrals of 33% (A: 46 meV and B: 68 meV) and 38% (A: 13 meV and B: 18 meV) were observed for the hole and electron, respectively. This result indicates that the tautomerization in the solid state has the potential to affect the charge transport by altering intermolecular couplings.

1 has a relatively large reorganization energy of 0.41 eV for the hole. This could be attributed to the structure change of BIT with the slight shift of the proton positions in 1˙^+^ (Fig. 7a). On the other hand, the reorganization energy of 1 for the electron is very large, about 1.07 eV. This could be attributed to the large structural change in the ground state of 1˙^−^ in which the two protons are shifted to one side of the molecules as shown in Fig. 7c. Therefore, it is expected that the electron transport in BITs could be strongly prohibited.

The calculations of the transfer integrals for the pairs and the reorganization energy are also performed for 2, 7, 8, 9, and 11, and the results are summarized in Fig. S22 and Tables S10–11.† The difference in the transfer integrals between the two tautomeric situations was observed for most of the molecules (7–38%). The large reorganization energy for the charge transfer (0.36–0.48 eV for the hole and 0.96–1.24 eV for the electron) is a common feature in the BIT derivatives.

### X-ray diffraction of thin films

**Fig. 13:**
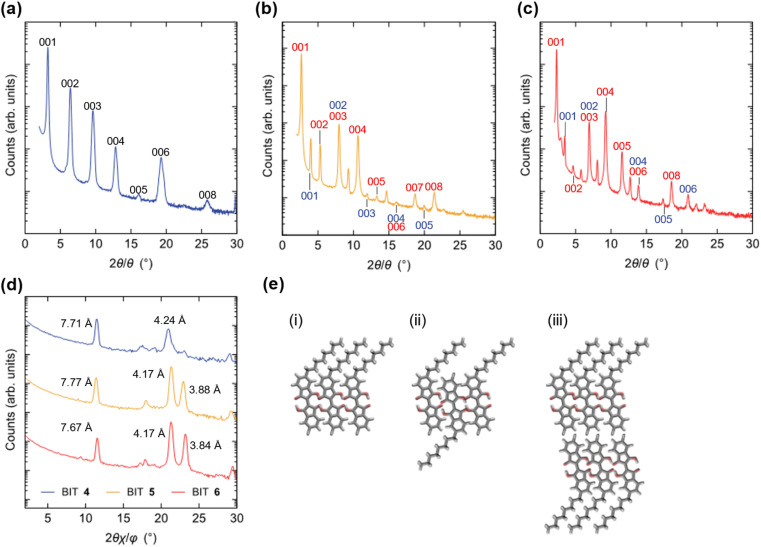
Out-of-plane X-ray diffraction patterns for the vacuum-deposited thin films of (a) 4, (b) 5, and (c) 6 with the indices of the layered structures. (d) In-plane X-ray diffraction patterns for films of 4–6. (e) Schematic representations of the possible molecular orientations in the lamellar structure for a (i) monolayer, (ii) monolayer (up and down molecular orientations), and (iii) bilayer.

For monoalkyl substituted BITs, we obtained the single crystal structure only for 4, but with a low *R* value (Fig. S23†). The result suggests a layered structure consisting of the antiparallel packing of the paired π-stacking in the layers. To elucidate the packing structure of 4–6 in thin films, X-ray diffraction patterns were measured for the thin films prepared by vacuum deposition to elucidate the structures in the solid state (Fig. 13a–d). There were several diffraction peaks in the out-of-plane direction of the films, most of which were assigned to 00*l* of the lamellar structures. The possible assignments are shown in Fig. 13 and the positions of the peaks are summarized in Table S9 in the ESI.† For the film of 4, all the peaks were assigned to the higher-order diffraction in the lamellar structure with a spacing of 2.74 nm for the 001 reflection, which was longer than the molecular length of 4 (1.77 nm) but shorter than twice its length (3.54 nm). This suggests that the structure may be a monolayer structure with mixed up and down molecular orientations in the layer (Fig. 13e(ii)). The films of 5 and 6 had more complicated diffraction patterns but most of the peaks could be assigned to two lamellar structures with different spacings for the 001 reflections of 3.31 and 2.22 nm for 5 and 3.80 and 2.55 nm for 6. Thus, based on the calculated molecular lengths of 5 (2.12 nm) and 6 (2.46 nm), the molecular orientation and packing structure of 5 and 6 were probably a mixture of the monolayer (Fig. 13e(i)) and the monolayer with up and down molecular orientations (Fig. 13e(ii)). In general, the bilayer structure in Fig. 13e(iii) may be the most suitable for the lateral charge transport in OFET devices but 4–6 did not adopt this structure in the vacuum deposited films. The in-plane diffraction patterns showed that the BIT units in the layer had a packing order in the lateral direction, although the type of packing was unclear (Fig. 13d). The diffraction peaks with the smallest spacings in this region, which were at 0.388 nm for 5 and 0.384 nm for 6 although the corresponding peak for 4 was small, could be attributed to the face-to-face π–π stacking of BIT in the layer.

### Organic field effect transistors

**Fig. 14:**
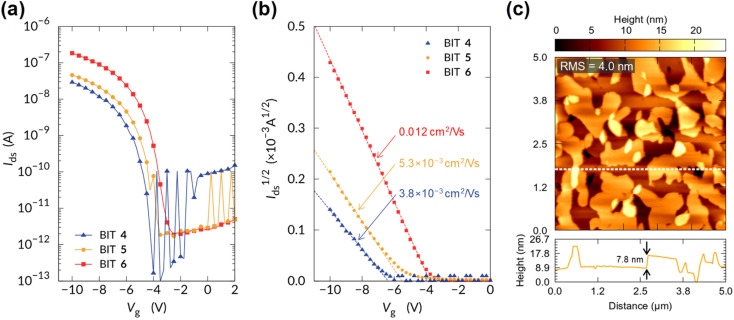
Transfer characteristics of OFETs based on the vacuum-deposited films of 4–6 with (a) logarithmic and (b) square root drain-source current scales. (c) Atomic force microscopy topographic images of the thin film of 6. Image size: 5 × 5 μm. The bottom panel shows the line profile along the dashed line in the image. RMS: root mean square.

For the BITs without long alkyl chains, such as 1 and 10, the electronic measurements on the thin films failed because they grew needle-like crystals on the substrate, forming non-uniform thin films. In contrast, the monoalkyl-substituted BITs 4–6 formed uniform thin films by vacuum deposition, and their semiconductor properties in OFETs were investigated by using the bottom-gate-top-contacts structure. Si substrates with 100 nm SiO_2_ were used after surface treatment with octadecylsilane and MoO_3_/Ag was used for the top-contact electrodes. The channel width and length were 1000 and 40 μm, respectively. All the organic films were vacuum-deposited to form uniform films and annealed at 120 °C for 30 min. The transfer characteristics of the OFETs in the saturated regime are shown in Fig. 14a and b with logarithmic and square root drain-source current scales, respectively (the output curves are shown in Fig. S24†). The monoalkyl-substituted BITs 4–6 functioned as p-type semiconductors with clear on/off switching behavior. The calculated field-effect hole mobilities increased with the alkyl chain length, and 6, which had the longest hexadecyl chain, showed a maximum mobility of 0.012 cm^2^ V^−1^ s^−1^ (Fig. 14b). This may reflect the highly ordered lamellar structure and in-plane packing of 6, as suggested by the X-ray diffraction results.

The surface morphology of the film of 6 observed by atomic force microscopy (AFM) is shown in Fig. 14c. A step and terrace structure with a height of about 7.8 nm was observed, which was almost twice the spacing of the 001 reflection in the out-of-plane direction (3.8 nm). The lateral sizes of the steps and the terraces were about 1 μm, which was small compared to those of state-of-the-art small-molecule semiconductors with high mobility (>10 μm).^37^ AFM images for the vacuum deposited films of 1, 2, 4, 5, 9, 11, and 12 are shown in Fig. S25.† The films of 1 and 2 consist of large needle-shaped crystals with a length of several μm, and the poor connectivity of the domains may not be suitable for OFET applications. The films of 4 and 5 showed step-terrace features on the surface as in 6, suggesting the formation of uniform layered structures. The films of 9, 11 and 12 show the presence of crystalline grain structures without clear step-terrace features, which may be related to the lack of charge transport properties in OFETs. Further optimization of the film structures may be necessary to improve the charge mobility of BITs in OFETs.

The temperature dependence of the hole mobility was studied for 6 in OFETs. In the range of 25 °C and 90 °C, the mobility showed a weak dependence on temperature; the higher the temperature, the lower the hole mobility (Fig. S26†). This is in contrast to typical thermally activated charge transport in disordered OSCs, where the charge hopping is activated by thermal energy. Whether this behavior is related to the interplay between intramolecular double proton transfer and charge conduction is unknown at this time and will be investigated in the future. The device showed severe degradation above 100 °C due to evaporation of the films in vacuum.

## Conclusion

We present a new synthetic route for BIT derivatives to demonstrate their application as OSCs in OFET devices. All BIT derivatives exhibit fast intramolecular double proton transfer in solution. BITs also exhibit the double proton transfer in the solid state, but due to the asymmetric potential of the protons in the solid state, the population ratio of the tautomers is biased to one side at thermal equilibrium. The rate of double proton transfer is faster than the time scale of NMR, although the exact dynamics is unclear. Further analysis of the dynamics of tautomerization is required. The tautomerization process can be further controlled by chemical modifications or an external stimulus such as heat or light. BITs could be a unique platform to study the possible interplay between proton transfer and charge transport as a first step towards solid-state proton-coupled charge transport.

## Data availability

All the data are available in the main text and ESI.†

## Author contributions

Tajima conceived the idea of the study and performed the syntheses, the characterizations, and the basic quantum chemical calculations. KN performed the characterizations of the materials and the devices. IWL contributed to the early synthesis and characterizations. DH performed the crystallographic analysis. KB and Takimiya performed the quantum chemical calculations on the transport properties. YN and TY performed the solid-state NMR measurements and the analyses. All authors critically reviewed and revised the manuscript draft and approved the final version for submission.

## Conflicts of interest

There are no conflicts to declare.

## Supplementary Material

SC-014-D3SC04125E-s001

SC-014-D3SC04125E-s002
