# Supplementary material for: Synthesis of 3,3′-dihydroxy-2,2′-diindan-1,1′-dione derivatives for tautomeric organic semiconductors exhibiting intramolecular double proton transfer
Source: Chem Sci. 2023 Oct 16;14(43):12205–18. doi: 10.1039/d3sc04125e (PMC10631252; doi:10.1039/d3sc04125e)
Supplement: SC-014-D3SC04125E-s001 [file SC-014-D3SC04125E-s001.pdf]

Electronic Supplementary Material (ESI) for Chemical Science.
This journal is © The Royal Society of Chemistry 2023

Supporting Information

Synthesis of 3,3′-Dihydroxy-2,2′-diindan-1,1′-dione Derivatives for Tautomeric Organic Semiconductors Exhibiting Intramolecular Double Proton Transfer

Kyohei Nakano[1], Iat Wai Leong[1,2], Daisuke Hashizume[1], Kirill Bulgarevich[1], Kazuo Takimiya[1,3,4], Yusuke Nishiyama[5], Toshio Yamazaki[6], Keisuke Tajima[1,*]

[1]RIKEN Center for Emergent Matter Science (CEMS), 2-1 Hirosawa, Wako 351-0198, Japan, [2]SANKEN, Osaka University, Mihogaoka 8-1, Ibaraki, Osaka 567-0047, Japan, [3]Department of Chemistry, Graduate School of Science, Tohoku University, 6-3 Aoba, Aramaki, Aoba-ku, Sendai, Miyagi 980-8578, Japan, [4]Tohoku University Advanced Institute for Materials Research (AIMR), 2-1-1 Katahira, Aoba-ku, Sendai, Miyagi 980-8577, Japan, [5]JEOL Ltd., Musashino, Akishima, Tokyo, 196-8558 Japan, [6]RIKEN Center for Biosystems Dynamics Research, 1-7-22 Suehiro-cho, Tsurumi-ku, Yokohama, Kanagawa 230-0045

Experimental

Materials

Ninhydrin, SeO_2, and $Pd(PPh_3)_2Cl_2$ were purchased from Nacalai Tesque (Japan). 1,3-Indandione, 5-methyl-1,3-indandione, 6-methyl-1-indanone, 4-bromophthalic anhydride, tetrafluorophthalic anhydride, octylboronic acid, 1-dodecyne, 1-hexadecyne, *tert*-butyl acetoacetate, ethyl acetoacetate, trifluoromethanesulfonic acid, 2,3-naphthalenedicarboxylic anhydride, 1,2-bis(dibromomethyl)benzene, and cyclopent-2-en-1-one were purchased from TCI (Japan). Poly(methylhydrosiloxane) was purchased from Alfa Aesar (USA). All chemicals and solvents were used without further purification unless otherwise noted.

Instruments

Microwave heating was performed with a 400 W microwave reactor (Initiator+, Biotage). ^{1}H, $^{19}F\{^{1}H\}$, $^{13}C\{^{1}H\}$, and $^{13}C\{^{1}H, {}^{19}F\}$ NMR spectra were recorded on an NMR spectrometer (JNM-ECZ400, JEOL) at 399 MHz for ^{1}H, 376 MHz for ^{19}F, and 100 MHz for ^{13}C. Chemical shifts are reported in delta (δ) units, part per million (ppm) relative to the reference. Tetramethylsilane was used as the internal reference for ^{1}H and ^{13}C NMR in $CDCl_3$, and the corresponding residual solvent peaks were used for the other

experiments.

High-resolution mass spectroscopy with field desorption (FD; JMS-T100GCV, JEOL) and electrospray ionization (ESI; Synapt G2, Waters) was performed.

Single-crystal X-ray diffraction data were collected on a 4-circle diffractometer (AFC-11, Rigaku) equipped with a detector (HyPix6000HE, Rigaku). The measurement temperature was controlled by blowing cool N_2 gas onto the sample. The structure was solved by the dual space method with the SHELXT-2018 program[1] and refined by the full-matrix least-squares method on F^2 with the SHELXL-2019 program.[2] Differential scanning calorimetry (DSC) was performed on a calorimeter (DSC 8230, Rigaku) with a heating/cooling rate of 10 °C min^{-1} under an N_2 flow. Al_2O_3 was used as a reference compound.

Magic angle spinning (MAS) ^{13}C solid-state NMR was performed on an NMR spectrometer (JNM-ECZ600R, JEOL) equipped with a 3.2 mm double-resonance MAS probe (JEOL) at 14.01 T with a ^{1}H resonance frequency of 599.7 MHz. Approximately 25.0 mg of sample was loaded into the 3.2 mm MAS rotor. All experiments were conducted at a MAS rate of 15-18 kHz.

Quantum chemical calculations

The geometry optimization of the molecules was conducted with the Gaussian16

program based on density functional theory (DFT) by using B3LYP hybrid density functionals with the 6-311++G(d,p) basis set with tight convergence criteria. The structure of the transition state between the two ground states with the mirror image structures was optimized with the QST3 option. The UV-vis absorption spectrum was calculated based on time-dependent DFT by single-point energy calculation of the excited states with the optimized ground state structure. The potential surface of the proton transfer process was calculated with the 6-31G(d) basis set by scanning the O-H distance in the range from 0.900 to 1.600 Å with a step of 0.007 Å and optimizing the structures at each point.

The transfer integrals were calculated based on the obtained crystal structures by using Amsterdam Density Functional (ADF) software package at PW91/TZP level of theory. First, transfer integrals between the neighboring molecular pairs in the crystals are calculated for the hole and the electron transports and the pairs with the largest integrals are chosen as in Figure S22 (Crystal). Next, the molecules with the optimized conformation by DFT calculations were placed at the positions of the selected pairs with the same center of mass and molecular plane. Two possible situations are generated in term of the choice of the tautomers as shown in Figure S22 (A and B). Finally, the difference of the transfer integral between the two pairs was calculated to see the effect

of the tautomerization. The reorganization energy of the molecules was calculated with the Gaussian16 program at B3LYP/6-311++G(d,p) level. The results are summarized in Table S10.

The geometric optimizations and chemical shift calculations in the solid-state were performed using the density functional theory programs pw and qe-gipaw in the Quantum ESPRESSO package version 7.0. The initial structures of **1** with different tautomeric structure are constructed from the obtained crystal structure by placing the corresponding tautomer in the same positions and orientations. The structures were relaxed with fixed cell constants using the pseudopotentials of X.pbe-tm-new-gipaw-dc.UPF. The kinetic energy cutoff for the wavefunctions (ecutwfc) was set to 80 Ry. The difference in the total electronic energy between the tautomeric structures was 2.0 kJ/mol.

Field-effect transistor device fabrication and characterization

The surface of a precleaned n+Si substrate with a 100 nm SiO_2 insulating layer (SEIREN KST, Japan) was treated with a solution of octadecyltrimethoxysilane (TCI) in trichloroethylene to form a self-assembled monolayer, as previously reported.[3] On the substrate, BITs were thermally evaporated under a pressure of 10^{-3} Pa with a deposition rate of 0.5 Å/s. A 10-nm-thick MoO_3 and 50-nm-thick Ag top-contact electrode were

formed sequentially by thermal evaporation through a metal shadow mask under a pressure of 10^{-4} Pa. The channel width and length were 1000 and 40 μm, respectively. Source meters were used to measure the source-drain current (6430, Keithley) and gate leakage current (2400, Keithley). Field-effect transistor measurements were conducted in a vacuum of ~10^{-2} Pa.

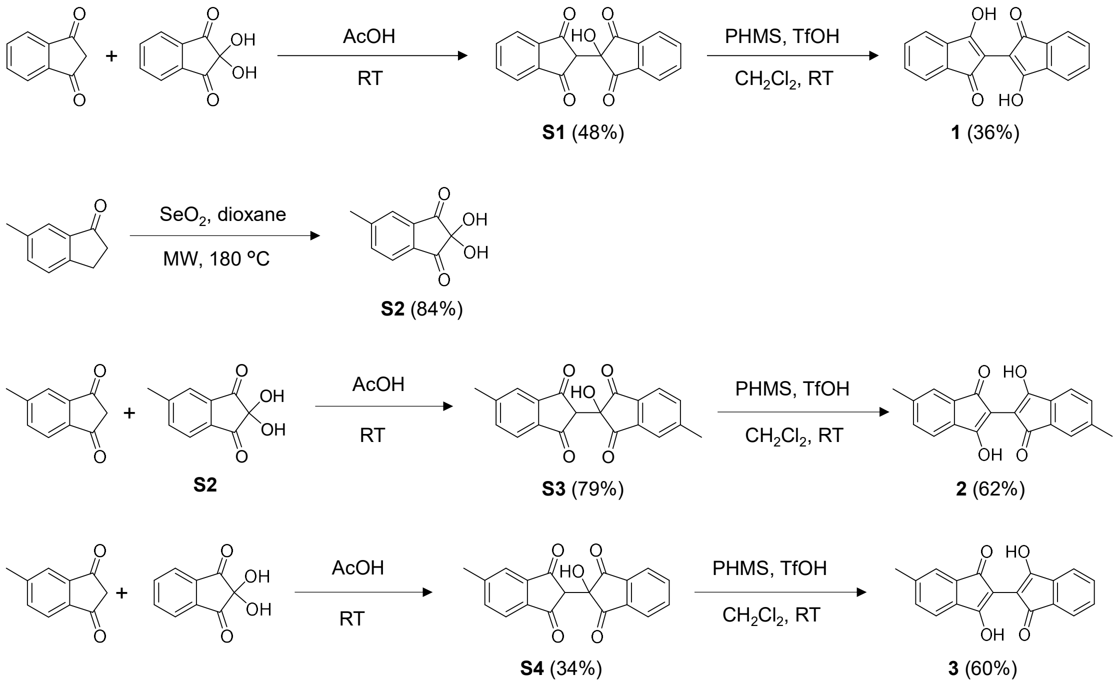

Figure S1 Synthetic scheme for compounds **1–3**.

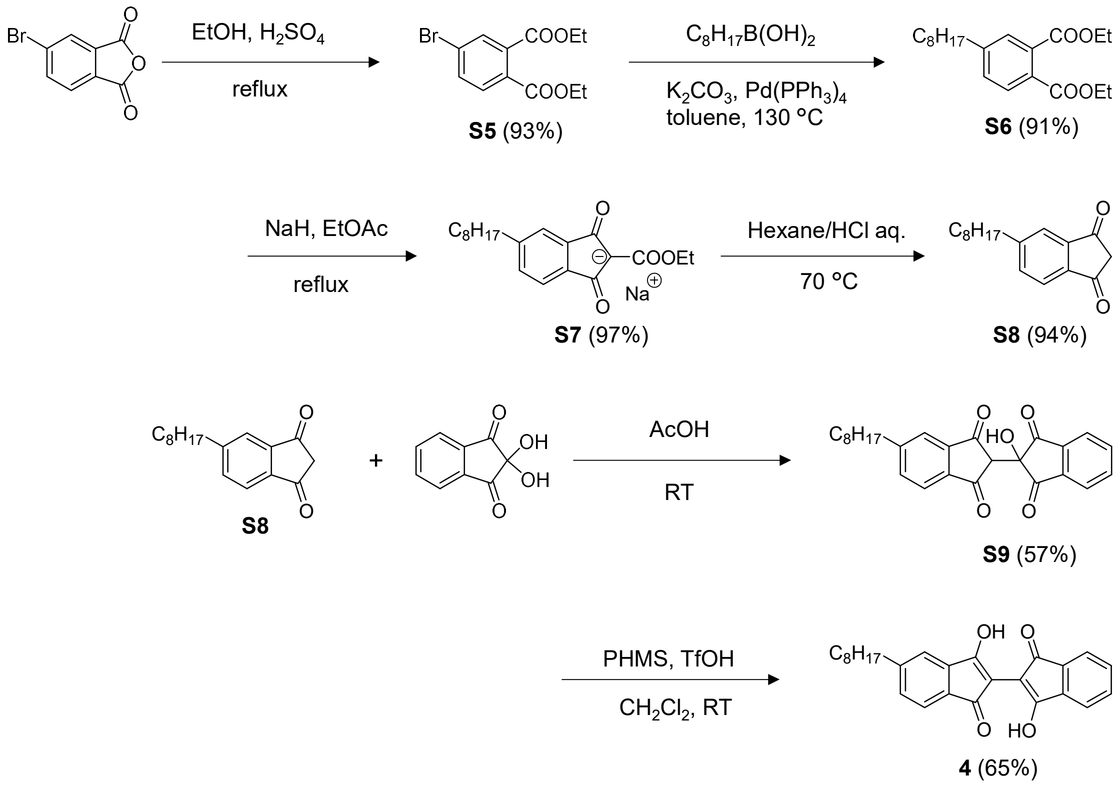

Figure S2 Synthetic scheme for compound **4**.

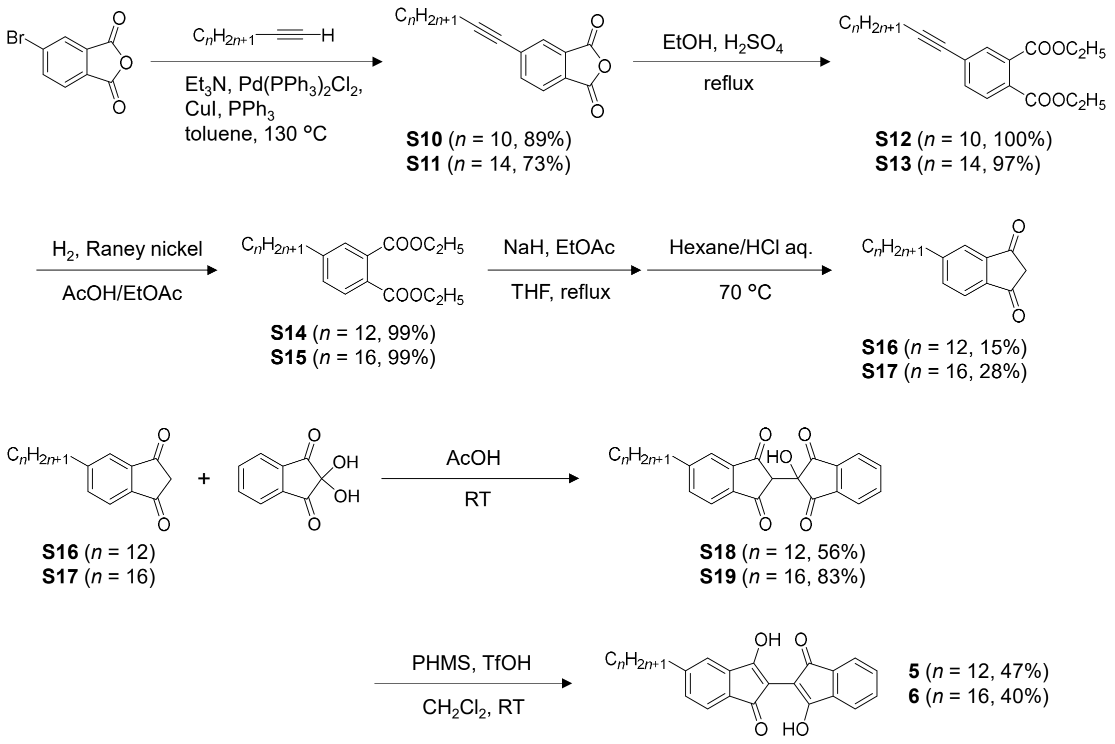

Figure S3 Synthetic scheme for compounds **5** and **6**.

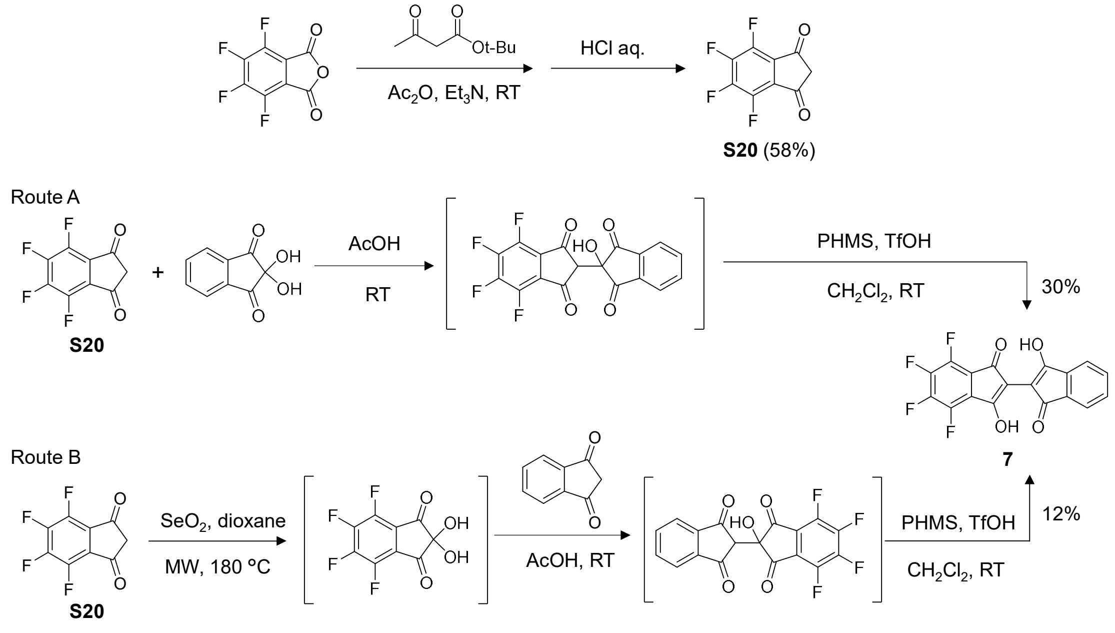

Figure S4 Synthetic scheme for compound **7**.

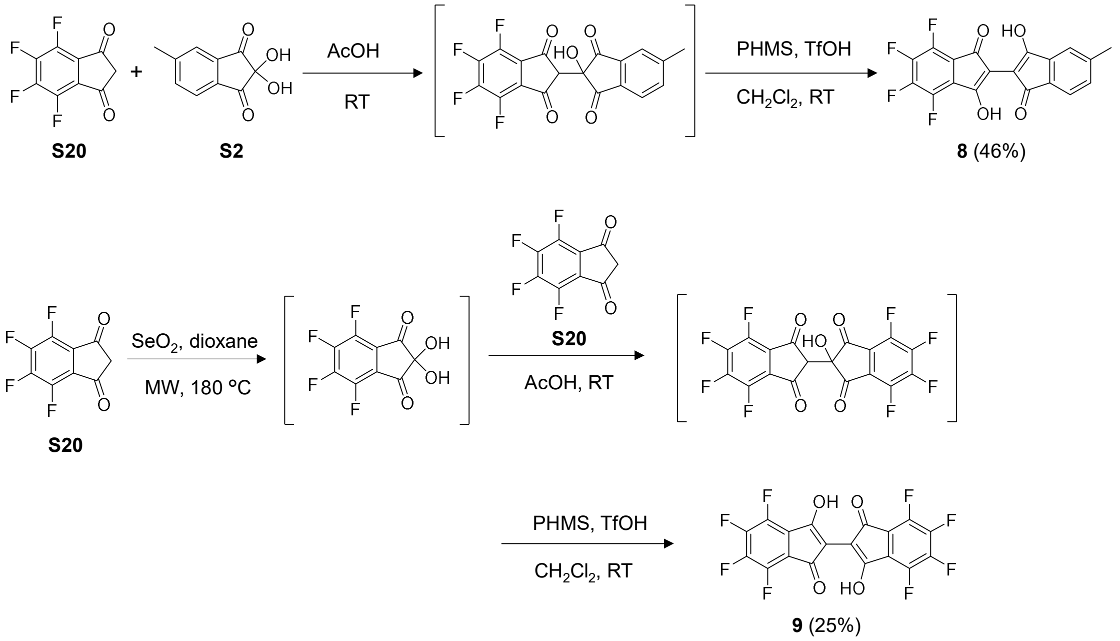

Figure S5 Synthetic scheme for compounds **8** and **9**.

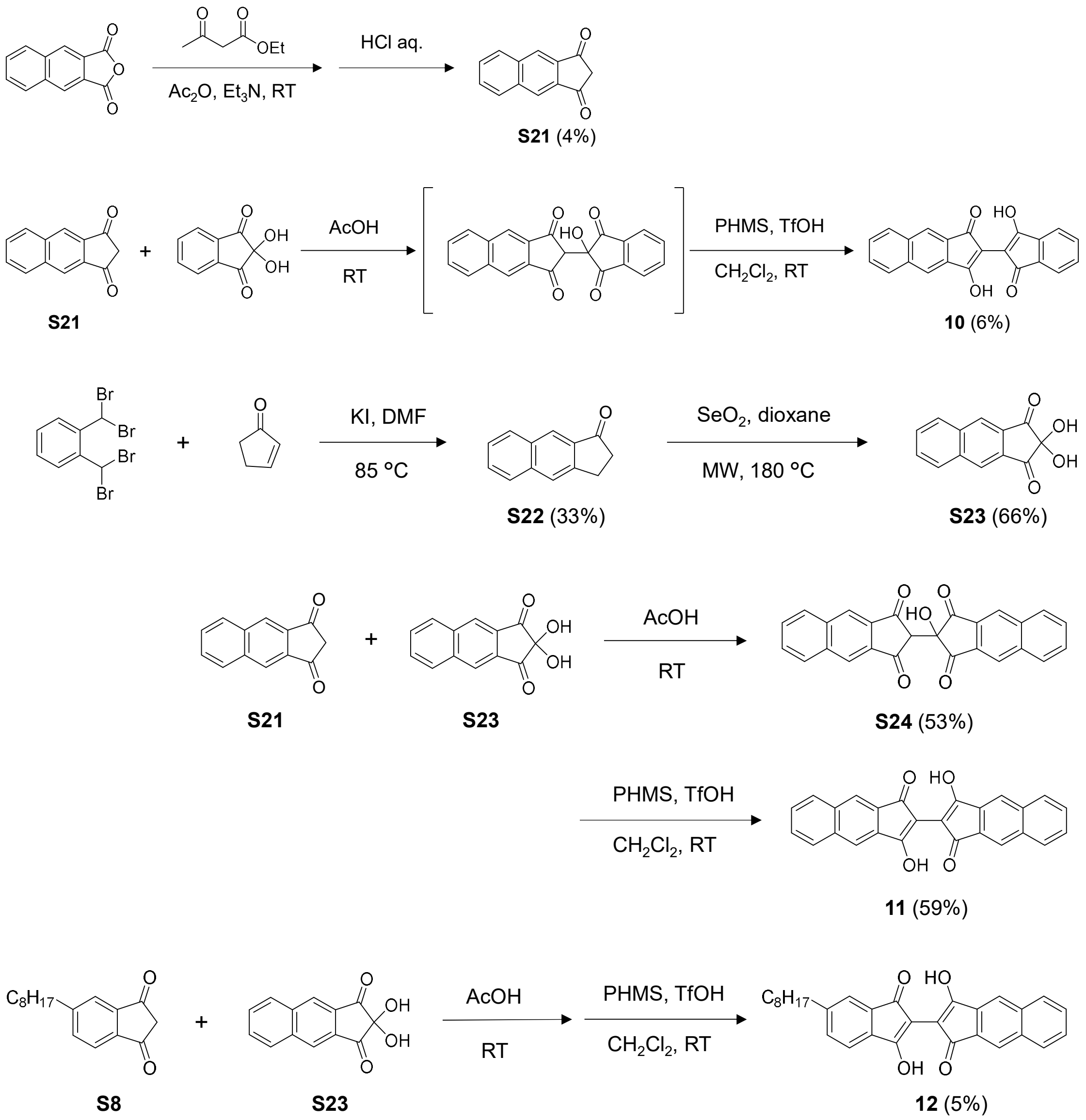

Figure S6 Synthetic scheme for compounds **10–12**.

Synthesis

2-Hydroxy-2,2′-biindan-1,1′,3,3′-tetraone (S1)[4]

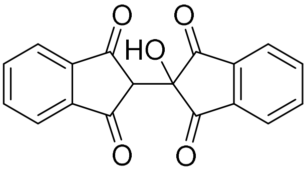

Ninhydrin (3.0 g, 16.8 mmol) and 1,3-indandione (2.2 g, 15.1 mmol) were dissolved in AcOH (120 mL) and stirred at room temperature overnight, resulting in the precipitation of a white solid. The solid was filtered off, washed with AcOH and water, and dried in vacuo to give the product as a white powder (2.2 g, 48%). ^{1}H NMR ($CDCl_3$, 399 MHz): δ 7.87–8.03 (m, 8H), 5.46 (s, 1H), 3.98 (s, 1H). ^{13}C{^{1}H} NMR ($CDCl_3$, 100 MHz): δ 197.41, 196.07, 142.09, 141.08, 136.74, 136.40, 124.40, 123.70, 76.15, 53.26. HRMS (FD/TOF) *m*/*z*: $[M]^+$ Calcd for $C_{18}H_{10}O_5$ 306.05282; Found 306.05285.

3,3′-Dihydroxy-2,2′-biindan-1,1′-dione (1)

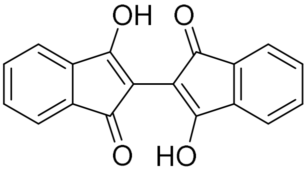

Trifluoromethanesulfonic acid (2 mL, 22.6 mmol) was added slowly to a solution of 2-hydroxy-2,2′-biindan-1,1′,3,3′-tetraone (**S1**, 0.61 g, 2 mmol) and poly(methylhydrosiloxane) (0.60 g, 10 mmol) in anhydrous $CHCl_3$ (100 mL) at room

temperature. The solution immediately turned deep purple. After the solution was stirred at room temperature for 30 min, it was extracted with NaOH aq. (1 M) three times. The combined aqueous phase was acidified with HCl aq. (1 M) to give a purple precipitate. The solid was collected by filtration and recrystallized from hot chlorobenzene to afford the product as needle-shaped purple crystals (0.21 g, 36%). ^{1}H NMR ($CDCl_3$, 399 MHz, 40 °C): δ 14.56 (s, 2H), 7.31 (m, 8H). ^{13}C{^{1}H} NMR ($CDCl_3$, 100 MHz, 40 °C): δ 187.59, 136.69, 132.17, 120.47, 103.50. HRMS (FD/TOF) *m*/*z*: $[M]^+$ Calcd for $C_{18}H_{10}O_4$ 290.05791; Found 290.05772. Anal. Calcd for $C_{18}H_{10}O_4$: C, 74.48; H, 3.47. Found: C, 74.22; H, 3.63. m.p. (DSC): 231 °C.

2,2-Dihydroxy-4-methylindan-1,3-dione (S2)[5]

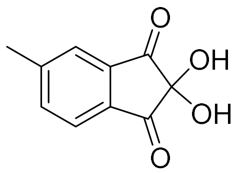

6-Methyl-1-indanone (1.46 g, 10 mmol), SeO_2 (3.44 g, 31 mmol), and dioxane (20 mL) were sealed in a glass tube and heated at 180 °C for 15 min with a microwave reactor. The resulting solution was evaporated in vacuo, and the residue was extracted with a minimum amount of hot water. The resulting clear solution was freeze-dried to afford the product as a white powder (1.62 g, 84%). ^{1}H NMR (DMSO-d_6, 399 MHz): δ 7.89 (dd, *J*

= 17.6, 8.0 Hz, 2H), 7.83 (s, 1H), 7.47 (s, 2H), 2.54 (s, 3H). $^{13}C\{^1H\}$ NMR ($CDCl_3$, 100 MHz): δ 197.72, 197.11, 149.18, 139.32, 138.71, 136.88, 124.30, 124.19, 88.39, 22.17. HRMS (FD/TOF) *m*/*z*: $[M - H_2O]^+$ Calcd for $C_{10}H_6O_3$ 174.03169; Found 174.03182.

2-Hydroxy-5,5′-dimethyl-2,2′-biindan-1,1′,3,3′-tetraone (S3)

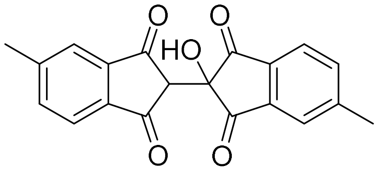

The product was synthesized from 2,2-dihydroxy-4-methylindan-1,3-dione (**S2**, 1.62 g, 8.4 mmol) and 5-methyl-1,3-indandione (1.34 g, 8.4 mmol) with a procedure similar to that for 2-hydroxy-2,2′-biindan-1,1′,3,3′-tetraone and the product was obtained as a white powder (2.22 g, 79%). ^1H NMR ($CDCl_3$, 399 MHz): δ 7.90 (d, *J* = 7.8 Hz, 1H), 7.80–7.85 (m, 2H), 7.65–7.73 (m, 3H), 5.47 (d, *J* = 1.8 Hz, 1H), 3.92 (s, 1H), 2.56 (s, 3H), 2.54 (s, 3H). $^{13}C\{^1H\}$ NMR ($CDCl_3$, 100 MHz): δ 197.79, 197.62, 197.10, 196.92, 196.34, 196.30, 195.81, 195.77, 148.59, 148.26, 142.55, 142.49, 141.49, 141.45, 140.04, 139.98, 139.00, 138.96, 137.91, 137.47, 124.28, 124.23, 123.67, 123.54, 53.53, 22.29, 22.20. HRMS (FD/TOF) *m*/*z*: $[M]^+$ Calcd for $C_{20}H_{14}O_5$ 334.08412; Found 334.08420.

5,5′-Dimethyl-2,2′-biindan-1,1′,3,3′-tetraone (2)

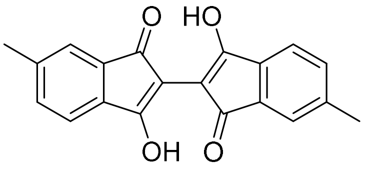

Trifluoromethanesulfonic acid (2 mL, 22.6 mmol) was added slowly to a solution of 2-hydroxy-5,5′-dimethyl-2,2′-biindan-1,1′,3,3′-tetraone (**S3**, 2.0 g, 6.0 mmol) and poly(methylhydrosiloxane) (3.6 g, 60 mmol) in anhydrous $CHCl_3$ (100 mL) at room temperature. The solution immediately turned green, and then deep purple. After the solution was stirred at room temperature for 1 h, it was extracted with NaOH aq. (1 M) three times. The combined aqueous phase was acidified with HCl aq. (1 M) to give a purple precipitate. The solid was collected by filtration and sublimed in vacuo to afford the product as needle-shaped purple crystals (1.18 g, 62%). The product was obtained as a mixture of conformational isomers (5,5′-dimethyl and 5,4′-dimethyl) in a 1:1 ratio. ^{1}H NMR ($CDCl_3$, 399 MHz, 40 °C): δ 14.69 (s, 0.5H), 14.59 (s, 1H), 14.53 (s, 0.5H), 7.20–7.16 (m, 2H), 7.05–7.10 (m, 4H), 2.35 (s, 6H). ^{13}C{^{1}H} NMR ($CDCl_3$, 100 MHz, 40 °C): δ 191.69, 187.84, 187.19, 183.12, 143.62, 142.94, 138.01, 137.03, 133.79, 132.81, 131.76, 131.24, 121.65, 121.37, 120.74, 120.42, 103.54, 103.47, 21.89, 21.83. HRMS (FD/TOF) *m*/*z*: $[M]^+$ Calcd for $C_{20}H_{14}O_5$ 318.08921; Found 334.08943. Anal. Calcd for $C_{20}H_{14}O_4$: C, 75.46; H, 4.43. Found: C, 75.40; H, 4.53. m.p. (DSC): 258 °C.

2-Hydroxy-5′-methyl-2,2′-biindan-1,1′,3,3′-tetraone (S4)

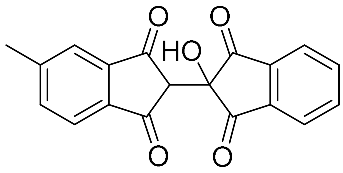

The product was synthesized from ninhydrin (0.89 g, 5.0 mmol) and 5-methyl-1,3-indandione (0.64 g, 4.4 mmol) with a procedure similar to that for 2-hydroxy-2,2′-biindan-1,1′,3,3′-tetraone and the product was obtained as a white powder (0.47 g, 34%). ^{1}H NMR ($CDCl_3$, 399 MHz): δ 7.99–8.04 (m, 2H), 7.89–7.93 (m, 2H), 7.84 (d, J = 7.8 Hz, 1H), 7.73 (m, 1H), 7.67 (d, J = 7.8 Hz, 1H), 5.51 (s, 1H), 3.95 (s, 1H), 2.55 (s, 3H). ^{13}C{^{1}H} NMR ($CDCl_3$, 100 MHz): δ 197.65, 196.96, 196.19, 196.17, 148.32, 142.50, 141.12, 141.08, 139.99, 137.53, 136.69, 124.36, 123.70, 123.57, 76.13, 53.48, 22.21. HRMS (FD/TOF) m/z: $[M]^+$ Calcd for $C_{19}H_{12}O_5$ 320.06847; Found 320.06850.

5-Methyl-2,2′-biindan-1,1′,3,3′-tetraone (3)

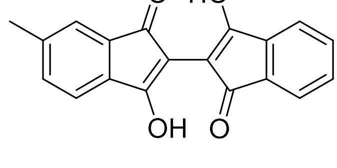

Trifluoromethanesulfonic acid (1 mL, 11.3 mmol) was added slowly to a solution of 2-hydroxy-5′-methyl-2,2′-biindan-1,1′,3,3′-tetraone (**S4**, 0.35 g, 1.1 mmol) and poly(methylhydrosiloxane) (0.63 g, 10.5 mmol) in anhydrous $CHCl_3$ (50 mL) at room

temperature. The solution immediately turned green, and then deep purple. After the solution was stirred at room temperature for 1 h, the solution was extracted with NaOH aq. (1 M) three times. The combined aqueous phase was acidified with HCl aq. (1 M) to give a purple precipitate. The solid was collected by filtration and dried to afford the product as a purple powder (0.19 g, 60%). ^{1}H NMR ($CDCl_3$, 399 MHz, 40 °C): δ 14.65 (s, 1H), 14.54 (s, 1H), 7.27–7.34 (m, 4H), 7.18 (d, J = 7.3 Hz, 1H), 7.09 (dd, J = 8.4, 7.8 Hz, 2H), 2.35 (s, 3H). ^{13}C{^{1}H} NMR ($CDCl_3$, 100 MHz): δ 189.88, 189.49, 185.43, 185.20, 143.41, 137.47, 137.10, 136.09, 135.79, 133.22, 132.34, 131.81, 131.57, 121.59, 120.67, 120.49, 120.19, 103.59, 103.32, 21.87. HRMS (FD/TOF) m/z: $[M]^+$ Calcd for $C_{19}H_{12}O_4$ 304.07356; Found 304.07380. Anal. Calcd for $C_{19}H_{12}O_4$: C, 74.99; H, 3.98. Found: C, 74.18; H, 4.09.

Diethyl 4-bromophthalate (S5)

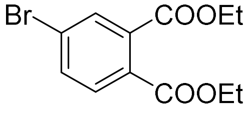

Concentrated H_2SO_4 (2 mL) was added to a solution of 4-bromophthalic anhydride (42.5 g, 0.187 mol) in EtOH (300 mL) in a 500 mL round-bottom flask equipped with a reflux condenser. After refluxing for 52 h, the reaction was quenched with $NaHCO_3$ aq. and EtOH was removed with a rotary evaporator. The solution was extracted with $CHCl_3$ and

the organic layer was dried over anhydrous $MgSO_4$. After removing the solvent with a rotary evaporator, the resulting clear liquid was used in the next step without further purification (52.6 g, 93%). ^{1}H NMR ($CDCl_3$, 399 MHz): δ 7.83 (d, *J* = 1.8 Hz, 1H), 7.61–7.69 (m, 2H), 4.33–4.42 (m, 4H), 1.37 (td, *J* = 7.2, 4.4 Hz, 6H).

Diethyl 4-octylphthalate (S6)

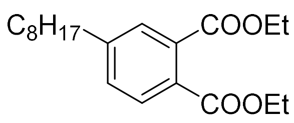

Diethyl 4-bromophthalate (**S5**, 20.8 g, 68.9 mmol), octylboronic acid (15.5 g, 98.0 mmol), and K_2CO_3 (28.5 g, 0.207 mol) were placed in a 300 mL three-necked round-bottom flask and flushed with N_2. Toluene (200 mL) and water (75 mL) were added to the flask and the mixture was bubbled with N_2 under stirring for 15 min. After adding $Pd(PPh_3)_4$ (2.4 g, 2.1 mmol), the mixture was refluxed for 29 h. The resulting mixture was extracted with $CHCl_3$/water and the organic layer was dried over anhydrous $MgSO_4$. After concentrating with a rotary evaporator, the resulting black liquid was subjected to column chromatography using hexane/EtOAc (9:1) as the eluent to afford the product as a clear liquid (21.0 g, 91%). ^{1}H NMR ($CDCl_3$, 399 MHz): δ 7.68 (d, *J* = 7.8 Hz, 1H), 7.47 (d, *J* = 1.8 Hz, 1H), 7.32 (dd, *J* = 8.0, 1.6 Hz, 1H), 4.30–4.41 (m, 4H), 2.65 (t, *J* = 7.8 Hz, 2H), 1.55–1.62 (m, 2H), 1.35–1.40 (m, 6H), 1.18–1.28 (m, 10H), 0.88 (t, *J* = 6.9 Hz, 3H).

Sodio-2-carboethoxy-5-octylindan-1,3-dione (S7)

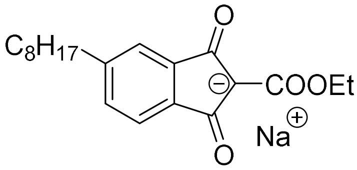

A solution of diethyl 4-octylphthalate (**S6**, 17.1 g, 51.1 mmol) in anhydrous EtOAc (10 mL, 0.10 mol) was added to a NaH dispersion in oil (60 wt %, 3.1 g, 76.7 mmol) in a 100 mL two-necked round-bottom flask equipped with a reflux condenser and a $CaCl_2$ tube. The mixture was refluxed for 6 h, and then diluted in EtOAc (100 mL) and poured into hexane (1 L). After stirring for a while, the precipitate was collected by filtration to give a yellow cake. The solid was dried in vacuum at 60 °C to give the product to use in the next step without further purification (16.4 g, 91%). ^{1}H NMR (DMSO-d_6, 399 MHz): δ 7.22 (s, 2H), 7.14 (s, 1H), 4.04 (q, J = 7.2 Hz, 2H), 2.61 (t, J = 7.5 Hz, 3H), 1.55–1.61 (m, 2H), 1.13–1.27 (m, 13H), 0.85 (t, J = 6.9 Hz, 3H). ^{13}C{^{1}H} NMR ($CDCl_3$, 100 MHz): δ 190.45, 190.31, 166.04, 146.21, 140.25, 137.62, 131.09, 119.39, 119.35, 97.36, 57.28, 35.86, 31.80, 31.46, 29.35, 29.18, 29.14, 22.61, 15.41, 14.48. HRMS (ESI/Q-TOF) m/z: $[M - Na]^-$ Calcd for $C_{20}H_{25}O_4$ 329.1753; Found 329.1751.

5-Octylindan-1,3-dione (S8)

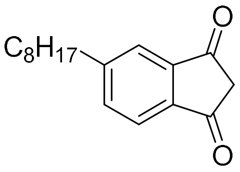

HCl aq. (200 mL, 1.2 M) was added to a suspension of finely ground sodio-2-carboethoxy-5-octylindan-1,3-dione (**S7**, 7.0 g, 19.8 mmol) in hexane (200 mL) in a 500 mL round-bottom flask equipped with a reflux condenser, and the mixture was heated at 70 °C under gentle hexane reflux for 3 h. After the powder disappeared and the hexane layer become a clear red solution, the hexane layer was separated and dried over anhydrous $MgSO_4$. Removing the solvent with a rotary evaporator afford the product as a red liquid that was used in the next step without further purification (4.79 g, 94%). ^{1}H NMR ($CDCl_3$, 399 MHz): δ 7.89 (d, *J* = 8.2 Hz, 1H), 7.77 (s, 1H), 7.65 (dd, *J* = 8.2, 1.4 Hz, 1H), 3.23 (s, 2H), 2.79 (t, *J* = 7.8 Hz, 2H), 1.62–1.71 (m, 2H), 1.26–1.31 (m, 10H), 0.88 (t, *J* = 6.9 Hz, 3H). HRMS (FD/TOF) *m*/*z*: $[M]^+$ Calcd for $C_{17}H_{22}O_2$ 258.16198; Found 258.16196.

2-Hydroxy-5′-octyl-2,2′-biindan-1,1′,3,3′-tetraone (S9)

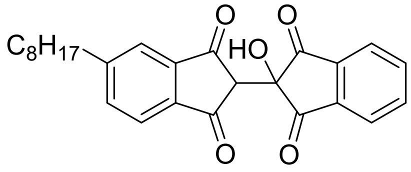

5-Octylindan-1,3-dione (**S8**, 2.58 g, 10 mmol) was added to a solution of ninhydrin (5.34 g, 30 mmol) in acetic acid (100 mL), and the mixture was stirred at room temperature for 43 h. The resulting brown dispersion was filtered, the filtrate was extracted with $CHCl_3$/water, and the organic layer was dried over anhydrous $MgSO_4$. After removing the solvent with a rotary evaporator, the resulting red liquid was subjected to column chromatography using hexane/EtOAc (9:1) as the eluent to afford the product as a pale-yellow liquid (2.4 g, 57%). ^{1}H NMR ($CDCl_3$, 399 MHz): δ 8.00–8.04 (m, 2H), 7.90–7.93 (m, 2H), 7.84 (d, J = 8.2 Hz, 1H), 7.73 (s, 1H), 7.67 (dd, J = 7.8, 1.4 Hz, 1H), 5.53 (s, 1H), 3.96 (s, 1H), 2.77 (t, J = 7.8 Hz, 2H), 1.62–1.69 (m, 2H), 1.24–1.31 (m, 10H), 0.88 (t, J = 6.9 Hz, 3H).

3,3′-Dihydroxy-5-octyl-2,2′-biindan-1,1′-dione (4)

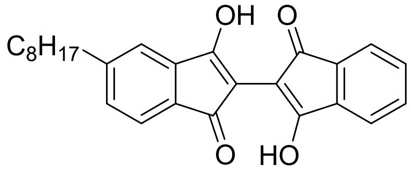

Trifluoromethanesulfonic acid (0.11 mL, 1.19 mmol) was added slowly to a solution of 2-hydroxy-5′-octyl-2,2′-biindan-1,1′,3,3′-tetraone (**S9**, 0.5 g, 1.19 mmol) and

poly(methylhydrosiloxane) (0.36 g, 5.95 mmol) in anhydrous CH_2Cl_2 (100 mL) at room temperature. The solution immediately turned deep purple. After the solution was stirred at room temperature for 30 min, the solution was extracted with NaOH aq. (1 M) three times. The combined aqueous phase was acidified with HCl aq. (1 M) to give a purple precipitate. The solid was collected by filtration and recrystallized from hot hexane to afford the product as purple needle-shaped crystals. (0.31 g, 65%). ^{1}H NMR ($CDCl_3$, 399 MHz): δ 14.66 (s, 1H), 14.57 (s, 1H), 7.27–7.34 (m, 4H), 7.20 (d, J = 7.3 Hz, 1H), 7.13 (s, 1H), 7.08 (d, J = 7.3 Hz, 1H), 2.59 (t, J = 7.8 Hz, 2H), 1.59–1.64 (m, 2H), 1.27–1.30 (m, 10H), 0.88 (t, J = 6.9 Hz, 3H). ^{13}C{^{1}H} NMR (100 MHz, $CDCl_3$): δ 189.62, 189.20, 185.68, 185.52, 148.51, 137.39, 137.03, 136.15, 133.46, 132.30, 131.83, 131.12, 120.92, 120.66, 120.45, 120.20, 103.61, 103.32, 36.21, 31.86, 30.99, 29.42, 29.22, 29.20, 22.67, 14.12. HRMS (FD/TOF) m/z: $[M]^+$ Calcd for $C_{26}H_{26}O_4$ 402.18311; Found 402.18291. Anal. Calcd for $C_{26}H_{26}O_4$: C, 77.59; H, 6.51. Found: C, 77.59; H, 6.57. m.p. (DSC): 146 °C.

4-(Dodec-1-yn-1-yl)phthalic anhydride (S10)

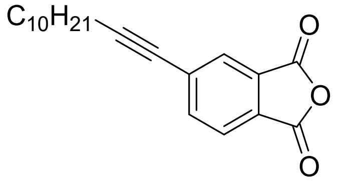

4-Bromophthalic anhydride (11.4 g, 50.0 mmol) and triethylamine (5.56 g, 55 mmol) were dissolved in toluene (60 mL) in a 100 mL two-necked round-bottom flask, and $Pd(PPh_3)_2Cl_2$ (0.21 g, 0.30 mmol), CuI (0.114 g, 0.6 mmol), and PPh_3 (0.236 g, 0.90 mmol) were added under N_2 and heated at 50 °C. 1-Dodecyne (19.5 g, 117 mmol) was slowly added and the solution was stirred at 50 °C for 18 h. The solvent was removed with a rotary evaporator and the residual black liquid was subjected to column chromatography using hexane/$CHCl_3$ as the eluent to afford the product as a yellow liquid (13.8 g, 89%). ^{1}H NMR ($CDCl_3$, 399 MHz): δ 7.96 (s, 1H), 7.91–7.93 (m, 1H), 7.84 (dd, J = 8.0, 1.1 Hz, 1H), 2.47 (t, J = 7.3 Hz, 2H), 1.60–1.67 (m, 2H), 1.42–1.49 (m, 2H), 1.27–1.31 (m, 12H), 0.88 (t, J = 6.9 Hz, 3H).

4-(Hexadec-1-yn-1-yl)phthalic anhydride (S11)

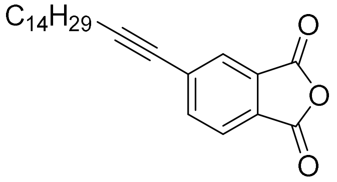

4-Bromophthalic anhydride (6.81 g, 30.0 mmol) and triethylamine (3.34 g, 3.3 mmol) were dissolved in toluene (36 mL) in a 100 mL two-necked round-bottom flask, and $Pd(PPh_3)_2Cl_2$ (0.126 g, 0.18 mmol), CuI (68.6 mg, 0.36 mmol), and PPh_3 (0.141 g, 0.54 mmol) were added under N_2 and heated at 50 °C. 1-Hexadecyne (8.0 g, 36 mmol) was

slowly added and the solution was stirred at 50 °C for 18 h. The solvent was removed with a rotary evaporator and the residual black liquid was subjected to column chromatography using hexane/$CHCl_3$ as the eluent to afford the product as a brown solid (8.1 g, 73%). ^{1}H NMR ($CDCl_3$, 399 MHz): δ 7.96 (s, 1H), 7.92 (d, *J* = 7.8 Hz, 1H), 7.84 (dd, *J* = 7.8, 1.4 Hz, 1H), 2.47 (t, *J* = 7.1 Hz, 2H), 1.60–1.67 (m, 2H), 1.42–1.49 (m, 2H), 1.22–1.31 (m, 20H), 0.88 (t, *J* = 6.9 Hz, 3H).

Diethyl 4-(dodec-1-yn-1-yl)phthalate (S12)

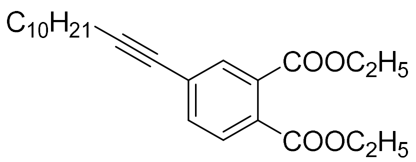

Concentrated H_2SO_4 (2 mL) was added to a solution of 4-(dodec-1-yn-1-yl)phthalic anhydride (**S10**, 13.8 g, 44 mmol) in EtOH (200 mL) in a 200 mL round-bottom flask equipped with a reflux condenser. After refluxing for 48 h, the reaction was quenched with $NaHCO_3$ aq. and EtOH was removed with a rotary evaporator. The solution was extracted with $CHCl_3$ and the organic layer was dried over anhydrous $MgSO_4$. After removing the solvent with a rotary evaporator, the resulting liquid was used in the next step without further purification (17.0 g, 100%). ^{1}H NMR ($CDCl_3$, 399 MHz): δ 7.66–7.69 (m, 2H), 7.50 (dd, *J* = 8.0, 1.6 Hz, 1H), 4.32–4.39 (m, 4H), 2.41 (t, *J* = 7.1 Hz, 2H),

1.57–1.64 (m, 2H), 1.40–1.46 (m, 2H), 1.33–1.40 (m, 6H), 1.20–1.33 (m, 12H), 0.88 (t, J = 6.6 Hz, 3H).

Diethyl 4-(hexadec-1-yn-1-yl)phthalate (S13)

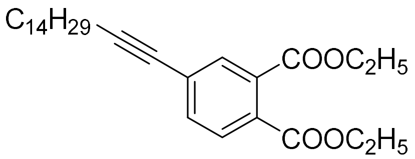

Concentrated H_2SO_4 (2 mL) was added to a solution of 4-(hexadec-1-yn-1-yl)phthalic anhydride (**S11**, 8.1 g, 22 mmol) in EtOH (200 mL) in a 200 mL round-bottom flask equipped with a reflux condenser. After refluxing for 48 h, the reaction was quenched with $NaHCO_3$ aq. and EtOH was removed with a rotary evaporator. The solution was extracted with $CHCl_3$ and the organic layer was dried over anhydrous $MgSO_4$. After removing the solvent with a rotary evaporator, the resulting liquid was used in the next step without further purification (9.4 g, 97%). ^{1}H NMR ($CDCl_3$, 399 MHz): δ 7.66–7.69 (m, 2H), 7.50 (dd, J = 8.1, 1.5 Hz, 1H), 4.32–4.40 (m, 4H), 2.41 (t, J = 7.0 Hz, 2H), 1.52–1.65 (m, 2H), 1.40–1.46 (m, 2H), 1.33–1.40 (m, 6H), 1.20–1.33 (m, 20H), 0.88 (t, J = 6.6 Hz, 3H).

Diethyl 4-dodecylphthalate (S14)

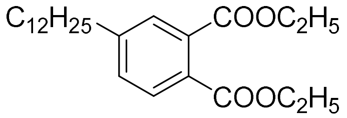

A solution of (diethyl 4-(dodec-1-yn-1-yl)phthalate (**S12**, 17.0 g, 44 mmol) in EtOAc (100 mL) was added to a dispersion of Pd/C (Pd 10%, 0.3 g) in EtOAc (100 mL) placed in a 500 mL three-necked round-bottom flask equipped with a N_2-filled balloon. The container was evacuated and filled with H_2 and the suspension was stirred at room temperature for 72 h. The solution was filtered through Celite 545 and the solvent was removed with a rotary evaporator to give the product as a yellow liquid (17.0 g, 99%). ^{1}H NMR ($CDCl_3$, 399 MHz): δ 7.68 (d, J = 8.2 Hz, 1H), 7.47 (d, J = 1.4 Hz, 1H), 7.32 (dd, J = 8.0, 1.6 Hz, 1H), 4.32–4.39 (m, 4H), 2.65 (t, J = 7.8 Hz, 2H), 1.58–1.65 (m, 2H), 1.33–1.41 (m, 6H), 1.18–1.31 (m, 18H), 0.88 (t, J = 6.9 Hz, 3H).

Diethyl 4-hexadecylphthalate (S15)

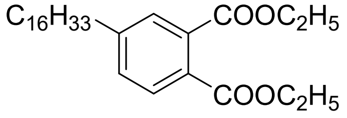

Diethyl 4-(hexadec-1-yn-1-yl)phthalate (**S13**, 9.44 g, 21.3 mmol), MeOH (5 mL), EtOAc (150 mL), and slurry of Raney nickel (8 mL) were placed in a 500 mL three-necked round-bottom flask equipped with a balloon. The container was evacuated and filled with H_2 and the suspension was stirred at room temperature for 48 h. The solution was filtered

through Celite 545 and the solvent was removed with a rotary evaporator to give the product as a brown liquid (9.42 g, 99%). ^{1}H NMR ($CDCl_3$, 399 MHz): δ 7.68 (d, J = 8.2 Hz, 1H), 7.47 (d, J = 1.8 Hz, 1H), 7.32 (dd, J = 8.2, 1.8 Hz, 1H), 4.32–4.39 (m, 4H), 2.65 (t, J = 7.8 Hz, 2H), 1.60 (t, J = 7.5 Hz, 2H), 1.33–1.43 (m, 6H), 1.20–1.32 (m, 26H), 0.88 (t, J = 6.9 Hz, 3H).

5-Dodecylindan-1,3-dione (S16)

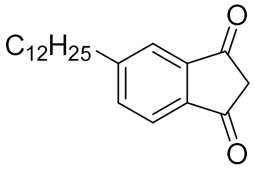

A solution of diethyl-4-dodecylphthalate (**S14**, 17.2 g, 44 mmol) in anhydrous EtOAc (10 mL, 0.10 mol) was added to a NaH dispersion in oil (60 wt %, 3.1 g, 76.7 mmol) in a 100 mL two-necked round-bottom flask equipped with a reflux condenser and a $CaCl_2$ tube. The mixture was refluxed for 6 h, and then diluted in EtOAc (100 mL) and poured into hexane (500 mL). The solution was placed in a 1 L round-bottom flask equipped with a reflux condenser, 1.2 M HCl aq. (200 mL) was added, and the mixture was heated at 70 °C under gentle hexane reflux for 1 h. The hexane layer was separated and dried over anhydrous $MgSO_4$. The solvent was removed with a rotary evaporator and the residue was subjected to column chromatography using hexane/EtOAc (9:1) as the eluent. The

product was recrystallized in hexane in a refrigerator to afford the product as a yellow powder (2.0 g, 15%). ^{1}H NMR ($CDCl_3$, 399 MHz): δ 7.89 (d, *J* = 7.8 Hz, 1H), 7.77 (s, 1H), 7.65 (dd, *J* = 7.8, 1.4 Hz, 1H), 3.23 (s, 2H), 2.79 (t, *J* = 7.5 Hz, 2H), 1.63–1.71 (m, 2H), 1.21–1.31 (m, 18H), 0.88 (t, *J* = 6.9 Hz, 3H). ^{13}C{^{1}H} NMR ($CDCl_3$, 100 MHz): δ 197.88, 197.18, 152.26, 143.80, 141.46, 136.25, 123.13, 122.52, 45.37, 36.34, 31.92, 31.04, 29.63, 29.50, 29.40, 29.35, 29.16, 22.70, 14.14. HRMS (FD/TOF) *m*/*z*: $[M]^+$ Calcd for $C_{21}H_{28}O_2$ 314.22458; Found 314.22444.

5-Hexadecylindan-1,3-dione (S17)

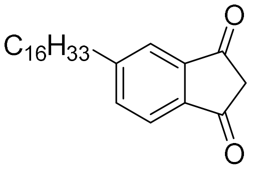

A solution of diethyl 4-hexadecylphthalate (**S15**, 9.5 g, 21 mmol) in anhydrous EtOAc (10 mL, 0.10 mol) was slowly added to an NaH dispersion in oil (60 wt %, 2.52 g, 63.0 mmol) placed in a 200 mL two-necked round-bottom flask equipped with a reflux condenser and a $CaCl_2$ tube. The mixture was refluxed for 24 h, and then diluted in EtOAc (100 mL) and quenched with water. The solution was extracted with dilute HCl aq./$CHCl_3$ and the organic layer was concentrated with a rotary evaporator. The remaining solid was dissolved in HCl aq. (10%)/hexane and the solution was stirred at 70 °C under gentle

hexane reflux for 2 h. The hexane layer was evaporated to give a solid, which was recrystallized from hexane to give the product as a green solid (2.2 g, 28%). ^{1}H NMR ($CDCl_3$, 399 MHz): δ 7.88 (d, *J* = 7.8 Hz, 1H), 7.77 (s, 1H), 7.64 (dd, *J* = 7.8, 1.4 Hz, 1H), 3.23 (s, 2H), 2.78 (t, *J* = 7.8 Hz, 2H), 1.63–1.71 (m, 2H), 1.22–1.31 (m, 26H), 0.88 (t, *J* = 6.9 Hz, 3H). ^{13}C{^{1}H} NMR ($CDCl_3$, 100 MHz): δ 197.89, 197.19, 152.31, 143.90, 141.46, 136.26, 123.14, 122.53, 45.37, 36.34, 31.93, 31.05, 29.70, 29.67, 29.63, 29.51, 29.40, 29.38, 29.16, 22.71, 14.14. HRMS (FD/TOF) *m*/*z*: $[M]^+$ Calcd for $C_{25}H_{38}O_2$ 370.28718; Found 370.28693.

2-Hydroxy-5′-dodecyl-2,2′-biindan-1,1′,3,3′-tetraone (S18)

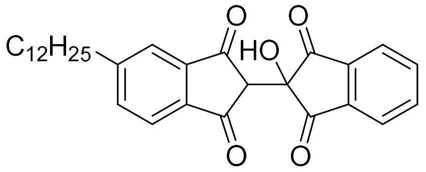

5-Dodecylindan-1,3-dione (**S16**, 2.0 g, 6.36 mmol) was added to a solution of ninhydrin (1.70 g, 9.5 mmol) in acetic acid (100 mL), and the mixture was stirred at room temperature for 48 h. The solution was extracted with $CHCl_3$/water and the organic layer was dried over anhydrous $MgSO_4$. After removing the solvent with a rotary evaporator, the resulting liquid was subjected to column chromatography using hexane/EtOAc (9:1) as the eluent to afford the product as a pale-green liquid (1.7 g, 56%). ^{1}H NMR ($CDCl_3$,

399 MHz): δ 8.01–8.03 (m, 2H), 7.90–7.92 (m, 2H), 7.84 (d, *J* = 7.8 Hz, 1H), 7.73 (s, 1H), 7.67 (dd, *J* = 7.8, 1.4 Hz, 1H), 5.53 (s, 1H), 3.96 (s, 1H), 2.77 (t, *J* = 7.5 Hz, 2H), 1.62–1.69 (m, 2H), 1.24–1.31 (m, 18H), 0.88 (t, *J* = 6.9 Hz, 3H).

2-Hydroxy-5′-hexadecyl-2,2′-biindan-1,1′,3,3′-tetraone (S19)

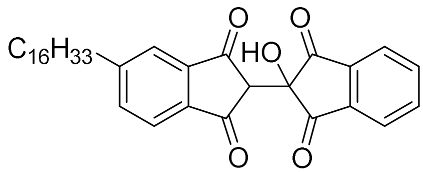

5-Hexadecylindan-1,3-dione (**S17**, 2.58 g, 10 mmol) was added to a solution of ninhydrin (1.44 g, 8.1 mmol) in acetic acid (100 mL) and $CHCl_3$ (10 mL), and the mixture was stirred at room temperature for 48 h. The resulting solution was extracted with $CHCl_3$/water and the organic layer was dried over anhydrous $MgSO_4$. After removing the solvent with a rotary evaporator, the resulting liquid was subjected to column chromatography using hexane/EtOAc (9:1) as the eluent to afford the product as a green oil (2.38 g, 83%). ^{1}H NMR ($CDCl_3$, 399 MHz): δ 8.01–8.03 (m, 2H), 7.90–7.92 (m, 2H), 7.84 (d, *J* = 7.8 Hz, 1H), 7.73 (s, 1H), 7.67 (dd, *J* = 7.8, 1.4 Hz, 1H), 5.53 (s, 1H), 3.96 (s, 1H), 2.77 (t, *J* = 7.8 Hz, 2H), 1.62–1.69 (m, 2H), 1.24–1.31 (m, 26H), 0.88 (t, *J* = 6.9 Hz, 3H).

3,3′-Dihydroxy-5-dodecyl-2,2′-biindan-1,1′-dione (5)

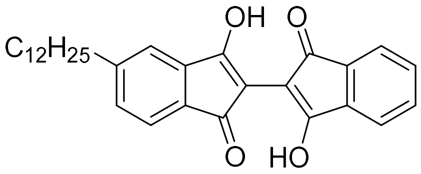

Trifluoromethanesulfonic acid (0.36 mL, 4.1 mmol) was added slowly to a solution of 2-hydroxy-5′-dodecyl-2,2′-biindan-1,1′,3,3′-tetraone (**S18**, 1.7 g, 3.6 mmol) and poly(methylhydrosiloxane) (1.22 g, 20 mmol) in anhydrous CH_2Cl_2 (100 mL) at room temperature. The solution immediately turned deep purple. The solution was stirred at room temperature for 40 min, and then was extracted with NaOH aq. (1 M) three times. The combined aqueous phase was acidified with HCl aq. (1 M) to give a purple precipitate. The solid was collected by filtration and recrystallized from hot hexane to afford the product as a purple powder (0.77 g, 47%). ^{1}H NMR ($CDCl_3$, 399 MHz): δ 14.66 (s, 1H), 14.57 (s, 1H), 7.27–7.34 (m, 4H), 7.20 (d, J = 7.3 Hz, 1H), 7.13 (s, 1H), 7.08 (dd, J = 7.3, 1.2 Hz, 1H), 2.59 (t, J = 7.6 Hz, 2H), 1.56–1.62 (m, 4H), 1.22–1.31 (m, 18H), 0.88 (t, J = 6.9 Hz, 3H). ^{13}C{^{1}H} NMR (100 MHz, $CDCl_3$): δ 189.55, 189.13, 185.84, 185.70, 148.55, 137.52, 137.17, 136.34, 133.66, 132.30, 131.86, 131.17, 120.98, 120.69, 120.49, 120.25, 103.70, 103.42, 36.26, 31.97, 30.98, 29.67, 29.58, 29.47, 29.38, 29.23, 22.71, 14.11. HRMS (FD/TOF) m/z: [M]$^+$ Calcd for $C_{30}H_{34}O_4$ 458.24571; Found 458.24566. Anal. Calcd for $C_{30}H_{34}O_4$: C, 78.57; H, 7.47. Found: C, 78.48; H, 7.56. m.p. (DSC):

134 °C.

3,3′-Dihydroxy-5-hexadecyl-2,2′-biindan-1,1′-dione (6)

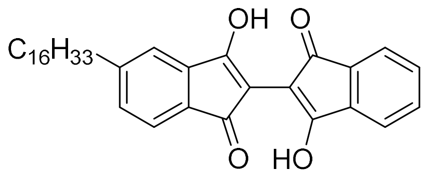

Trifluoromethanesulfonic acid (0.4 mL, 4.48 mmol) was added slowly to a solution of 2-hydroxy-5′-hexadecyl-2,2′-biindan-1,1′,3,3′-tetraone (**S19**, 2.38 g, 4.48 mmol) and poly(methylhydrosiloxane) (1.22 mL, 20 mmol) in anhydrous CH_2Cl_2 (100 mL) at room temperature. The solution immediately turned deep blue, and then deep purple, and some solidification was observed. After the solution was stirred at room temperature for 30 min, the solution was extracted with NaOH aq. (1 M) three times. The combined aqueous phase was acidified with HCl aq. (1 M) and extracted with $CHCl_3$. The organic layer was dried over anhydrous $MgSO_4$, the solvent was removed with a rotary evaporator, and the residual solid was subjected to column chromatography using hexane/$CHCl_3$ as the eluent. The product was recrystallized from hexane (1 L) to afford a purple powder (0.92 g, 40%). ^{1}H NMR ($CDCl_3$, 399 MHz): δ 14.61 (s, 1H), 14.52 (s, 1H), 7.28–7.34 (m, 4H), 7.19 (d, J = 7.3 Hz, 1H), 7.12 (s, 1H), 7.07 (d, J = 7.3 Hz, 1H), 2.59 (t, J = 7.7 Hz, 2H), 1.57–1.63 (m, 2H), 1.22–1.32 (m, 26H), 0.88 (t, J = 6.8 Hz, 3H). ^{13}C{^{1}H} NMR ($CDCl_3$, 100 MHz):

δ 189.65, 189.23, 185.70, 185.55, 148.53, 137.41, 137.00, 136.17, 133.44, 132.32, 131.85, 131.14, 120.94, 120.67, 120.47, 120.21, 103.61, 103.32, 36.21, 31.93, 30.98, 29.70, 29.66, 29.54, 29.43, 29.37, 29.17, 22.70, 14.14. HRMS (FD/TOF) *m*/*z*: $[M]^+$ Calcd for $C_{34}H_{42}O_4$ 514.30831; Found 514.30832. Anal. Calcd for $C_{34}H_{42}O_4$: C, 79.34; H, 8.23. Found: C, 78.85; H, 8.24. m.p. (DSC): 132 °C.

Tetrafluoroindan-1,3-dione (S20)

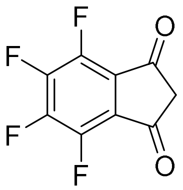

Triethylamine (3.84 g, 37.9 mmol) was added dropwise to a solution of tetrafluorophthalic anhydride (8.34 g, 37.9 mmol) and *tert*-butyl acetoacetate (8.99 g, 57 mmol) in acetic anhydride (50 mL). The solution was stirred overnight to form a yellow solid in red solution. The solid was filtered off and dispersed in HCl aq. (5 M) at room temperature for 1 h. The solid was filtered, dried in vacuo and further purified by sublimation to give the product as a yellow powder (4.76 g, 58%). ^{1}H NMR ($CDCl_3$, 399 MHz): δ 3.33 (s, 2H). ^{19}F{^{1}H} NMR ($CDCl_3$, 376 MHz): δ −135.15, −139.98. ^{13}C{^{1}H, ^{19}F} NMR ($CDCl_3$, 100 MHz): δ 190.45, 145.77, 142.97, 125.23, 45.59. HRMS (ESI/Q-TOF) *m*/*z*: $[M-H]^-$ Calcd for $C_9HO_2F_4$ 216.9913; Found 216.9910.

3,3′-Dihydroxy-4,5,6,7-tetrafluoro-2,2′-biindan-1,1′-dione (7)

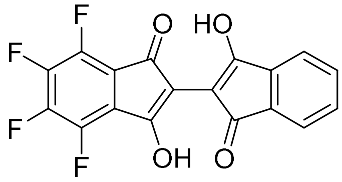

(A) Ninhydrin (89 mg, 0.5 mmol) and tetrafluoroindan-1,3-dione (**S20**, 0.11 g, 0.5 mmol) were dissolved in AcOH (10 mL) and stirred at room temperature overnight. The solution was freeze-dried and subjected to the next hydrogenation reaction without separating the intermediate. Trifluoromethanesulfonic acid (1 mL, 11.3 mmol) was added slowly to the solution of the powder and poly(methylhydrosiloxane) (0.60 g, 10 mmol) in dry $CHCl_3$ (10 mL) at room temperature. The solution immediately turned deep purple and a purple solid precipitated. The solid was filtered and recrystallized from chlorobenzene to afford the product as a purple solid (56 mg, 30%).

(B) Tetrafluoroindan-1,3-dione (**S20**, 0.10 g, 0.46 mmol), SeO_2 (76 mg, 0.69 mmol), dioxane (8 mL), and water (2 mL) were sealed in a glass tube and heated at 180 °C for 5 min in a microwave reactor. The resulting solution was evaporated in vacuo, and the residue was redissolved in $CHCl_3$ and dried over $MgSO_4$. The solution was filtered and the solvent was removed by rotary evaporator to give the crude tetrafluoroninhydrin product, which was used without purification. The product was synthesized with a

procedure similar to route (A) by the reactions between the crude tetrafluoroninhydrin product and indan-1,3-dione (20 mg, 12%). Routes (A) and (B) gave an identical product, as confirmed by ^{1}H and ^{19}F NMR. ^{1}H NMR ($CDCl_3$, 399 MHz): δ 14.48 (s, 2H), 7.35 (m, 4H). $^{19}F\{^{1}H\}$ NMR ($CDCl_3$, 376 MHz): δ −139.13, −146.76. $^{13}C\{^{1}H, ^{19}F\}$ NMR (TCE-d_2, 100 MHz, 120 °C): δ 187.52, 181.73, 142.07, 136.09, 132.37, 120.75, 116.87, 105.38, 102.25. HRMS (FD/TOF) *m*/*z*: $[M]^+$ Calcd for $C_{18}H_6F_4O_4$ 362.02022; Found 362.02020. Anal. Calcd for $C_{18}H_6F_4O_4$: C, 59.68; H, 1.67. Found: C, 59.27; H, 1.73.

3,3′-Dihydroxy-4,5,6,7-tetrafluoro-5-methyl-2,2′-biindan-1,1′-dione (8)

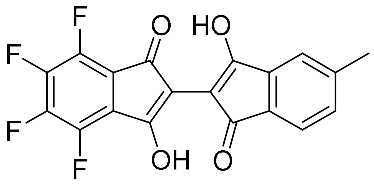

2,2-Dihydroxy-4-methylindan-1,3-dione (**S2**, 0.5 g, 2.6 mmol) and tetrafluoroindan-1,3-dione (0.57 g, 2.6 mmol) were dissolved in AcOH (20 mL) and stirred at room temperature overnight. The solution was freeze-dried and subjected to the next hydrogenation reaction without separating the intermediate. Trifluoromethanesulfonic acid (2 mL, 22.6 mmol) was added slowly to the solution of the powder and poly(methylhydrosiloxane) (1.0 g, 16.7 mmol) in dry CH_2Cl_2 (10 mL) at room temperature. The solution immediately turned deep purple and a purple solid precipitated.

The solid was filtered and dissolved in NaOH aq. (1 M) and filtered. The solution was acidified with HCl aq. (1 M) to give a purple precipitate. The solid was collected by filtration and recrystallized from hot chlorobenzene to afford the product as needle-shaped purple crystals (0.45 g, 46%). ^{1}H NMR ($CDCl_3$, 399 MHz): δ 14.63 (s, 1H), 14.33 (s, 1H), 7.12–7.22 (m, 3H), 2.37 (s, 3H). ^{19}F{^{1}H} NMR ($CDCl_3$, 376 MHz): δ −139.16, −139.52, −146.72, −147.38. ^{13}C{^{1}H, ^{19}F} NMR (TCE-*d*$_2$, 100 MHz, 120 °C): δ 189.23, 185.86, 182.93, 180.40, 143.87, 142.10, 136.93, 132.96, 131.96, 121.90, 120.97, 117.26, 116.57, 105.52, 102.09, 21.39. HRMS (FD/TOF) *m*/*z*: $[M]^+$ Calcd for $C_{19}H_8F_4O_4$ 376.03587; Found 376.03628. Anal. Calcd for $C_{19}H_8F_4O_4$: C, 60.65; H, 2.14. Found: C, 60.66; H, 2.26.

3,3′-Dihydroxy-4,4′,5,5′,6,6′,7,7′-octafluoro-2,2′-biindan-1,1′-dione (9)

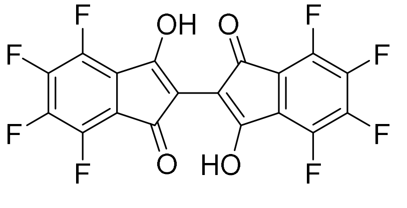

The product was synthesized with a procedure similar to that for 3,3′-dihydroxy-4,5,6,7-tetrafluoro-2,2′-biindan-1,1′-dione (**7**). Crude tetrafluoroninhydrin product generated from tetrafluoroindan-1,3-dione (0.1g, 0.46 mmol) was reacted with tetrafluoroindan-1,3-dione (**S20**, 0.1g, 0.46 mmol), and then hydrogenated with trifluoromethanesulfonic acid

(1 mL, 11.3 mmol) and poly(methylhydrosiloxane) (0.60 g, 10 mmol) in dry $CHCl_3$ (10 mL). The purple precipitate was recrystallized from hot chlorobenzene to give the product (50 mg, 25%). ^1H NMR (TCE-d_2, 399 MHz, 120 °C): δ 14.12 (s, 2H). $^{19}F\{^1H\}$ NMR (TCE-d_2, 399 MHz, 120 °C): δ −137.17, −145.54. $^{13}C\{^1H, ^{19}F\}$ NMR (TCE-d_2, 100 MHz, 120 °C): δ 182.04, 143.90, 142.40, 116.42, 104.21. HRMS (FD/TOF) *m*/*z*: $[M]^+$ Calcd for $C_{18}H_2F_8O_4$ 433.98253; Found 433.98175.

Benz[f]indan-1,3-dione (S21)

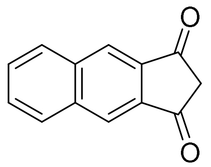

2,3-Naphthalenedicarboxylic anhydride (15.1 g, 76.2 mmol) was dissolved in a mixture of acetic anhydride (80 mL) and Et_3N (60 mL). Ethyl acetoacetate (29.7 g, 228 mmol) was added to the solution, and the mixture was stirred at room temperature for 24 h. The solution was poured into HCl aq. (600 mL, 2 M) and stirred overnight. The brown solid was filtered and dispersed in HCl aq. (300 mL, 5 M), and the suspension was refluxed for 4 h. After the suspension cooled, the solid was filtered and dried in vacuo. Repeated sublimations gave the product as a yellow powder (0.57 g, 4%). ^1H NMR ($CDCl_3$, 399 MHz): δ 8.51 (s, 2H), 8.10–8.14 (m, 2H), 7.71–7.76 (m, 2H), 3.38 (s, 2H). $^{13}C\{^1H\}$ NMR

($CDCl_3$, 100 MHz): δ 197.71, 138.16, 136.37, 130.66, 129.72, 124.32, 46.69. HRMS (ESI/Q-TOF) *m*/*z*: $[M + H]^+$ Calcd for $C_{13}H_9O_2$ 197.0603; Found 197.0602.

3,3′-Dihydroxy-2-benzo[f]indan-2′-indan-1,1′-dione (10)

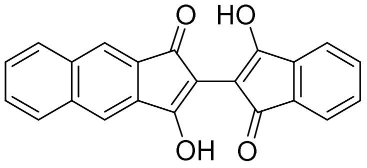

Ninhydrin (0.14 g, 0.76 mmol) and benz[f]indan-1,3-dione (**S21**, 0.1 g, 0.51 mmol) were dissolved in AcOH (10 mL) and stirred at room temperature overnight. The solution was freeze-dried and subjected to the next hydrogenation reaction without separating the intermediate. Trifluoromethanesulfonic acid (0.1 mL, 1.13 mmol) was added slowly to the solution of the powder and poly(methylhydrosiloxane) (1.0 g, 16.7 mmol) in dry $CHCl_3$ (10 mL) at room temperature. The solution immediately turned deep purple and a purple solid precipitated. The solid was filtered and dissolved in NaOH aq. (1 M) and filtered. The solution was acidified with HCl aq. (1 M) to give purple precipitate. The solid was collected by filtration and recrystallized from hot chlorobenzene to afford the product as needle-shaped purple crystals (11 mg, 6%). ^{1}H NMR (TCE-d_2, 399 MHz, 120 °C): δ 14.97 (s, 2H), 7.85 (q, J = 3.0 Hz, 2H), 7.74 (s, 2H), 7.55 (q, J = 3.1 Hz, 2H), 7.41 (s, 4H). $^{13}C\{^1H\}$ NMR (TCE-d_2, 100 MHz, 120 °C): δ 188.04, 186.10, 136.63,

135.21, 133.13, 132.04, 129.80, 128.22, 120.92, 120.36, 103.36. HRMS (FD/TOF) *m*/*z*: $[M]^+$ Calcd for $C_{22}H_{12}O_4$ 340.07356; Found 340.07369. Anal. Calcd for $C_{22}H_{12}O_4$: C, 77.64; H, 3.55. Found: C, 77.35; H, 3.67.

Benz[*f*]indan-1-one (S22)[6]

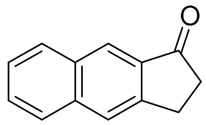

The compound was synthesized from 1,2-bis(dibromomethyl)benzene (25g, 59.3 mmol) and cyclopent-2-en-1-one (4.0 g, 48.7 mmol) by following the procedure reported by Wössner et al. and was obtained as a yellow solid (2.93 g, 33%). ^{1}H NMR ($CDCl_3$, 399 MHz): δ 8.34 (s, 1H), 8.00 (d, *J* = 8.2 Hz, 1H), 7.90 (s, 1H), 7.87 (d, *J* = 8.2 Hz, 1H), 7.57–7.61 (m, 1H), 7.48–7.52 (m, 1H), 3.34 (t, *J* = 6.2 Hz, 2H), 2.80–2.83 (m, 2H). $^{13}C\{^1H\}$ NMR ($CDCl_3$, 100 MHz): δ 207.49, 147.91, 137.17, 134.72, 132.31, 130.43, 128.57, 127.76, 126.12, 124.87, 124.42, 36.96, 25.35. HRMS (ESI/Q-TOF) *m*/*z*: $[M + H]^+$ Calcd for $C_{13}H_{11}O$ 183.0810; Found 183.0812.

Benzo[*f*]ninhydrin (S23)

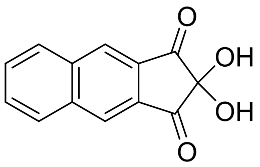

Benz[f]indan-1-one (**S21**, 0.18 g, 1 mmol), SeO_2 (0.343 g, 3.1 mmol), and dioxane (3 mL) were sealed in a glass tube and heated at 180 °C for 15 min in a microwave reactor. Silica gel was charged with the resulting reaction mixture and the mixture was subjected to silica column chromatography with a gradient eluent from hexane (100%) to hexane:EtOAc (50%) to afford the product as a brown powder (0.15 g, 66%). ^{1}H NMR (DMSO-d_6, 399 MHz): δ 8.74 (s, 2H), 8.36 (q, J = 3.2 Hz, 2H), 7.86 (q, J = 3.2 Hz, 2H), 7.55 (s, 2H). ^{13}C{^{1}H} NMR (DMSO-d_6, 100 MHz): δ 197.10, 136.47, 133.45, 130.63, 130.11, 125.34, 88.13. HRMS (FD/TOF) m/z: $[M - H_2O]^+$ Calcd for $C_{13}H_6O_3$ 210.03169; Found 210.03170.

2-Hydroxy-2,2′-bibenzo[*f*]indan-1,1′,3,3′-tetraone (S24)

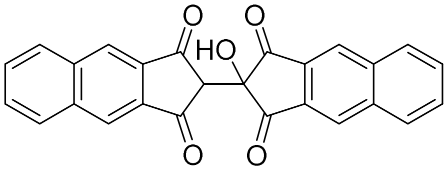

Benzo[f]ninhydrin (**S23**, 0.15 g, 0.65 mmol) and benz[f]indan-1,3-dione (**S21**, 0.13 g, 0.65 mmol) were dissolved in AcOH (20 mL) and stirred at room temperature overnight, resulting in the formation of an orange solid. The solid was filtered off, washed with

AcOH and water, and dried in vacuo to give the product as an orange powder (0.14 g, 53%). ^{1}H NMR ($CDCl_3$, 399 MHz): δ 7.87–8.03 (m, 8H), 5.46 (s, 1H), 3.98 (s, 1H). $^{13}C\{^1H\}$ NMR ($CDCl_3$, 100 MHz): δ 197.84, 196.56, 136.96, 136.64, 136.60, 130.82, 130.77, 130.23, 130.13, 125.93, 125.15, 77.22, 55.22. HRMS (FD/TOF) *m*/*z*: $[M]^+$ Calcd for $C_{26}H_{14}O_5$ 406.08412; Found 406.08448.

3,3′-Dihydroxy-2,2′-bibenzo[*f*]indan-1,1′-dione (11)

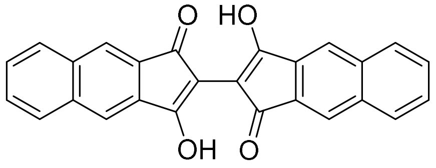

Trifluoromethanesulfonic acid (1 mL, 11.3 mmol) was added slowly to a solution of 2-hydroxy-2,2′-bibenzo[f]indan-1,1′,3,3′-tetraone (**S24**, 0.10 g, 0.25 mmol) and poly(methylhydrosiloxane) (0.30 g, 5 mmol) in anhydrous $CHCl_3$ (30 mL) at room temperature. The solution immediately turned deep purple and a purple solid precipitated. The solution was stirred at room temperature for 30 min, and then it was extracted with NaOH aq. (1 M) three times. The combined aqueous phase was acidified with HCl aq. (1 M) to give purple precipitate. The solid was collected by filtration and recrystallized from hot chlorobenzene to afford the product as needle-shaped purple crystals (58 mg, 59%). ^{1}H NMR (TCE-d_2, 399 MHz, 125 °C): δ 15.48 (s, 2H), 7.79–7.88 (m, 4H), 7.75–7.76 (m,

4H), 7.56–7.59 (m, 4H). HRMS (ESI/Q-TOF) *m*/*z*: [M − H]$^-$ Calcd for $C_{26}H_{13}O_4$ 389.0714; Found 389.0816. Anal. Calcd for $C_{26}H_{14}O_4$: C, 79.99; H, 3.61. Found: C, 80.09; H, 3.73. The solubility of **11** was too low for ^{13}C NMR measurement.

3,3′-Dihydroxy-5′-octyl-2-benzo[f]indan-2′-indan-1,1′-dione (12)

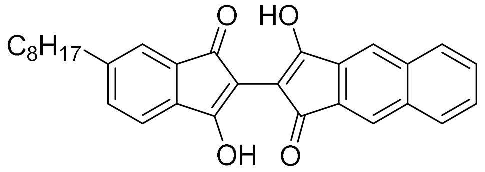

Benzo[f]ninhydrin (**S23**, 0.59 g, 2.6 mmol) and 5-octylindan-1,3-dione (**S8**, 0.67 g, 2.6 mmol) were dissolved in AcOH (20 mL) and stirred at room temperature overnight. The solution was freeze-dried and subjected to the next hydrogenation reaction without separating the intermediate. Trifluoromethanesulfonic acid (1 mL, 11.3 mmol) was added slowly to the solution of the powder and poly(methylhydrosiloxane) (1.0 g, 16.7 mmol) in dry $CHCl_3$ (10 mL) at room temperature. The solution immediately turned deep purple. The solution was extracted with NaOH aq. (1 M), and then with HCl aq. (1 M)/$CHCl_3$. The organic layer was dried over anhydrous $MgSO_4$, the solvent was removed with a rotary evaporator, and the residual solid was subjected to column chromatography using hexane/$CHCl_3$ as the eluent. The solid was collected by filtration and recrystallized from hexane to afford the product as needle-shaped purple crystals (34 mg, 5%). ^1H NMR

($CDCl_3$, 399 MHz): δ 15.19 (s, 1H), 15.18 (s, 1H), 7.78 (t, *J* = 4.6 Hz, 2H), 7.66 (d, *J* = 7.9 Hz, 2H), 7.49 (td, *J* = 3.5, 2.1 Hz, 2H), 7.22–7.25 (m, 1H), 7.17 (s, 1H), 7.12 (d, *J* = 7.3 Hz, 1H), 2.61 (t, *J* = 7.6 Hz, 2H), 1.27–1.32 (m, 12H), 0.88 (t, *J* = 6.9 Hz, 3H) $^{13}C\{^1H\}$ NMR ($CDCl_3$, 100 MHz): δ 190.18, 187.79, 186.79, 184.95, 148.66, 137.35, 135.28, 135.06, 133.57, 133.33, 132.99, 131.38, 130.10, 129.96, 128.43, 128.29, 121.25, 121.09, 120.88, 120.79, 109.26, 103.35, 36.24, 31.86, 31.01, 29.42, 29.21, 22.67, 14.12. HRMS (FD/TOF) *m*/*z*: $[M]^+$ Calcd for $C_{30}H_{28}O_4$ 452.19876; Found 452.19903. Anal. Calcd for $C_{30}H_{28}O_4$: C, 79.62; H, 6.24. Found: C, 78.65; H, 6.27. m.p. (DSC): 182 °C.

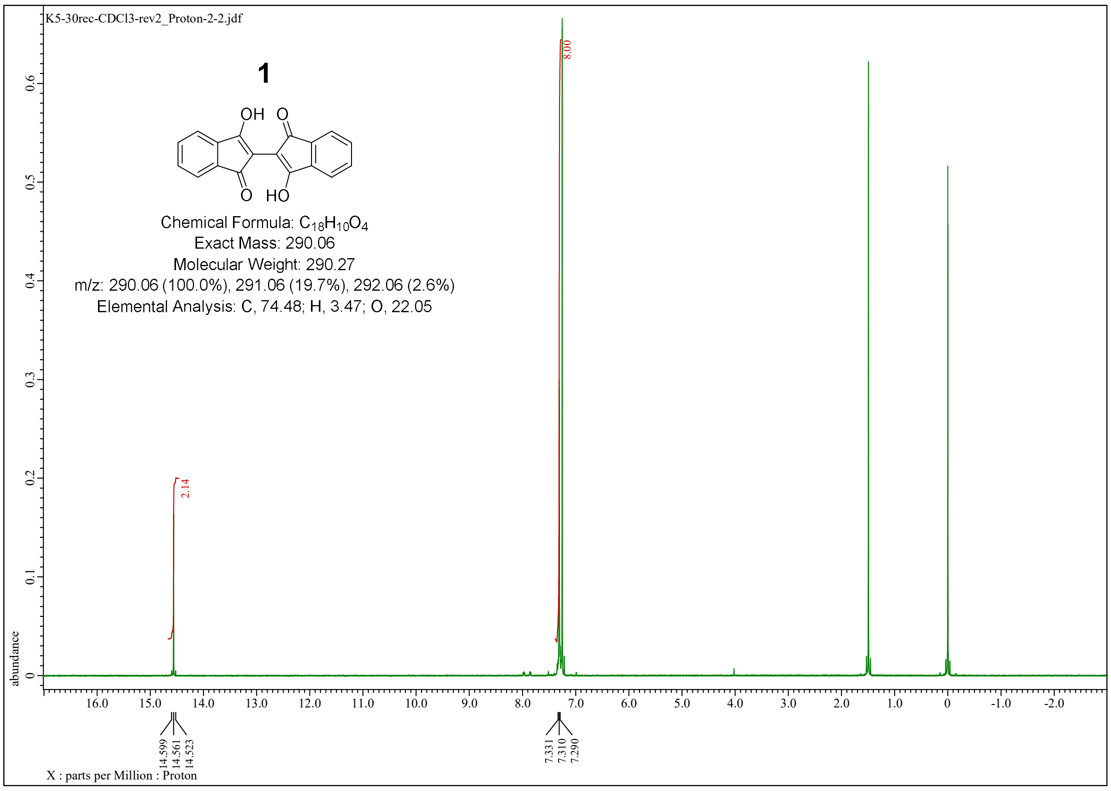

Figure S7 ^{1}H NMR spectrum of **1** in $CDCl_3$. All the peaks are assigned to the enol form.

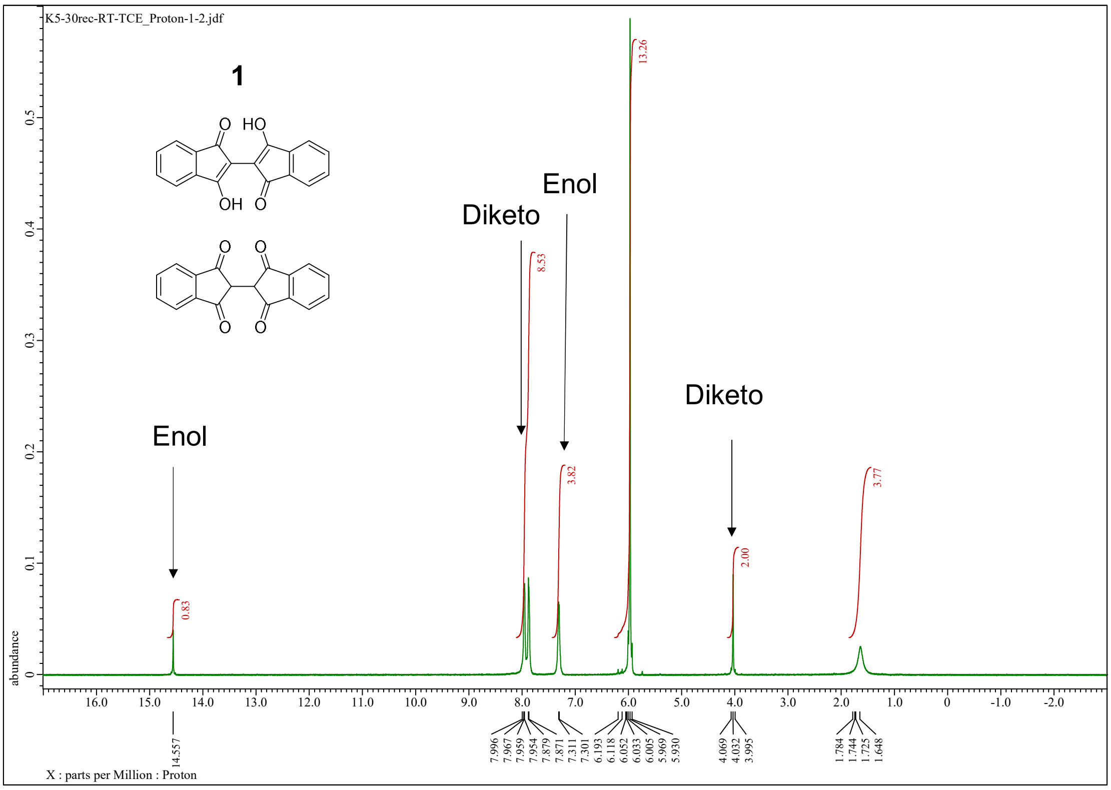

Figure S8 ^{1}H NMR spectrum of **1** in tetrachloroethane-d_2 at room temperature. Both the enol and the diketo forms were observed in a ratio of about 1:2.4.

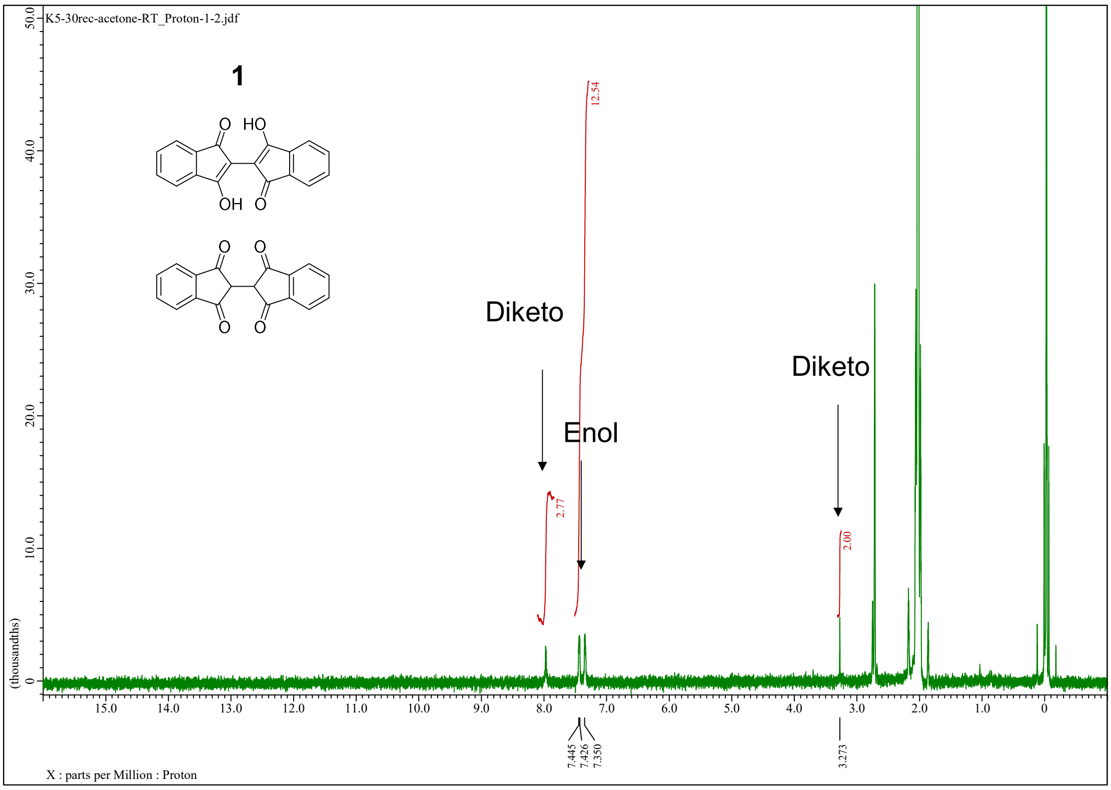

Figure S9 ^{1}H NMR spectrum of **1** in acetone-d_6 at room temperature. Both the enol and the diketo forms were observed in a ratio of about 1:0.22. The -OH peak in the enol form is absent, probably due to the exchange with water in the solvent.

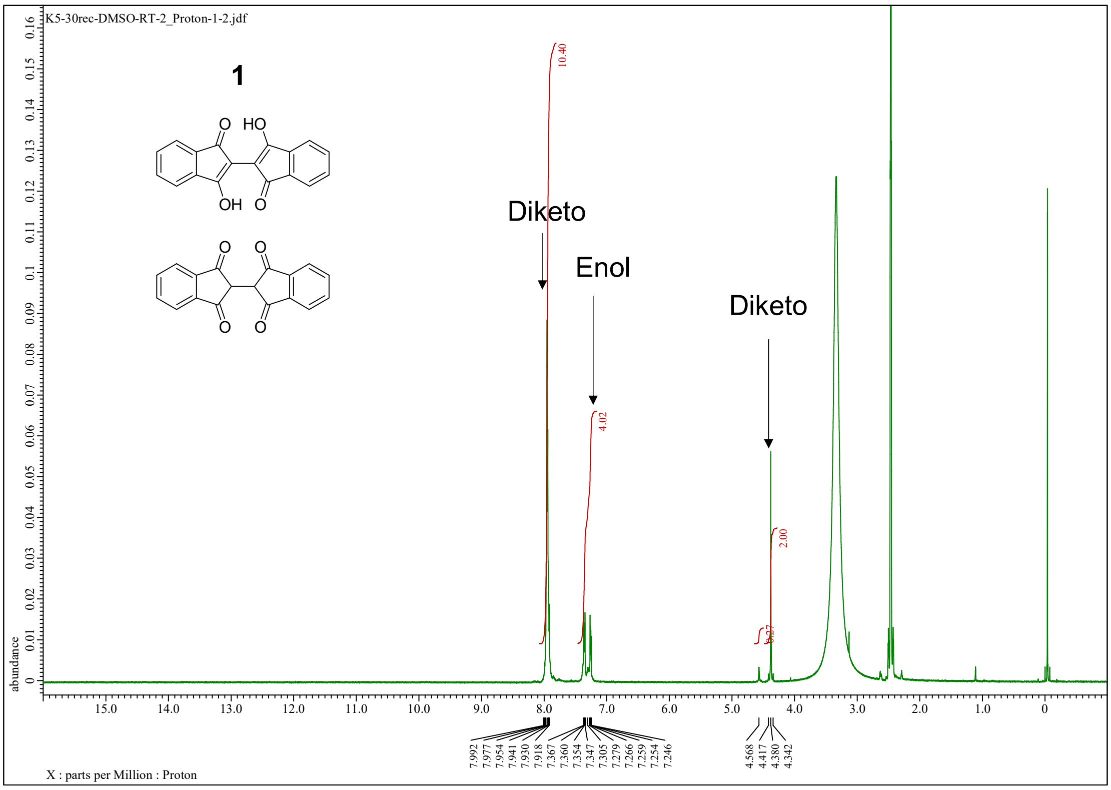

Figure S10 ^{1}H NMR spectrum of **1** in DMSO-d_6 at room temperature. Both the enol and the diketo forms were observed in a ratio of about 1:2.6. The -OH peak in the enol form is absent, probably due to the exchange with water in the solvent.

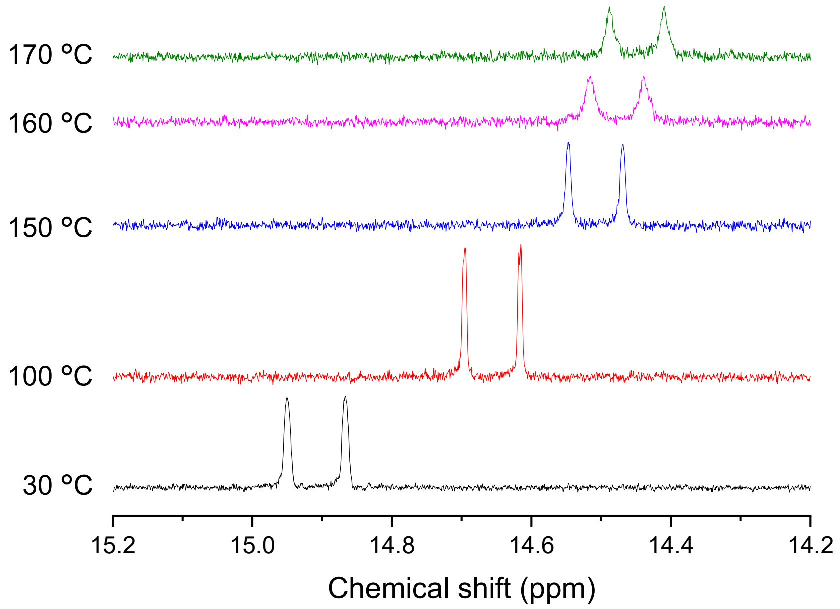

Figure S11 VT ^{1}H NMR spectra of **4** in the region of the enol -OH protons in *o*-dichlorobenzne-d_4. The peak separations are 0.079 ppm at 30 °C and 0.083 ppm at 170 °C, showing no sign of the coalescence of the peaks.

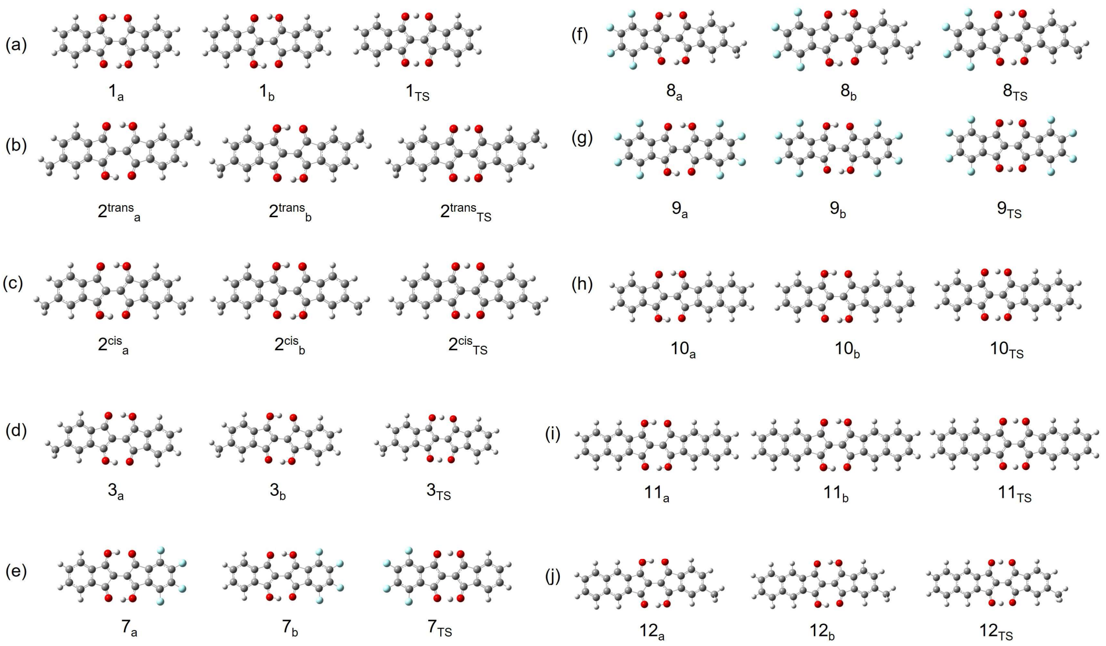

Figure S12 Optimized structures of the tautomers (defined as a and b) and the TS structure for the neutral states of (a) **1**, (b) **2^{cis}**, (c) **2^{trans}**, (d) **3**, (e) **7**, (f) **8**, (g) **9**, (h) **10**, (i) **11**, and (j) **12** (model).

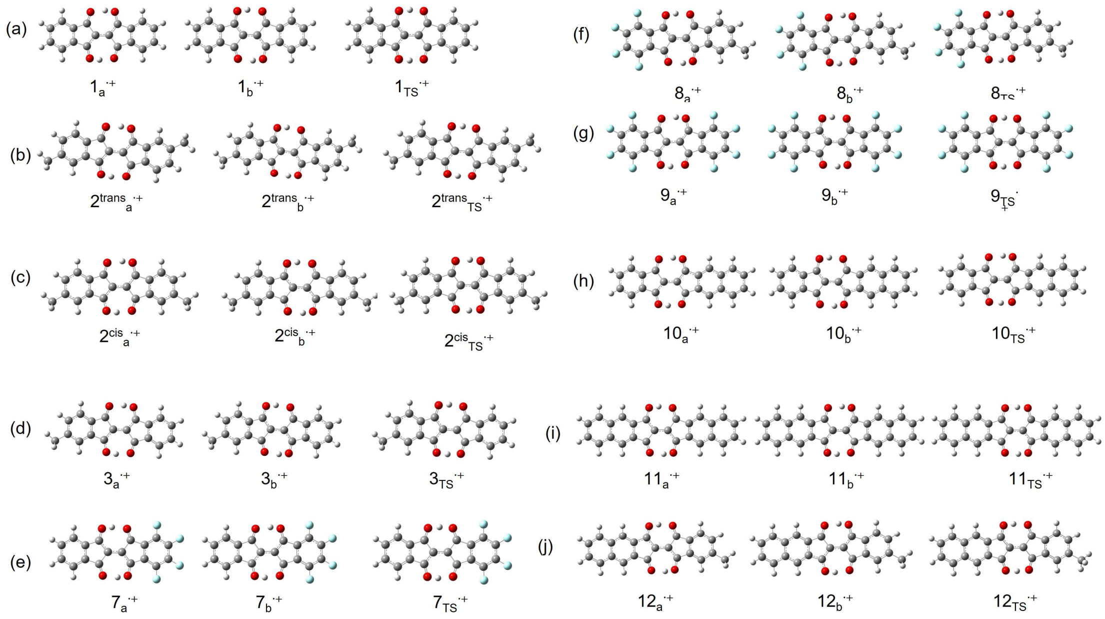

Figure S13 Optimized structures of the tautomers (defined as a and b) and the TS structure for the radical cations of (a) **1**, (b) **2^{cis}**, (c) **2^{trans}**, (d) **3**, (e) **7**, (f) **8**, (g) **9**, (h) **10**, (i) **11**, and (j) **12** (model).

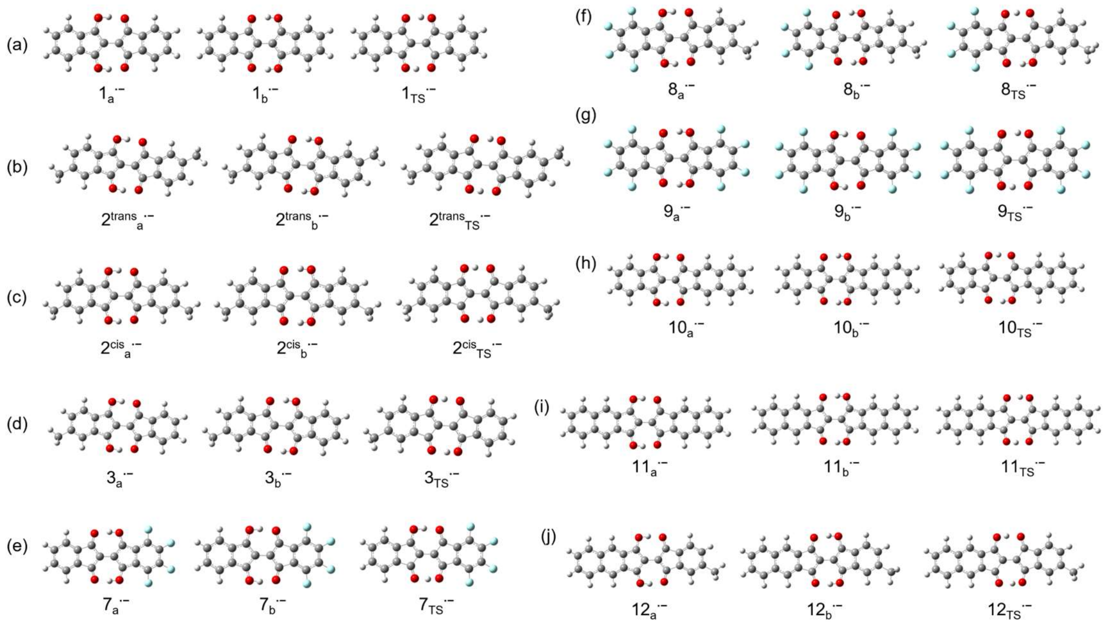

Figure S14 Optimized structures of the tautomers (defined as a and b) and the TS structure for the radical anions of (a) **1**, (b) $\mathbf{2^{cis}}$, (c) $\mathbf{2^{trans}}$, (d) **3**, (e) **7**, (f) **8**, (g) **9**, (h) **10**, (i) **11**, and (j) **12** (model).

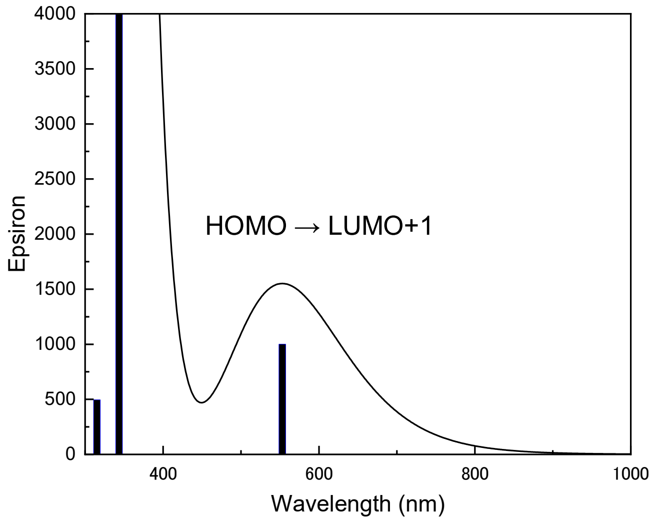

Figure S15 UV-vis absorption spectrum of **1** predicted by time-dependent DFT calculation. The HOMO → LUMO transition at 610 nm is prohibited by the orbital symmetry.

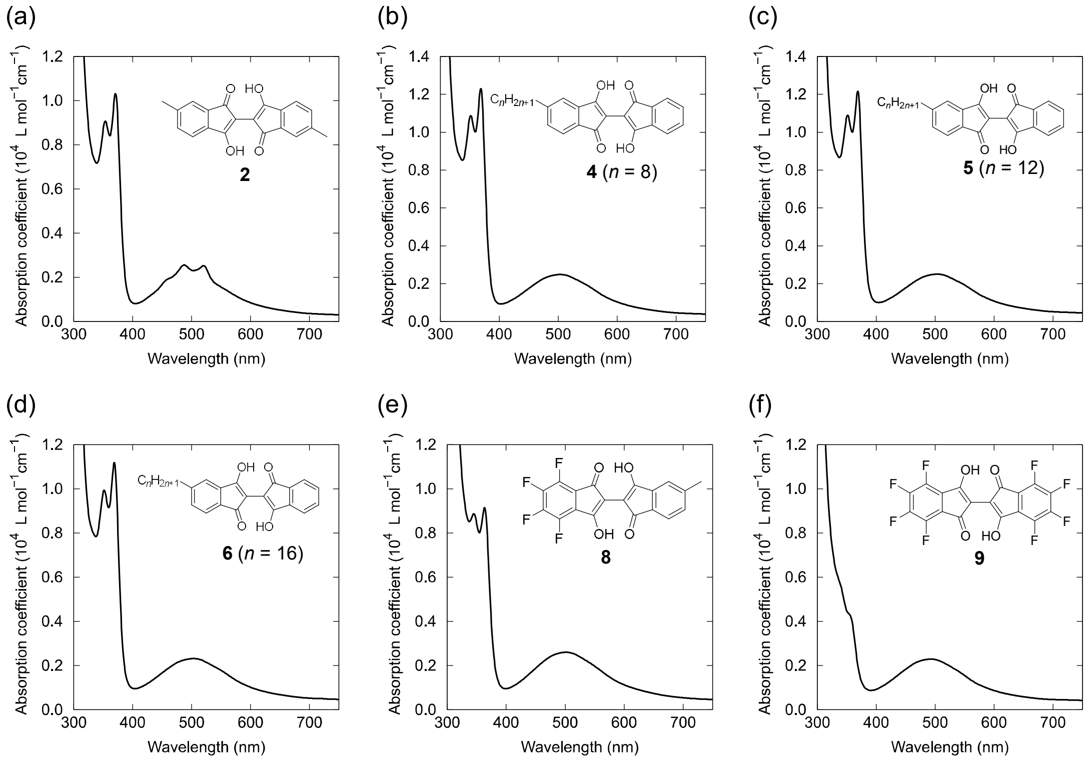

Figure S16 (a) Absorption spectra of (a) **2**, (b) **4**, (c) **5**, (d) **6**, (e) **8**, and (f) **9** in $CHCl_3$ solutions.

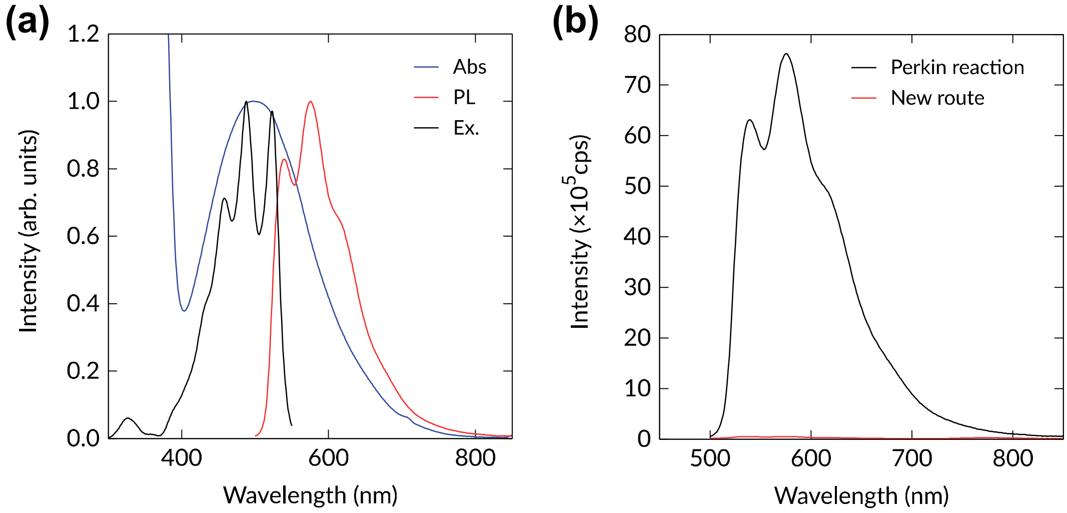

Figure S17 (a) UV-vis absorption, photoluminescence (λ_{ex} = 470 nm), and excitation spectra (λ_{em} = 600 nm) in $CHCl_3$ for **1** synthesized by the Perkin reaction (Figure 2a). The mismatch between the absorption and the excitation spectra indicates the presence of a fluorescent impurity. (b) Comparison of photoluminescence spectra of **1** synthesized by the Perkin reaction (Figure 2a) and the new route (Figure 2b) (λ_{ex} = 470 nm). Abs.: Absorbance; PL: Photoluminescence; Ex.: Excitation.

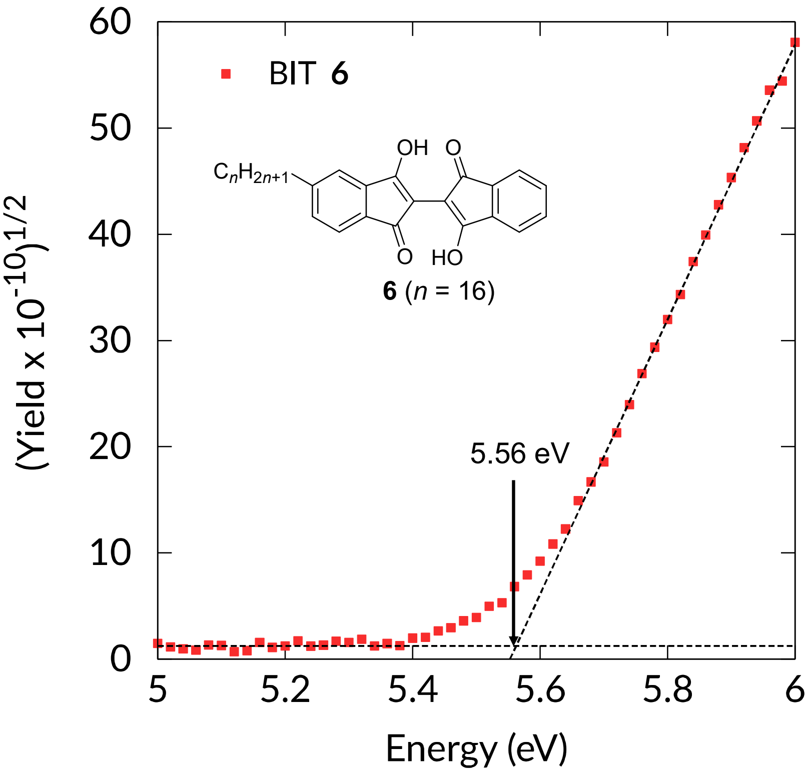

Figure S18 Photoemission yield spectroscopy in air performed on a film of **6** prepared by vacuum deposition. IE is estimated as 5.56 eV from the onset.

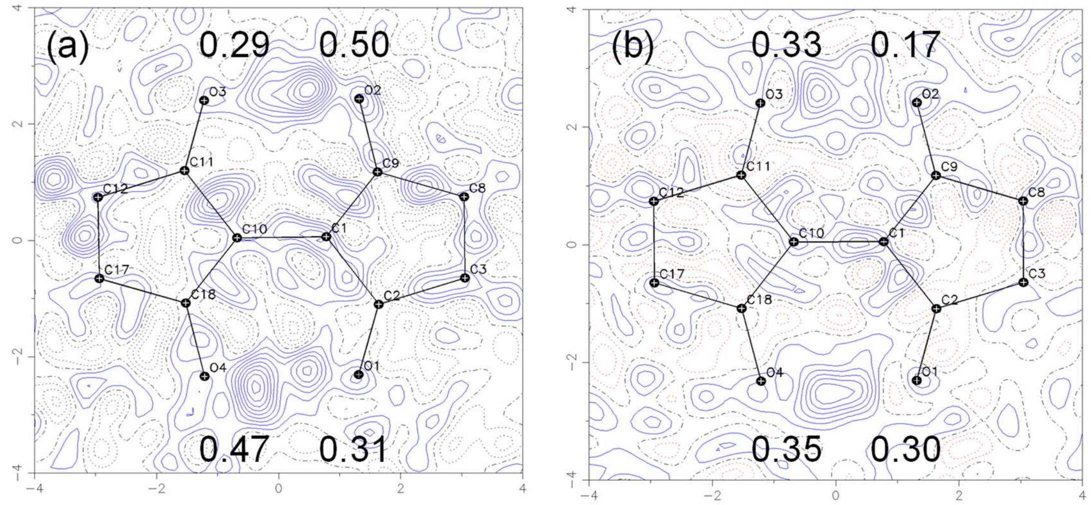

Figure S19 Contour plots of the difference Fourier maps for the molecular plane of the central part of **1** calculated using the structure model without the H atoms on the -OH groups and the measured diffractions at (a) 90 K and (b) 298 K. The contours were drawn at 0.05 e/Å^3 intervals. The numbers in the maps show the electron density maxima between the O…O groups in e/Å^3, indicating the position of the -OH protons.

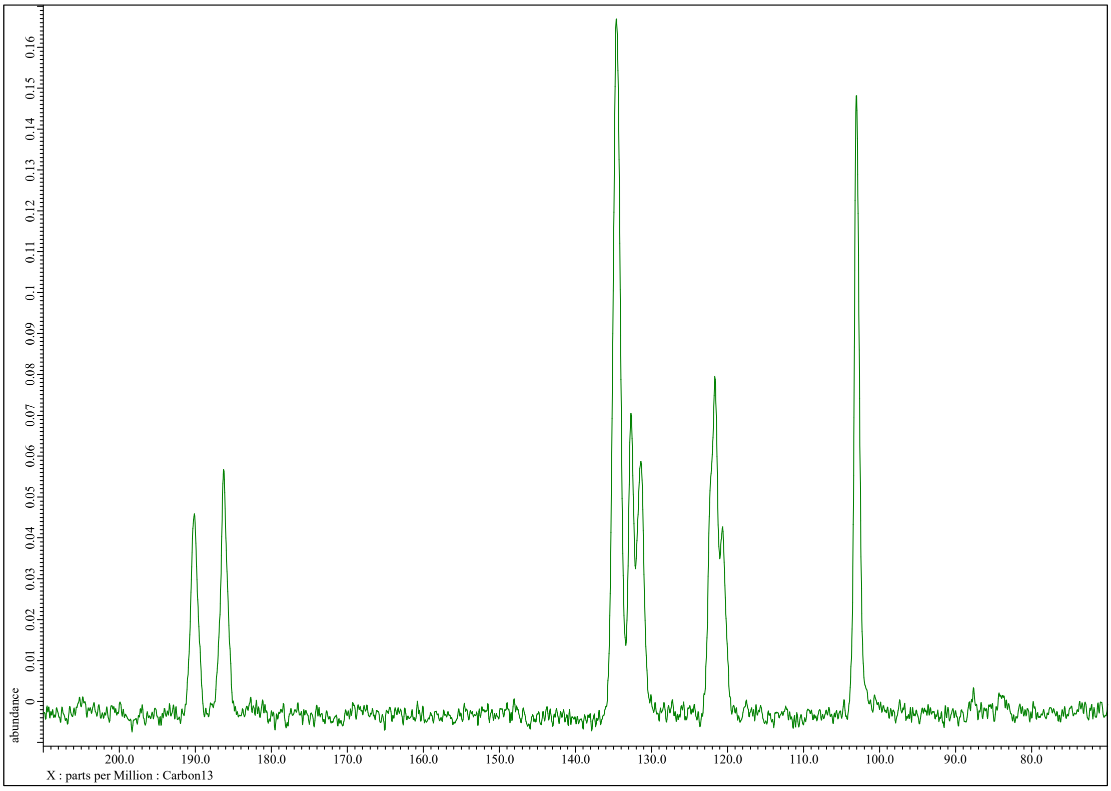

Figure S20 MAS ^{13}C solid-state NMR spectrum of **1** at 298 K.

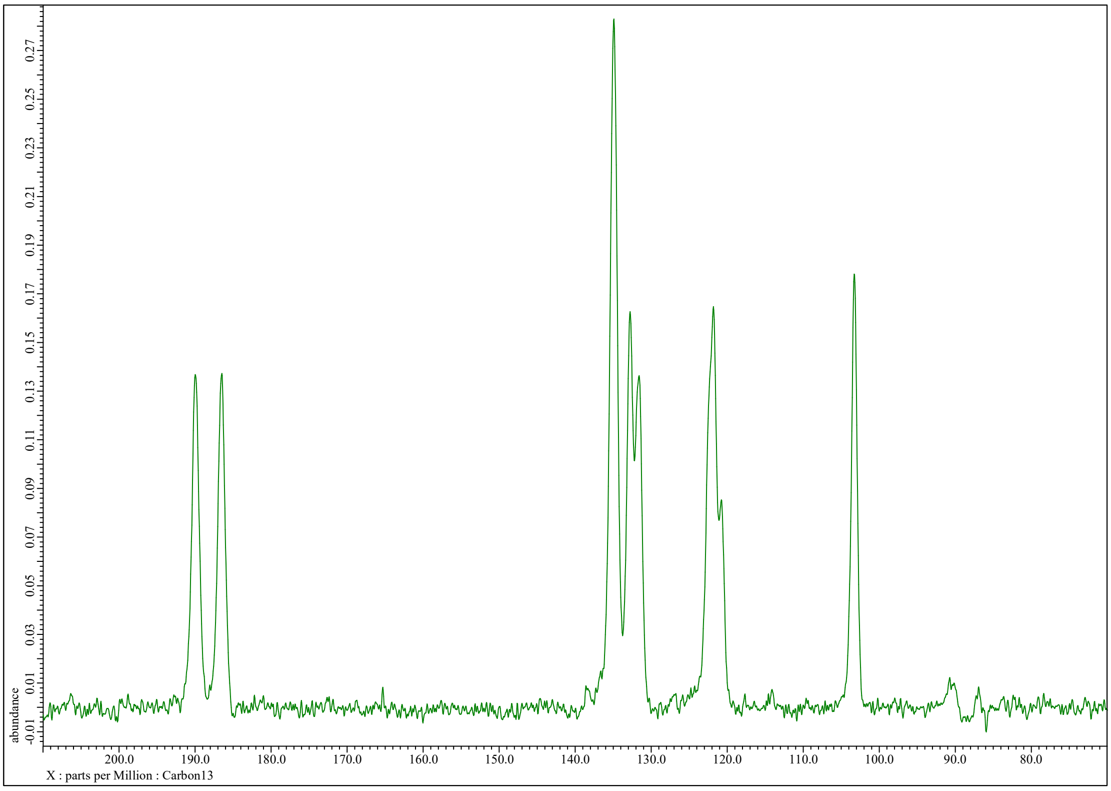

Figure S21 MAS ^{13}C solid-state NMR spectrum of **1** at 423 K.

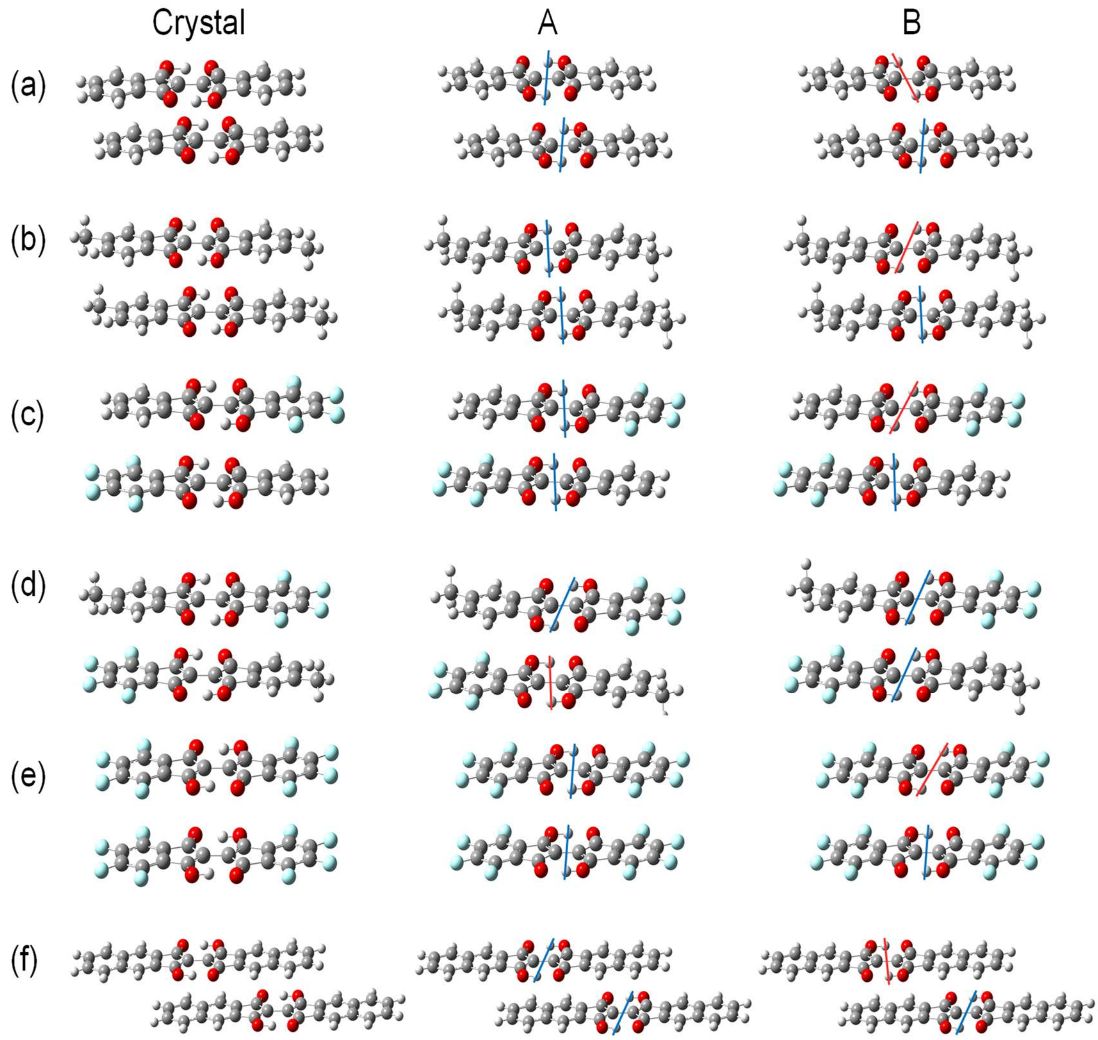

Figure S22 The molecular pairs of (a) **1**, (b) **2**, (c) **7**, (d) **8**, (e) **9**, and (f) **11** taken from the crystal structures which give the largest transfer integrals among the neighboring pairs (left column) and the generated tautomeric pairs in which the molecular conformations are optimized by DFT calculations, and the molecules are placed at the positions with the same center of mass and the molecular plane (right two columns). Two possible situations A and B are generated in term of the choice of the tautomers. The colored lines indicate the difference in proton positions.

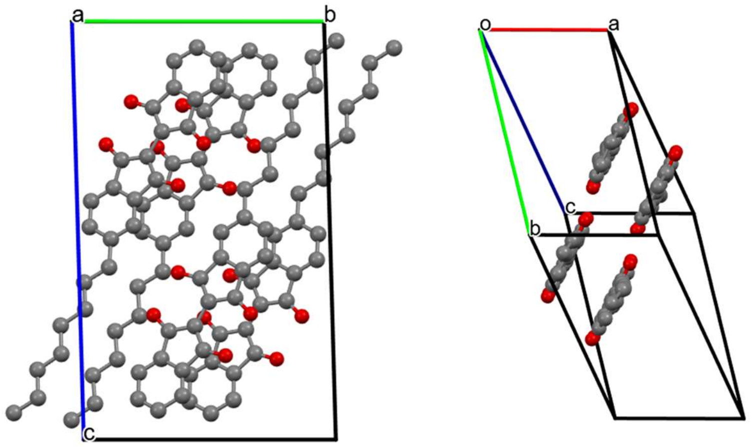

Figure S23 Crystal structure of **4**. ***a***: 7.2340(4); ***b***: 13.3280(8); ***c***: 21.6483(11) Å; ***α***: 86.340(4); ***β***: 81.601(4) **γ**: 75.391(5)°.

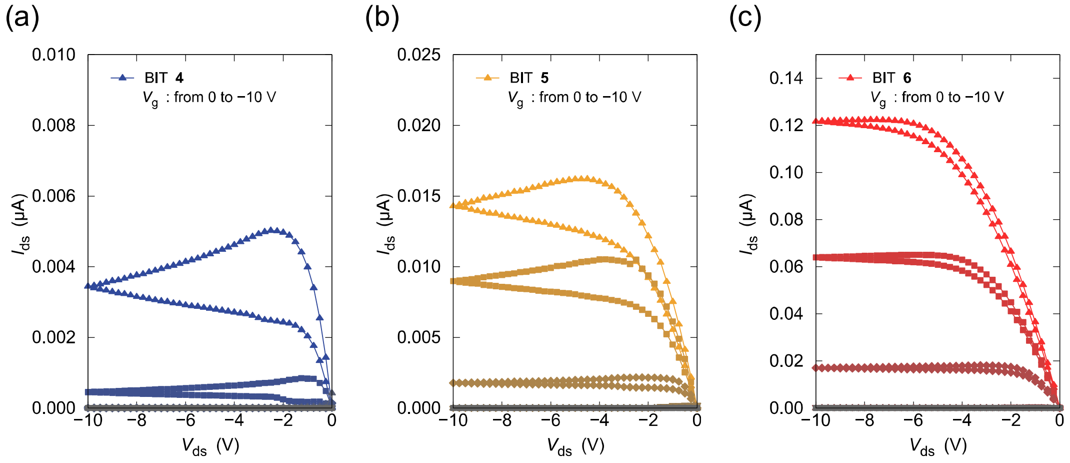

Figure S24 Output characteristics of OFET based on the films of (a) **4**, (b) **5**, and (c) **6**.

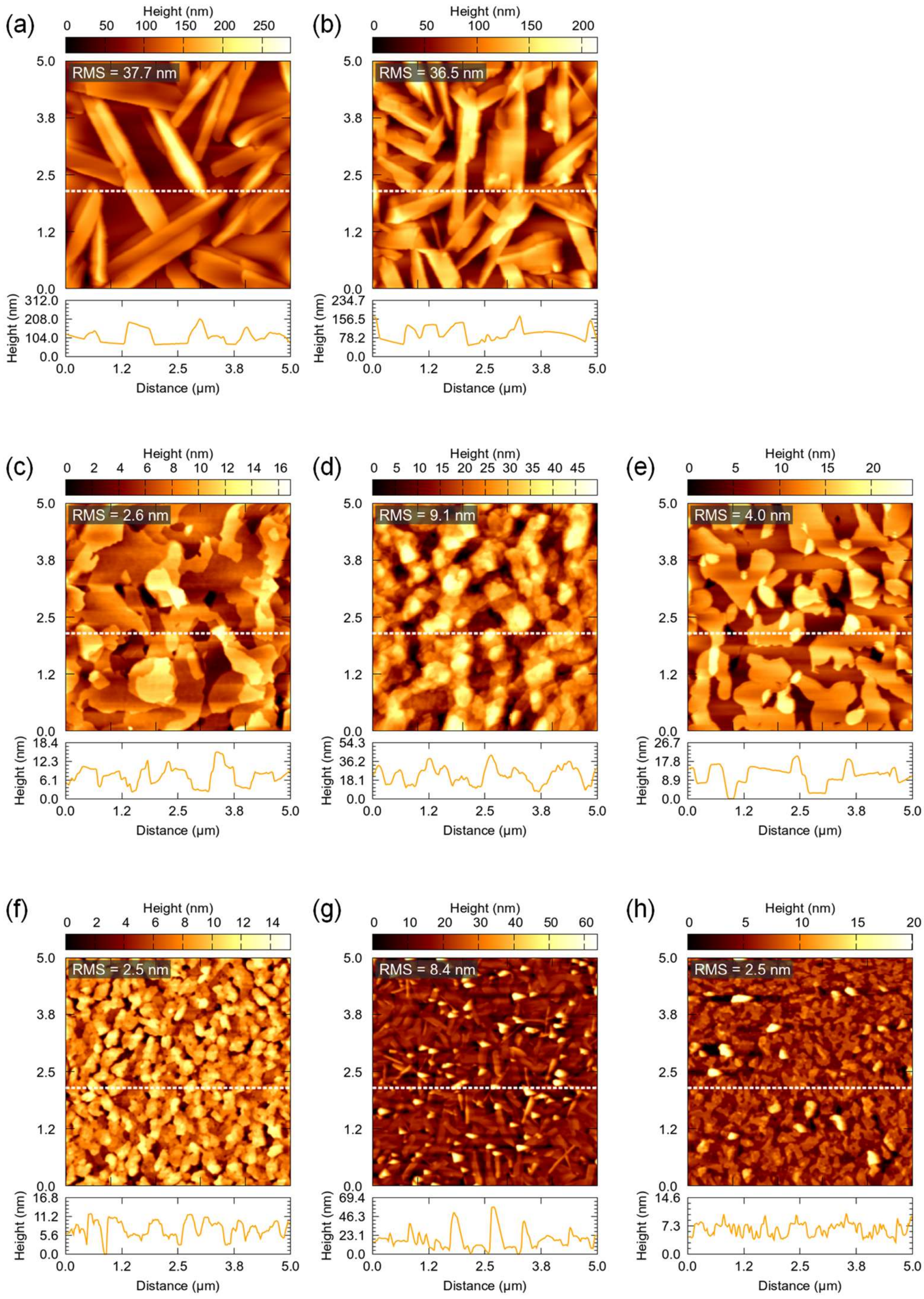

Figure S25 AFM topographic images of thermally evaporated films of (a) **1**, (b) **2**, (c) **4**, (d) **5**, (e) **6**, (f) **9**, (g) **11**, and (h) **12**. Image size: 5 ×5 μm. The bottom panels show line profiles of the topographic image at the positions indicated by dashed lines. RMS: root mean square.

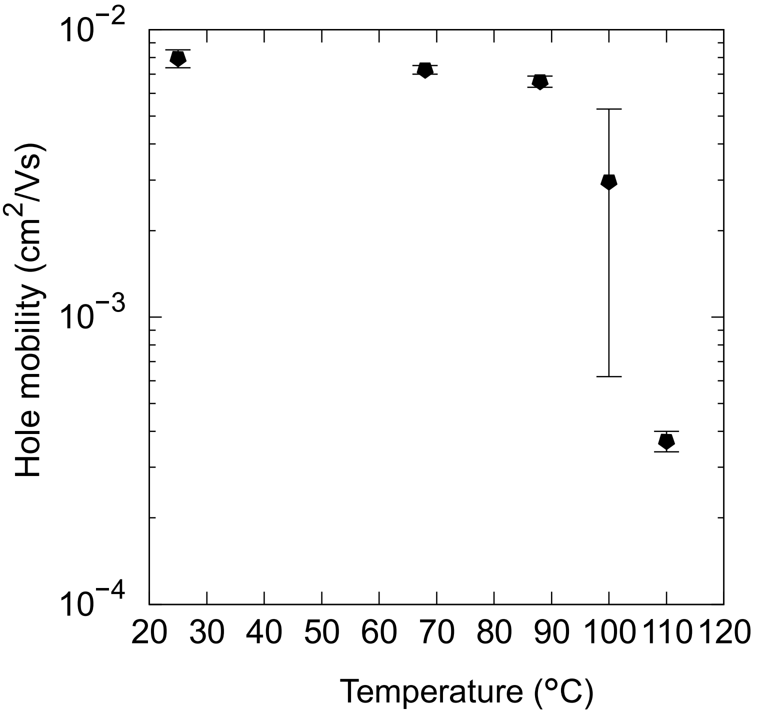

Figure S26 Temperature dependence of hole mobility of OFET based on the film of **6**.

Table S1 Energy levels of HOMO and LUMO for the tautomers.

Molecules	HOMO (eV)		LUMO (eV)	
	Tautomer a	Tautomer b	Tautomer a	Tautomer b
**1**	−5.63	−5.63	−2.88	−2.88
$\mathbf{2^{trans}}$	−5.50	−5.47	−2.73	−2.75
$\mathbf{2^{cis}}$	−5.48	−5.48	−2.74	−2.74
**3**	−5.56	−5.55	−2.82	−2.82
**7**	−6.02	−6.02	−3.38	−3.38
**8**	−5.95	−5.93	−3.33	−3.33
**9**	−6.42	−6.42	−3.68	−3.68
**10**	−5.62	−5.62	−2.84	−2.84
**11**	−5.62	−5.62	−2.73	−2.73
**12**	−5.56	−5.55	−2.75	−2.76

Table S2 Electronic energy of the tautomers and the barrier energy of the transient states for the neutral molecules.

	Electronic energy (Hartree)			Difference (a-b) (meV)	Barrier energy (meV)
	Tautomer a	Tautomer b	TS		
**1**	−993.118597	−993.118597	−993.110121	0	231
$\mathbf{2^{trans}}$	−1071.77538	−1071.774797	−1071.76704	−16	227
$\mathbf{2^{cis}}$	−1071.77509	−1071.775091	−1071.767031	0	219
**3**	−1032.44698	−1032.446732	−1032.43869	−7	225
**7**	−1390.15641	−1390.156408	−1390.149445	0	189
**8**	−1429.485	−1429.484795	−1429.478239	−5	184
**9**	−1787.19302	−1787.193017	−1787.184782	0	224
**10**	−1146.80152	−1146.801515	−1146.795341	0	168
**11**	−1300.48434	−1300.484337	−1300.477803	0	178
**12**	−1186.12992	−1186.129683	−1186.123465	−7	176

Table S3 Electronic energy of the tautomers and the barrier energy of the transient states for the radical cations.

	Electronic energy (Hartree)			Difference (a-b) (meV)	Barrier energy (meV)
	Tautomer a	Tautomer b	TS		
$\mathbf{1}^{\cdot+}$	−992.866654	−992.866654	−992.86226	0	120
$\mathbf{2}^{\mathbf{trans}\cdot+}$	−1071.52985	−1071.530574	−1071.525867	20	128
$\mathbf{2}^{\mathbf{cis}\cdot+}$	−1071.530199	−1071.530199	−1071.525838	0	119
$\mathbf{3}^{\cdot+}$	−1032.198291	−1032.198721	−1032.194402	12	118
$\mathbf{7}^{\cdot+}$	−1389.890806	−1389.890806	−1389.887256	0	97
$\mathbf{8}^{\cdot+}$	−1429.222829	−1429.223392	−1429.219827	15	97
$\mathbf{9}^{\cdot+}$	−1786.913749	−1786.913749	−1786.909595	0	113
$\mathbf{10}^{\cdot+}$	−1146.553395	−1146.553395	−1146.551285	0	57
$\mathbf{11}^{\cdot+}$	−1300.239442	−1300.239442	−1300.236219	0	88
$\mathbf{12}^{\cdot+}$	−1185.884787	−1185.885145	−1185.882629	10	68

Table S4 Electronic energy of the tautomers and the barrier energy of the transient states for the radical anions.

	Electronic energy (Hartree)			Difference (a-b) (meV)	Barrier energy (meV)
	Tautomer a	Tautomer b	TS		
$\mathbf{1}^{\cdot-}$	−993.191161	−993.191161	−993.17851	0	344
$\mathbf{2}^{\mathbf{trans}\cdot-}$	−1071.843986	−1071.843986	−1071.831234	0	347
$\mathbf{2}^{\mathbf{cis}\cdot-}$	−1071.843992	−1071.843992	−1071.831195	0	348
$\mathbf{3}^{\cdot-}$	−1032.517054	−1032.518127	−1032.504818	29	362
$\mathbf{7}^{\cdot-}$	−1390.246805	−1390.241177	−1390.230602	−153	441
$\mathbf{8}^{\cdot-}$	−1429.573801	−1429.56708	−1429.556894	−183	460
$\mathbf{9}^{\cdot-}$	−1787.29541	−1787.29541	−1787.283249	0	331
$\mathbf{10}^{\cdot-}$	−1146.875613	−1146.87312	−1146.861596	−68	381
$\mathbf{11}^{\cdot-}$	−1300.556828	−1300.556828	−1300.544513	0	335
$\mathbf{12}^{\cdot-}$	−1186.200135	−1186.201758	−1186.188177	44	370

Table S5 Energy and oscillator strength of the two lowest excited states for the molecules calculated by TD-DFT.

	Excited State	Energy (eV)	Oscillator strength	Contributions	Coefficient
**1**	1	2.0302	0	75(HOMO)→76(LUMO)	0.70485
	2	2.2422	0.0383	75(HOMO)→77(LUMO+1)	0.69855
**2**^{trans}	1	2.0436	0	83(HOMO)→84 (LUMO)	0.70448
	2	2.2538	0.0348	83(HOMO)→85 (LUMO+1)	0.69739
**2**^{cis}	1	2.0271	0	83(HOMO)→84 (LUMO)	0.70448
	2	2.239	0.0375	83(HOMO)→85 (LUMO+1)	0.69739
**3**	1	2.0338	0.0008	79(HOMO)→80 (LUMO)	0.68872
				79(HOMO)→81(LUMO+1)	0.149
	2	2.2498	0.0356	79(HOMO)→80(LUMO)	−0.14679
				79(HOMO)→81(LUMO+1)	0.68239
**7**	1	1.9855	0.0067	91(HOMO)→92(LUMO)	0.68177
				91(HOMO)→93 (LUMO+1)	0.17915
	2	2.2761	0.034	91(HOMO)→92(LUMO)	−0.17518
				91(HOMO)→93 (LUMO+1)	0.67677
**8**	1	1.9696	0.0096	95(HOMO)→96 (LUMO)	0.68493
				95(HOMO)→97 (LUMO+1)	0.16599
	2	2.2915	0.0285	95(HOMO)→96 (LUMO)	−0.16159
				95(HOMO)→97 (LUMO+1)	0.67959
**9**	1	1.9855	0.0067	91(HOMO)→92 (LUMO)	0.68177
				91(HOMO)→93 (LUMO+1)	0.17915
	2	2.2761	0.034	91 (HOMO)→92 (LUMO)	−0.17518
				91(HOMO)→93 (LUMO+1)	0.67677
**10**	1	2.1047	0.0055	88(HOMO)→89 (LUMO)	0.69464
				88(HOMO)→90 (LUMO+1)	0.10868
	2	2.3794	0.0415	88 (HOMO)→89 (LUMO)	−0.10174
				88(HOMO)→90 (LUMO+1)	0.68765
**11**	1	2.2414	0	101(HOMO)→102 (LUMO)	0.70341
	2	2.4575	0.0604	101(HOMO)→103(LUMO+1)	0.68544
				101(HOMO)→104(LUMO+2)	0.13787
**12**	1	2.124	0.0028	92(HOMO)→93(LUMO)	0.69925

	2	2.3719	0.0425	92(HOMO)→94 (LUMO+1)	0.69102

Table S6 Distances between π-planes and between the centroids of the molecules, and the displacement angle between the plane and the centroids.

Molecules	Distance between π-plane (Å)	Distance between centroids (Å)	Displacement angle between the plane and the centroids (°)
**1**	3.345	3.680	24.6
**2**	3.378	3.706	24.3
**7**	3.315	3.888	31.5
**8**	3.277	3.956	34.1
**9**	3.129	4.711	48.4
**11**	3.384	6.493	58.6
**12**[a]	3.410	11.358	72.5

[a] The centroids are defined for π-conjugated core

Table S7 Atomic distances (Å) at the central part of BIT molecules obtained from the DFT calculations at the B3LYP/6-311++G(d,p) level. Bond length differences between C-C and C=C and between C-O and C=O are also shown.

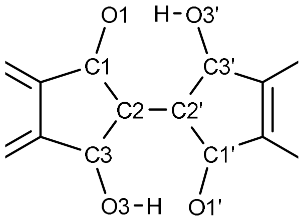

	**1**	Diff.	**2**	Diff.	**7**	Diff.	**8**	Diff.
C1-O1	1.236	0.075	1.237	0.074	1.232	0.075	1.232	0.075
C3-O3	1.311		1.311		1.307		1.306	
C1-C2	1.467	0.077	1.470	0.080	1.464	0.074	1.464	0.074
C2-C3	1.390		1.390		1.389		1.390	
C2-C2′	1.461		1.460		1.462		1.461	
C1′-O1′	1.236	0.075	1.237	0.074	1.236	0.074	1.237	0.075
C3′-O3′	1.311		1.311		1.310		1.311	
C1′-C2′	1.467	0.077	1.470	0.080	1.468	0.077	1.471	0.081
C2′-C3′	1.390		1.390		1.391		1.390	
O1-O3′	2.559		2.557		2.568		2.571	
O3-O1′	2.559		2.557		2.544		2.539	
O3-H	1.010		1.011		1.015		1.016	
O3′-H	1.010		1.011		1.006		1.005	

	**9**	Diff.	**11**	Diff.	**12**	Diff.
C1-O1	1.232	0.074	1.240	0.069	1.240	0.071
C3-O3	1.306		1.309		1.311	
C1-C2	1.465	0.075	1.465	0.068	1.465	
C2-C3	1.390		1.397		1.395	0.069
C2-C2′	1.463		1.460		1.460	
C1′-O1′	1.232	0.074	1.240	0.069	1.237	0.070
C3′-O3′	1.306		1.309		1.307	
C1′-C2′	1.465	0.075	1.465	0.068	1.465	0.071
C2′-C3′	1.390		1.397		1.395	
O1-O3′	2.554		2.538		2.538	
O3-O1′	2.554		2.538		2.555	
O3-H	1.010		1.016		1.011	
O3′-H	1.010		1.016		1.017	

Table S8 Atomic distances (Å) at the central part of **1** obtained from the single crystal structures at different temperatures. Bond length differences between C-C and C=C and between C-OH and C=O are also shown.

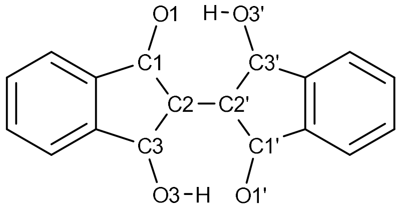

	**90 K**	Diff.	**298 K**	Diff.	**348 K**	Diff.	**373 K**	Diff.
C1-O1	1.251(2)	0.044	1.262(3)	0.014	1.264(3)	0.010	1.263(3)	0.015
C3-O3	1.295(2)		1.276(3)		1.274(3)		1.278(3)	
C1-C2	1.446(3)	0.052	1.424(3)	0.016	1.421(3)	0.011	1.420(3)	0.013
C2-C3	1.394(3)		1.408(3)		1.410(3)		1.407(3)	
C2-C2′	1.462(2)		1.458(3)		1.460(3)		1.461(3)	
C1′-O1′	1.249(2)	0.049	1.262(3)	0.015	1.262(3)	0.010	1.262(3)	0.011
C3′-O3′	1.298(2)		1.277(3)		1.272(3)		1.273(3)	
C1′-C2′	1.441(2)	0.039	1.421(3)	0.008	1.417(3)	0.004	1.417(3)	0.003
C2′-C3′	1.402(2)		1.413(3)		1.413(3)		1.414(3)	
O1…O3′	2.525(2)		2.523(2)		2.522(2)		2.525(2)	
O3…O1′	2.539(2)		2.536(2)		2.537(2)		2.537(2)	
O3-H	1.08(4)		1.28(4)		1.25(4)		1.28(4)	
O3′-H	1.04(4)		1.17(4)		1.18(4)		1.16(3)	

Table S9 Atomic distances (Å) at the central part of **7** obtained from the single crystal structures at different temperatures. Bond length differences between C-C and C=C and between C-OH and C=O are also shown.

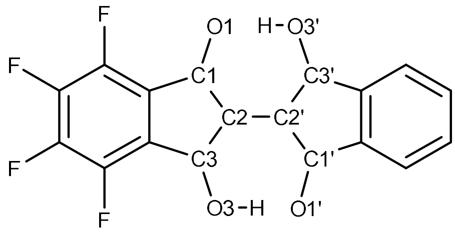

	**90 K**	Diff.	**298 K**	Diff.	**423 K**	Diff.
C1-O1	1.2327(14)	0.0771	1.2449(13)	0.0414	1.249(2)	0.030
C3-O3	1.3098(13)		1.2863(13)		1.279(2)	
C1-C2	1.4634(14)	0.0751	1.4412(14)	0.0398	1.426(2)	0.024
C2-C3	1.3883(15)		1.4014(14)		1.402(2)	
C2-C2′	1.4542(15)		1.4523(14)		1.458(2)	
C1′-O1′	1.2350(14)	0.0714	1.2473(13)	0.0370	1.274(2)	0.021
C3′-O3′	1.3064(13)		1.2843(13)		1.253(2)	
C1′-C2′	1.4608(14)	0.0717	1.4377(14)	0.0339	1.423(2)	0.017
C2′-C3′	1.3891(15)		1.4038(14)		1.406(2)	
O1…O3′	2.5628(12)		2.5563(13)		2.558(2)	
O3…O1′	2.5467(12)		2.5484(12)		2.550(2)	
O3-H	0.93(2)		1.03(3)		1.11(4)	
O3′-H	0.92(3)		1.07(3)		1.21(4)	

Table S10 Calculated transfer integrals (t^+) and reorganization energy (λ^+) for hole transport. t^+ are calculated for the molecular pairs taken from crystals and the tautomeric situations with the molecular structures optimized by DFT calculations as shown in Figure S22. λ^+ are calculated for the single molecules by DFT calculations.

Compounds	$\|t^+\|$ (meV)			Difference between A and B	λ^+ (meV)
	Crystal	A	B		
**1**	50	46	68	33%	406
**2**	106	100	79	21%	416
**7**	48	62	91	32%	442
**8**	12	33	24	38%	449
**9**	85	82	82	0%	484
**11**	55	57	67	15%	357

Table S11 Calculated transfer integrals (t^-) and reorganization energy (λ^-) for electron transport. t^- are calculated for the molecular pairs taken from crystals and the tautomeric situations with the molecular structures optimized by DFT calculations as shown in Figure S22. λ^- are calculated for the single molecules by DFT calculations.

Compounds	$\|t^-\|$ (meV)			Difference between A and B	λ^- (meV)
	Crystal	A	B		
**1**	19	13	18	38%	1074
**2**	67	78	56	28%	1125
**7**	44	45	55	18%	1221
**8**	27	78	57	27%	1239
**9**	1	5	7	29%	1122
**11**	54	56	52	7%	956

Table S12 Summary of d-spacings (Å) of diffractions peaks for the films of **4–6** in the out-of-plane direction.

	001	002	003	004	005	006	007	008
**4**	27.41	13.80	9.21	6.92	5.51	4.61	-	3.44
**5**	33.07	16.62	11.08	8.32	6.65	5.52	4.75	4.16
	22.16	11.08	7.38	5.52	4.44	-	-	-
**6**	38.02	18.90	12.75	9.57	7.66	6.39	-	4.79
	25.50	12.75	-	6.39	5.11	4.25	-	-

References

(1) Sheldrick, G. M. SHELXT - Integrated Space-Group and Crystal-Structure Determination. *Acta Crystallogr A Found Adv* **2015**, *71* (Pt 1), 3–8. https://doi.org/10.1107/S2053273314026370.

(2) Sheldrick, G. M. Crystal Structure Refinement with SHELXL. *Acta Crystallogr. B* **2015**, *71* (Pt 1), 3–8. https://doi.org/10.1107/S2053229614024218.

(3) Ito, Y.; Virkar, A. A.; Mannsfeld, S.; Oh, J. H.; Toney, M.; Locklin, J.; Bao, Z. Crystalline Ultrasmooth Self-Assembled Monolayers of Alkylsilanes for Organic Field-Effect Transistors. *J. Am. Chem. Soc.* **2009**, *131* (26), 9396–9404. https://doi.org/10.1021/ja9029957.

(4) Das, S.; Pramanik, A.; Fröhlich, R.; Patra, A. Facile Acid-Catalyzed Condensation of 2-Hydroxy-2,2′-Biindan-1,1′,3,3′-Tetrone with Phenols, Methoxyaromatic Systems and Enols. *Tetrahedron* **2004**, *60* (45), 10197–10205. https://doi.org/10.1016/j.tet.2004.09.004.

(5) Marminon, C.; Nacereddine, A.; Bouaziz, Z.; Nebois, P.; Jose, J.; Le Borgne, M. Microwave-Assisted Oxidation of Indan-1-Ones into Ninhydrins. *Tetrahedron Lett.* **2015**, *56* (14), 1840–1842. https://doi.org/10.1016/j.tetlet.2015.02.086.

(6) Wössner, J. S.; Grenz, D. C.; Kratzert, D.; Esser, B. Tuning the Optical Properties of Spiro-Centered Charge-Transfer Dyes by Extending the Donor or Acceptor Part. *Organic Chemistry Frontiers* **2019**, *6* (21), 3649–3656. https://doi.org/10.1039/c9qo01134j.
